# Pilot study of bempegaldesleukin in combination with nivolumab in patients with metastatic sarcoma

**DOI:** 10.1038/s41467-022-30874-8

**Published:** 2022-06-16

**Authors:** Sandra P. D’Angelo, Allison L. Richards, Anthony P. Conley, Hyung Jun Woo, Mark A. Dickson, Mrinal Gounder, Ciara Kelly, Mary Louise Keohan, Sujana Movva, Katherine Thornton, Evan Rosenbaum, Ping Chi, Benjamin Nacev, Jason E. Chan, Emily K. Slotkin, Hannah Kiesler, Travis Adamson, Lilan Ling, Pavitra Rao, Shreyaskumar Patel, Jonathan A. Livingston, Samuel Singer, Narasimhan P. Agaram, Cristina R. Antonescu, Andrew Koff, Joseph P. Erinjeri, Sinchun Hwang, Li-Xuan Qin, Mark T. A. Donoghue, William D. Tap

**Affiliations:** 1 Department of Medicine, Memorial Sloan Kettering Cancer Center, New York City, NY USA; 2 Department of Medicine, Weill Cornell Medical College, New York City, NY USA; 3 Parker Institute for Cancer Immunotherapy, Memorial Sloan Kettering Cancer Center, New York City, NY USA; 4 Marie-Josée and Henry R. Kravis Center for Molecular Oncology, Memorial Sloan Kettering Cancer Center, New York City, NY USA; 5 Department of Sarcoma Medical Oncology, Division of Cancer Medicine, The University of Texas MD Anderson Cancer Center, Houston, TX USA; 6 Laboratory of Chromatin Biology and Epigenetics, The Rockefeller University, New York City, NY USA; 7 Department of Pediatrics, Memorial Sloan Kettering Cancer Center, New York City, NY USA; 8 Department of Surgery, Memorial Sloan Kettering Cancer Center, New York City, NY USA; 9 Department of Pathology, Memorial Sloan Kettering Cancer Center, New York City, NY USA; 10 Program in Molecular Biology, Memorial Sloan Kettering Cancer, New York City, NY USA; 11 Department of Interventional Radiology, Memorial Sloan Kettering Cancer Center, New York City, NY USA; 12 Department of Radiology, Memorial Sloan Kettering Cancer Center, New York City, NY USA; 13 Department of Epidemiology and Biostatistics, Memorial Sloan Kettering Cancer Center, New York City, NY USA

**Keywords:** Sarcoma, Sarcoma

## Abstract

PD-1 blockade (nivolumab) efficacy remains modest for metastatic sarcoma. In this paper, we present an open-label, non-randomized, non-comparative pilot study of bempegaldesleukin, a CD122-preferential interleukin-2 pathway agonist, with nivolumab in refractory sarcoma at Memorial Sloan Kettering/MD Anderson Cancer Centers (NCT03282344). We report on the primary outcome of objective response rate (ORR) and secondary endpoints of toxicity, clinical benefit, progression-free survival, overall survival, and durations of response/treatment. In 84 patients in 9 histotype cohorts, all patients experienced ≥1 adverse event and treatment-related adverse event; 1 death was possibly treatment-related. ORR was highest in angiosarcoma (3/8) and undifferentiated pleomorphic sarcoma (2/10), meeting predefined endpoints. Results of our exploratory investigation of predictive biomarkers show: CD8 + T cell infiltrates and PD-1 expression correlate with improved ORR; upregulation of immune-related pathways correlate with improved efficacy; Hedgehog pathway expression correlate with resistance. Exploration of this combination in selected sarcomas, and of Hedgehog signaling as a predictive biomarker, warrants further study in larger cohorts.

## Abstract (web summary)

The activity of PD-1 blockade in patients with sarcoma has been modest so far. Here, the authors report the results of a pilot clinical trial to assess the efficacy and safety of bempegaldesleukin, a CD122-preferential interleukin-2 (IL-2) pathway agonist, in combination with the PD1 blockade (nivolumab) in patients with locally advanced or metastatic high-grade sarcoma.

## Introduction

Sarcomas are diverse malignancies comprising more than 100 subtypes of bone and soft tissue origin^1^. Each year, up to 16,000 patients are diagnosed in the United States^2^. In the metastatic setting, there are no good treatment options: front-line chemotherapy is associated with low objective response rates (ORR, averaging ~18%)^3,4^ and second-line treatments have lower efficacy still^5^. In other cancers, the use of monoclonal antibodies that block immune checkpoints such as programmed death 1 (PD-1; PDCD1) are effective alternative therapies to mobilize the immune system^6^, but in sarcoma their activity has been modest^7–10^. Studies that could elucidate this low response by characterizing the tumor microenvironment in sarcoma are limited.

High tumor mutational burden (TMB), tumor PD-L1 (CD274) expression, and increased tumor-infiltrating lymphocytes (TIL) are biomarkers of response to checkpoint inhibitors in multiple cancer types that have been found to be low across most sarcoma subtypes^11,12^. Prior studies in sarcoma have associated the presence of TlLs with improved clinical outcome^11,13–17^. These markers correspond to an immune-sensitive phenotype labeled “immune-hot.” In soft tissue sarcomas (STS) classified by gene expression analysis as immune-hot, with high expression of CD8 + T cells, tertiary lymphoid structures, and B cell markers, patients had improved response to PD-1 blockade^12^.

Novel approaches are needed to improve the effectiveness of PD-1 blockade while identifying predictive biomarkers of treatment response and resistance. We recently showed that combining talimogene laherparepvec via intratumoral injection with pembrolizumab in metastatic sarcoma led to immune conversion and correlated with improved clinical efficacy^10^. Bempegaldesleukin, a CD122-preferential interleukin-2 (IL-2) pathway agonist, is associated with increased proliferation and activation of TILs among patients with solid tumors, providing the rationale to explore this agent in sarcoma^18,19^.

We hypothesized that bempegaldesleukin would improve the efficacy of nivolumab PD-1 blockade. In this open-label, non-comparative pilot clinical trial (NCT03282344), we perform an integrated genetic, transcriptomic, and immunopathologic analysis to uncover the immune landscape in sarcoma at baseline and after treatment to differentiate which patients are most likely to respond. Here, we show that CD8 + T cell infiltrates and PD-1 expression correlate with improved ORR; that upregulation of immune-related pathways correlates with improved efficacy; and that Hedgehog pathway expression correlates with resistance.

## Results

### Patient cohort

A total of 84 patients with selected locally advanced or metastatic high-grade sarcoma were enrolled from October 6, 2017 to January 28, 2020 at Memorial Sloan Kettering Cancer Center (MSK) and MD Anderson Cancer Center (MDA). All patients received bempegaldesleukin 0.006 mg/kg and nivolumab 360 mg/kg as an intravenous (IV) infusion every 3 weeks, and treatment was continued until progressive disease (PD) or toxicity.

At study entry, patients’ mean age was 52 years (standard deviation: 17 years), 40 of 84 (48%) were female, 57 of 84 (68%) had an Eastern Cooperative Oncology Group (ECOG) performance status of 0, and 39 (46%) had received ≥3 lines of prior chemotherapy. Patients were divided into 9 cohorts predefined by histological subtype: alveolar soft part sarcoma (ASPS, *n* = 4), angiosarcoma (*n* = 10), conventional/dedifferentiated chondrosarcoma (*n* = 8; *n* = 2), leiomyosarcoma (LMS, *n* = 10), dedifferentiated liposarcoma (*n* = 10), osteosarcoma (*n* = 10), small blue round cell tumor (SBRCT) or synovial sarcoma (*n* = 6), undifferentiated pleomorphic sarcoma or high-grade myxofibrosarcoma (UPS/MFS, *n* = 10), and other (*n* = 14) (Table 1). Enrollment was completed in all cohorts other than ASPS and SRBCT/synovial sarcoma, which was terminated due to slow accrual.

**Table 1:** Patient characteristics.

	LMS	UPS, MFS	Chondrosarcoma	DDLPS	Osteo-sarcoma	Angiosarcoma	ASPS	SBRCT or synovial sarcoma	Other	Total
Patients (*n*)	10	10	10	10	10	10	4	6	14	84
Age, y										
Mean (SD)	60.5 (9.4)	66.0 (7.1)	56 (13.0)	59.2 (10.2)	39.4 (18.4)	55.7 (16.3)	27.5 (2.9)	38.6 (12.7)	46.1 (18.3)	51.8 (17.2)
Median (range)	58 (52–80)	69.5 (55–74)	57 (35–76)	58.5 (40–7)	36.5 (14–70)	64.5 (27–74)	26.5 (25–3)	38 (22–56)	48 (13–70)	56 (13–80)
ECOG PS										
0	7 (70%)	8 (80%)	5 (50%)	8 (80%)	5 (50%)	9 (90%)	2 (50%)	5 (83%)	8 (57%)	57 (68%)
1	3 (30%)	2 (20%)	5 (50%)	2 (20%)	5 (50%)	1 (10%)	2 (50%)	1 (17%)	5 (35%)	26 (31%)
2	0	0	0	0	0	0	0	0	1 (7.1%)	1 (1.2%)
Gender										
Female	8 (80%)	2 (20%)	4 (40%)	5 (50%)	4 (40%)	6 (60%)	2 (50%)	2 (50%)	7 (50%)	40 (48%)
Male	2 (20%)	8 (80%)	6 (60%)	5 (50%)	6 (60%)	4 (40%)	2 (50%)	4 (40%)	7 (50%)	44 (52%)
Prior therapies										
0	0 (0%)	1 (10%)	2 (20%)	0 (0%)	0 (0%)	2 (20%)	1 (25%)	0 (0%)	2 (14%)	8 (9.5%)
1	1 (10%)	1 (10%)	1 (10%)	1 (10%)	1 (10%)	2 (20%)	1 (25%)	0 (0%)	5 (36%)	13 (15%)
2	2 (20%)	4 (40%)	4 (40%)	4 (40%)	3 (30%)	3 (30%)	0 (0%)	0 (0%)	4 (29%)	24 (29%)
≥3	7 (70%)	4 (40%)	3 (30%)	5 (50%)	6 (60%)	3 (30%)	2 (50%)	6 (60%)	3 (21%)	39 (46%)

Data are *n* (%) unless specified.

*LMS* leiomyosarcoma, *UPS* undifferentiated pleomorphic sarcoma, *MFS* myxofibrosarcoma, *DDLPS* dedifferentiated liposarcoma, *ASPS* alveolar soft part sarcoma, *SBRCT* small blue round cell, *ECOG PS* Eastern Cooperative Oncology Group performance status.

**Fig. 1:**
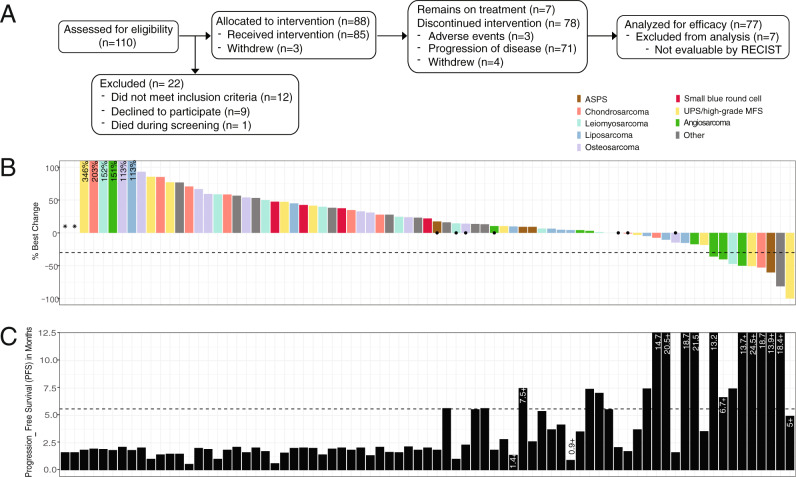
Response to the combination of nivolumab and bempegaldesleukin in patients with metastatic sarcoma. **A** CONSORT diagram. **B** Waterfall plot of overall best response (% change in sum of length measurements) of target lesions/nodes (RECIST v1.1; *n* = 77 patients evaluable for efficacy). Horizontal line represent cutoff for partial response (PR). Dot indicates achievement of response according to radiographic assessments despite classification as PD in non-target lesions (per RECIST v1.1). Two patients with progressive disease had target lesions that were not evaluable by RECIST. **C** Progression-free survival (PFS) for each patient (*n* = 77 patients). PFS is listed for patients without progression longer than 1 year and for patients who did not yet reach progression (indicated by + ). Dashed line indicates 24 weeks. ASPS alveolar soft part sarcoma, UPS undifferentiated pleomorphic sarcoma, MFS myxofibrosarcoma. Source data are provided as a Source Data file.

At the time of the data lock, 77 patients had discontinued study treatment and 7 patients remained on this combination; the most common reason for discontinuation was disease progression (Fig. 1A) while adverse events led to discontinuation in 3 (4%) patients. Thirty-four (40%) patients were alive with a median of 5 months of follow-up at the data lock date (February 24, 2020). Among the 50 deaths, 46 resulted from sarcoma and 4 were from unknown causes as patients were lost to follow-up.

### Efficacy analysis: response rates and survival

Seventy-seven patients were included in the efficacy analysis as 6 had discontinued study treatment prior to disease reevaluation and 1 had not reached their first response assessment at the time of data lock. Outcomes varied by histological subtype (Table 2, Fig. 1B). Median time to response by Response Evaluation Criteria in Solid Tumors (RECIST) was 3.7 months and median duration of response was 9.3 months. Median duration of treatment was 4.8 months (95% CI 3.2–6.1 months). Clinical benefit rate at 6 months ranged from 0% in osteosarcoma to 63% in angiosarcoma, while 67 (87%) evaluable patients experienced progressive disease (PD). Median progression-free survival (PFS) ranged from 1.8 months in LMS to 7.3 months in angiosarcoma (Fig. 1C, Table 2, Supplementary Fig. 1A). Median overall survival (OS) ranged from 5.9 months in the SBRCT tumor to not reached in both cohorts of ASPS and angiosarcoma cohorts (Supplementary Fig. 1B).

**Table 2:** Estimates of efficacy endpoints by subtype (*N* = 77).

	LMS	UPS, MFS	Chondrosarcoma	DDLPS	Osteo-sarcoma	Angio- sarcoma	ASPS	SBRCT or synovial sarcoma	Other
PR	1/10	2/10	1/10	0/10	0/10	3/8	1/4	0/5	1/10
Median DoR* (months)	3.8	14.8	12.9	NR	NR	5.9	11.8	NR	16.3
CBR at 6 months	20%	20%	20%	37.5%	0	62.5%	50%	0	10%
Median PFS (months)	1.8	2.4	1.8	3.9	2.0	7.3	2.6	2.0	2.1
Median OS (months)	6.9	9.2	5.1	21.7	6.3	NE	NE	5.9	12.0

*The data are censored at data freeze.

*LMS* leiomyosarcoma, *UPS* undifferentiated pleomorphic sarcoma, *MFS* myxofibrosarcoma, *DDLPS* dedifferentiated liposarcoma, *ASPS* alveolar soft part sarcoma, *SBRCT* small blue round cell, *PR* partial response, *DoR* duration of response, *CBR* clinical benefit rate, *NR* not reached, *NE* not evaluable

### Safety analysis

There were 84 patients who received at least one dose of therapy and were included in the safety analysis. All patients experienced ≥1 adverse event (AE); the most common grade 3 or 4 AEs were elevated lipase (*n* = 13; 10%), anemia (*n* = 8; 10%), elevated serum amylase (*n* = 7; 8%), hypertension (*n* = 6; 7%), pain (*n* = 6; 8%), and thromboembolic events (*n* = 4; 5%.)

All 84 patients also experienced ≥1 treatment-related adverse event (TRAE) of any grade (possibly, probably, and definitely related). The most common were fatigue (*n* = 84, 100%), fever (*n* = 84, 100%), rash (*n* = 63, 75%), pruritus (*n* = 60, 71%), arthralgia (*n* = 52, 62%), flu-like symptoms (*n* = 48, 57%), and chills (*n* = 41, 49%.) Grade 3 or 4 TRAEs occurred in 30 (35%) patients; the most common were elevated lipase (*n* = 5; 6%), elevated amylase (*n* = 3; 4%), and myalgia (*n* = 2; 2%) (Supplementary Table 1). There was one death possibly related to treatment. The patient stopped treatment due to disease progression, developed immunotherapy-associated pneumonitis shortly thereafter, and later presented with progressive dyspnea, ultimately expiring from respiratory failure.

### Immune cell populations evolve after treatment and define better responders

**Fig. 2:**
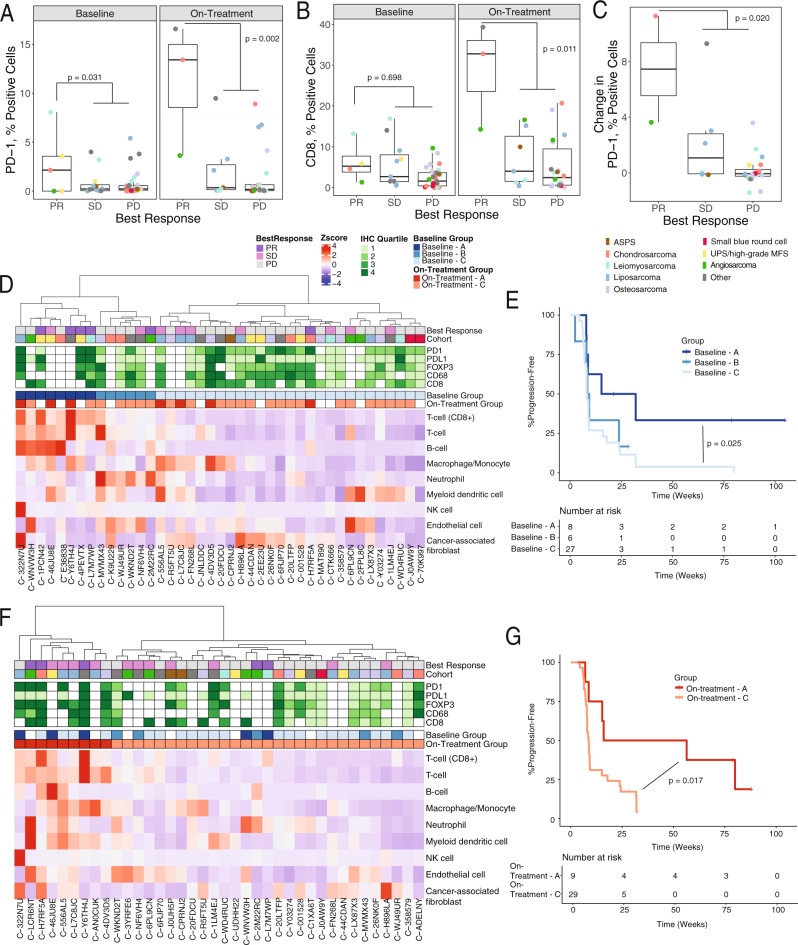
Immune cell content in on-treatment samples differentiates response. **A**–**C** Percent (**A**) PD-1-positive cells (Baseline n = 5 patients [PR], 12 [SD], 31 [PD]; On-Treatment n = 3 [PR], 7 [SD], 23 [PD]), (**B**) CD8-positive T cells (Baseline *n* = 4 patients [PR], 11 [SD], 32 [PD]; On-Treatment *n* = 3 [PR], 7 [SD], 22 [PD]), (**C**) change in PD-1-positive cells (*n* = 2 patients [PR], 6 [SD], 20 [PD]), as determined by IHC and categorized by best response and sample time point. **A**–**C** Colors indicate trial cohort. ASPS alveolar soft part sarcoma, UPS undifferentiated pleomorphic sarcoma, MFS myxofibrosarcoma. *P* values are nominal and derived from linear model of positive cells with ORR including sarcoma subtype as a covariate. Boxplot shows the median with hinges at 25th and 75th percentile with whiskers extending to smallest or largest value, no more than 1.5 times interquartile range from the hinges. All values are shown with points. **D**, **F** Immune markers in baseline (*n* = 41 patients) and on-treatment samples (*n* = 38 patients), respectively, clustered using Manhattan distance and Ward D clustering on the immune populations from RNA-seq (bottom heatmap), with color coding for best response (top row), quartile of percent positive cells for 5 immune markers as detected using IHC, and immune clusters according to the dendrogram. **E**, **G** Kaplan–Meier plot of progression-free survival of 3 and 2 immune clusters defined in **D** and **F**, respectively. Logrank *p* values shown for significant comparisons (Baseline *n* = 8 patients [A], 6 patients [B], *n* = 27 [C], On-Treatment *n* = 9 patients [A], On-Treatment *n* = 29 [C]). Source data are provided as a Source Data file.

Tumors from 61 patients were stained by immunohistochemistry (IHC) for various immune markers (Supplementary Fig. 1C, Supplementary Data 1) using matched specimens with both baseline and on-treatment samples. PD-1 expression at baseline was associated with ORR (linear model *p* = 0.031, covariate *p* = 4.7 × 10^−4^) while controlling for the effect of sarcoma histology (Fig. 2A). There was no association for baseline CD8, PD-L1, CD68, or FOXP3 markers (Fig. 2B, Supplementary Fig. 2A–C, Supplementary Data 2). However, at the on-treatment time point (cycle 2), both CD8 and PD-1 were associated with ORR, suggesting a potential change in tumor immune microenvironment in patients who attained a partial response (PR; linear model *p* = 0.01 and 0.002, covariate *p* = 4.6 × 10^−4^ and 7.3 × 10^−5^, respectively; Fig. 2A, B).

The matched specimen analysis showed that changes in PD-1+ cells between these time points was predictive of PR (linear model *p* = 0.020, covariate *p* = 4.3 × 10^−4^; Fig. 2C, Supplementary Fig. 2D–G, Supplementary Data 2).

Immune cell populations were quantified via Microenvironment Cell Populations (MCP)counter^20^ using RNA expression from 48 patients (41 baseline and 38 on-treatment biopsies) (Supplementary Fig. 1C). The reliability of this approach was confirmed by comparing this quantification to IHC of CD8 + T cells and CD68 + macrophages and monocytes; marker expression by IHC significantly correlated with MCPcounter-calculated immune cell abundance (Spearman’s *p* < 10^−7^, rho = 0.74 and 0.75, respectively; Supplementary Fig. 2H, I).

Independent clustering across immune cell populations quantified by MCPcounter on baseline samples classified tumors into three distinct groups with variation in response rates and PFS. Immune-hot tumors (Group A) were characterized by elevated CD8 + T cells; intermediate tumors (Group B) by high neutrophils and intermediate levels of other immune cells; and immune-cold tumors (Group C) by low levels of all immune cells. Patients with immune-hot tumors at baseline were significantly enriched for partial responders (Fisher’s test *p* value = 0.009) and had significantly longer PFS (logrank *p* = 0.025; Fig. 2D, E). Interestingly, 4/6 patients with partial response had immune-hot tumors at baseline. All on-treatment samples could be classified as immune-hot or immune-cold (Fig. 2F). Patients with immune-hot tumors in on-treatment samples also had higher response rates (PR or stable disease [SD]: 7/9; Fisher’s test *p* = 0.02) and improved PFS (Fig. 2G, logrank *p* = 0.017). The cell populations in baseline and on-treatment samples varied. For instance, T cells were consistently high at baseline and on-treatment in immune-hot sample while macrophages were more prevalent in immune-hot samples on-treatment versus at baseline.

### Expression of immune pathways and hedgehog signaling pathway differentiate patient response

**Fig. 3:**
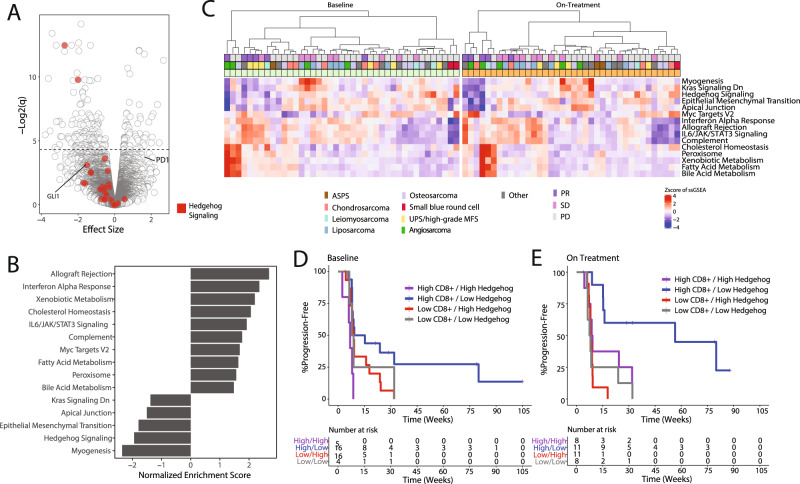
Differentially expressed pathways in partial responders. **A** Volcano plot of differential expression between partial responders (*n* = 7 patients) and non-responders (*n* = 41 patients). Model included trial cohort, patient, purity, and sample time point as covariates. Points represent genes. Genes in Hallmark hedgehog pathway are highlighted in red. **B** Top 10 upregulated and all 5 downregulated Hallmark pathways between partial responders (*n* = 7 patients) and non-responders (*n* = 41 patients). Analysis performed using fgsea in R. All pathways are significantly enriched (BH adjusted *p* < 0.05). **C** Heatmap of ssGSEA scores of baseline (*n* = 41 patients) and on-treatment (*n* = 38 patients) expression in individual patients for top enriched pathways in (**B**) using the leading-edge genes from the fgsea analysis. **D**, **E**. Kaplan–Meier plot of progression-free survival of patients divided into four groups depending on the amount CD8 + T cells and ssGSEA score of hedgehog pathway enrichment across cohort (high versus low, split by median). Scores derived from expression at baseline in (**D**) and on-treatment samples in (**E**). Note, cross-validation analysis does not output *p* values. Source data are provided as a Source Data file.

RNA-seq analysis identified 225 genes differentially expressed between patients with partial response (responders) versus non-responders across time points, of which 67 genes were upregulated (Fig. 3A, Supplementary Data 3). PD-1 was nominally differentially expressed with higher expression in patients with partial response (nominal *p* = 0.002, *q* value = 0.08). Interestingly, tumors of patients with SD had low *PD-1* expression at baseline, similar to patients with PD, but showed increased expression on treatment, suggesting tumor conversion due to therapy (*p* = 0.014; Supplementary Fig. 3A).

Gene set enrichment analysis (GSEA) was performed to assess shifts in expression across genes in a pathway^21^. Several immune-related pathways were upregulated in patients with PR, including IL6/JAK/STAT3 signaling and interferon alpha response (Benjamini-Hochberg [BH] adjusted *p* = 0.0096 for both); epithelial mesenchymal transition and Hedgehog signaling pathways were downregulated (BH adjusted *p* = 0.0021 for both) (Fig. 3B). Using hierarchical clustering of single-sample GSEA scores (ssGSEA), we found some clustering of partial responders at baseline but more defined clustering of patients following treatment, with one group in which 4 of 5 patients achieved PR and another with 7 of 9 patients receiving clinical benefit (Fig. 3C). Similar pathways were identified using an alternative differential expression model with additional covariates (Supplementary Fig. 3B). Furthermore, when we specifically compared patients with progressive disease to those with clinical benefit (partial response or stable disease), we identified similar pathways including interferon alpha response (BH adjusted *p* = 0.0016) and Hedgehog signaling (BH adjusted *p* = 0.0011) (Supplementary Fig. 3C).

Cross-validation GLM-NET of pathways and immune cell populations showed that Hedgehog signaling and CD8 + T cells, at baseline and on treatment, best predicted PFS; lower levels of Hedgehog signaling and higher levels of CD8 + T cells led to longer PFS (Fig. 3D,E). Neither measure was strongly associated with cohort (Supplementary Fig. 3D–G). Not all Hedgehog genes move uniformly across samples but we can see a general shift to higher expression among patients with progressive disease. This trend is clearer on-treatment (Supplementary Fig. 3H). Furthermore, while glioma-associated oncogene homolog 1 (*GLI1*), a transcription factor involved in Hedgehog signaling, was nominally differentially expressed in our overall model using all samples (Fig. 3A; nominal *p* = 0.004, *q* = 0.11), its higher expression in PD and lower expression in PR was more striking in on-treatment samples (two-sided *t*-test *p* = 0.011 [PR vs Others], 0.039 [PD vs Others]; Supplementary Fig. 3I).

### Genomic features of sarcomas

To determine if specific genomic alterations influenced either the immune content or expression of various pathways, whole exome sequencing was performed on samples derived from 67 patients, including 61 at baseline, 57 on-treatment, and 2 additional on-treatment biopsy specimens (Supplementary Fig. 1C). Consistent with previous sequencing studies in sarcoma^22^, the most common putative drivers were *TP53* mutations (40%), amplification of *CDK4* and *MDM2* loci (15%), *RB1* mutations (13%), amplifications in *NCOR1* or *MAP2K4* (12%) and homozygous deletions of *CDKN2A* (13%), among others (Supplementary Fig. 4A). Recurrent alterations identified were often unique to specific sarcoma subtypes; *CDK4*/*MDM2* amplifications were identified in all but one dedifferentiated liposarcoma, *ASPSCR1-TFE3* fusions in all patients with alveolar soft part sarcoma, and fusions involving *EWSR1* in all patients with SBRCT. No significant differences in ORR or PFS were correlated with genomic alterations.

Sequencing data were available at both baseline and on-treatment (cycle 2) for 51 patients. A median of 78% (IQR 68–87%) of mutations were shared across paired samples, while the majority of discordance could be explained by differences in tumor purity (Supplementary Fig. 4B). Of 35 patients with ≥1 driver mutation, 20 shared all driver mutations across all their samples (Supplementary Fig. 4C). Differences in the clonality of drivers between samples was evident in 13 of the 20 patients, likely representing tumor heterogeneity.

The 2 additional on-treatment samples were from: a palliative surgical resection of a treatment-responding fibrosarcoma (other cohort) in the setting of a joint infection, which shared 62% of mutations with the baseline sample, including 2 putative drivers in *GNAS* and *NF2*; and a progressing chondrosarcoma that shared 36% of mutations from baseline, including a putative oncogenic *IDH2* mutation and a distinct oncogenic *TP53* alteration (missense mutation at p.G334V rather than a splicing mutation at p.T125), suggestive of convergent evolution (Supplementary Fig. 4D).

Tumor mutation burden (TMB) across samples was relatively low compared with other tumor types (median 1.22 mut/Mb, range 0.03–4.61 mut/Mb)^23^. TMB was not significantly associated with ORR or PFS, as has been described in other solid tumors (two-sided *t*-test *p* = 0.46, logrank *p* > 0.08; Supplementary Fig. 5A, C)^24–26^. Not surprisingly, TMB was associated with sarcoma subtype: osteosarcoma tended to have higher TMB (median = 1.96, two-sided *t*-test *p* < 0.05) while liposarcoma tended to have lower TMB (median = 0.93, two-sided *t*-test *p* = 0.02; Supplementary Fig. 5B).

Fraction of genome altered (FGA), defined as the fraction of the genome where the total number of alleles differs from a balanced genome, was not associated with ORR or PFS (two-sided *t*-test *p* = 0.55, logrank *p* > 0.26; Supplementary Fig. 5D, F) but was significantly associated with sarcoma subtype, consistent with previous observations^22^. UPS displayed high FGA (two-sided t-test BH *p* = 0.03), while liposarcoma and SBRCT sarcoma had significantly lower FGA (two-sided *t*-test BH *p* = 0.03 and 4.5 × 10^−4^, respectively; Supplementary Fig. 5E).

Neoantigen load is correlated with response to checkpoint inhibitors in malignancies such as melanoma and non small cell lung cancer (NSCLC)^24,25,27^. In this study, there was no significant correlation between the number of expressed neoantigens at baseline (predicted using NetMHC and with expression confirmed by RNA-seq) compared to ORR (two-sided *t*-test, nominal *p* = 0.298; Supplementary Fig. 6A). Interestingly, to the contrary, on-treatment tumors with PR or SD expressed fewer neoantigens (two-sided *t*-test, BH *p* = 0.01; Supplementary Fig. 6B), which may reflect increased aberrant expression in progressing patients, as the mutations are largely concordant across time points. As expected, TMB is correlated strongly with neoantigen load, before and after considering expression (r^2^ = 0.9 and 0.7, respectively; Supplementary Fig. 6C, D).

While most *HLA* genotypes did not correlate with response, patients with the HLA-A-11-01 allele had worse PFS (nominal *p* = 0.013; Supplementary Fig. 6E). The significance of this finding is unknown and may warrant further study. Of note, a recent report in melanoma and NSCLC highlighted the role that HLA-I genotype plays in outcomes from checkpoint inhibition^28^. Although previous work has suggested that loss of heterozygosity (LOH) at HLA loci may contribute to response to immunotherapy^28,29^, we did not find that correlation. T-cell receptors (TCRs) on antigen-presenting cells have also been suggested to influence response to immunotherapy^30^. Intratumoral TCR clonality and diversity at baseline or on-treatment did not correlate with clinical outcome in our study (Supplementary Fig. 6F–M).

## Discussion

There remains an unmet clinical need to optimize immunotherapeutic strategies for patients with metastatic sarcoma. We performed this clinical trial to evaluate the efficacy of bempegaldesleukin and nivolumab in predefined histological cohorts and obtained serial biopsy specimens to help provide insight into predictive biomarkers of response and resistance. We identified variations in ORR based on the cohort; there were no responses in osteosarcoma, conventional chondrosarcomas, and dedifferentiated liposarcomas, while 3 of 8 (38%) patients with non-cutaneous angiosarcoma and 2 of 10 (20%) patients with UPS obtained durable responses, thereby meeting predefined study endpoints and suggesting that additional study may be warranted. The clinical efficacy in non-cutaneous angiosarcoma is surprising and interesting; efficacy seen in UPS remains consistent with previously published studies^7–9^. The trial design emphasized the importance of histology-specific cohorts because of the heterogeneity of this disease; this heterogeneity also highlights the need for larger cohorts to better define the clinical efficacy of this therapeutic combination and validate the status of Hedgehog signaling status as a predictive biomarker.

We sought to identify genomic and immune correlates of clinical benefit from this combination by conducting immunohistochemistry, whole exome sequencing, and RNA sequencing analyses. In our study cohorts, we did not identify any putative genetic drivers that correlated with response or resistance. Further, genomic markers such as TMB, neoantigen load, and loss of heterozygosity did not correlate with clinical benefit. Elevated TMB and resultant dominant UV signature mutations have been found to be potential predictive biomarkers of benefit in cutaneous, head, and neck angiosarcomas^31^. Despite the low TMB identified in our analyzed cohort, 3 of 8 (38%) patients with non-cutaneous angiosarcoma derived prolonged clinical benefit with this drug combination, suggesting that conventional genomic markers alone may not suffice to identify suitable patients.

The presence of increased numbers of CD8 + T cells and higher PD-1 expression (by IHC and gene expression) at baseline and on-treatment were associated with improved ORR and prolonged PFS, consistent with previous reports in other solid tumors^32,33^. At baseline, our clustering analysis identified three groups of tumors (Group A: “immune-hot”; Group B: intermediate with higher neutrophils; Group C: “immune-cold”). Myeloid cells, including neutrophils, have been shown to suppress T cell responses, providing a potential explanation for the predominance of response in Group A versus Groups B and C^34^. On-treatment analysis identified only 2 groups, A and C. Convergence into 2 groups presumably highlights the changes induced in the immune microenvironment during treatment. Notably, Petitprez et al. recently classified sarcomas into five phenotypes that correlated with ORR and OS^12^. While the clustering by Petitprez et al. was conducted in primary resected specimens, our cohort only includes treated metastatic specimens. Further, the variability of sarcoma subtypes and overall lower numbers within each subtype may also explain the differences between our results.

In sarcoma, bempegaldesleukin did not appear to improve efficacy to checkpoint blockade; other mechanisms may be driving resistance to immunotherapy. Our GSEA identified the upregulation of mesenchymal transition and Hedgehog signaling pathway expression as associated with lack of benefit. Hedgehog signaling has been implicated in the development of resistance to systemic therapies such as cytotoxic chemotherapy, targeted agents, and radiation therapy^35–37^. The Hedgehog signaling pathway can also regulate the immune response and aberrant Hedgehog signaling has been shown to drive tumor growth through a number of pathways including enhanced immunosuppressive mechanisms^38–40^. We have shown that the presence of CD8 + T cells and the reduced expression of the Hedgehog signaling pathway led to the best clinical outcome. Interestingly, in basal cell carcinoma, inhibition of Hedgehog signaling with vismodegib and sonidegib has led to reduction in tumor burden and durable clinical benefit for some patients^41,42^. In the setting of tumor regressions due to Hedgehog inhibitors, favorable changes to the immune microenvironment have been shown, including upregulation of MHC class I expression and infiltration of CD8 + T cells into tumors^43^. The interplay of Hedgehog signaling and the immune microenvironment warrants further investigation in sarcoma.

Limitations of this study include the low ORR across each cohort, which precluded robust correlative analyses. Further, the non-randomized nature limits the ability to identify the additive benefit of bempegaldesleukin. Sarcoma is a complex heterogeneous disease and each histological subtype can have variable behavior and response to systemic therapy, prompting the need to enroll patients onto cohorts based on subtype. Without well-defined biostatistical endpoints within each cohort, drawing definitive conclusions regarding efficacy is also not feasible. The goal instead was to identify a signal of efficacy that would potentially lead to expansions and further study. Similarly, our correlative studies identified interesting observations that will require additional evaluation in larger histology-specific cohorts.

This combination has limited activity in most sarcoma subtypes, other than in angiosarcoma where additional study may be warranted. Our data highlights the potential role of CD8 + T cell infiltration in driving immune responses to checkpoint inhibition, as has been described in other malignancies^32,33^. While upregulation of Hedgehog signaling seems to correlate with lack of efficacy, a predictive biomarker of immune conversion remains to be defined and further evaluation of the relevance of Hedgehog signaling status is necessary.

## Methods

### Study design and participants

This was a multi-center, open-label, non-randomized, non-comparative pilot trial conducted at MSK and MDA (ClinicalTrials.gov: NCT03282344). Eligible patients were ≥12 years and had advanced or metastatic sarcoma measurable per RECIST v1.1, ECOG performance status of 0 or 1, an estimated life expectancy of ≥3 months, previous receipt of ≥1 systemic therapy for metastatic disease (if applicable), and adequate kidney, liver, and bone marrow function. A sample size of 10 patients was planned for 7 of the histological cohorts (LMS, UPS/MFS, chondrosarcoma, DDLPS, osteosarcoma, angiosarcoma and ASPS), and a sample size of 15 patients was planned for the SBRCT/synovial sarcoma and other cohorts to account for their heterogeneity. A minimum of 28 days was required between any previous systemic therapy and initiation of bempegaldesleukin and nivolumab. Key exclusion criteria were active brain metastases or history of autoimmune diseases. Prior therapy with anti-PD-1 was permitted. The first participant enrolled on September 14, 2017, and the last enrolled on January 17, 2020.

Study protocol (Supplementary Note 1) was approved by review boards at MSK and MDA. The study was conducted according to the Declaration of Helsinki and the Guidelines for Good Clinical Practice. Each participant signed an IRB-approved, protocol-specific informed consent in accordance with federal and institutional guidelines. This study was monitored for accrual, safety, and the primary endpoint, at least twice annually, by the Data and Safety Monitoring Board, a standing committee at MSK. Since RECIST remains the standard approach to assess efficacy on therapeutic trials, in deviation from the protocol, we opted to not perform irRECIST reads as it would not change our interpretation of the data or alter our conclusions.

### Treatment and assessments

Patients received bempegaldesleukin 0.006 mg/kg and nivolumab 360 mg (flat dose) as an intravenous infusion every 3 weeks. Treatment was continued until progressive disease (PD) or toxicity.

Tumor assessments were performed at baseline and every 8 weeks thereafter and were not subject to central radiology review. Patients experiencing PD were permitted to continue study treatment beyond initial PD if the patient had evidence of clinical benefit and tolerated study treatment. Dose reductions were not permitted; however, dose interruptions for up to 6 weeks were allowed. If treatment was discontinued due to adverse events, patients were followed until disease progression or initiation of different therapy for >30 days after the last dose of bempegaldesleukin and nivolumab. Laboratory tests were performed at baseline and every 2–3 weeks as per the treatment schedule. Adverse events were graded according to the National Cancer Institute Common Terminology Criteria for Adverse Events (NCI CTCAE) version 4.0 during treatment and ≥30 days after treatment discontinuation. Mandatory biopsies prior to treatment and during week 3 were obtained in the same anatomic site unless not medically feasible. In addition, research blood tests were obtained at baseline and pretreatment on day 1 of cycles 8, 16, 24, 46, and at the time of progression.

### Outcomes

The primary endpoint was confirmed ORR within each histology cohort based on RECIST v1.1 during protocol-directed treatment. Confirmation of response was required 4 weeks following initial response. Confirmed ORR was estimated as the number of patients with a CR or PR divided by the number of evaluable patients. Secondary outcomes were toxicity, clinical benefit rate, duration of response, PFS, OS, and duration of treatment. Clinical benefit rate was defined as best objective status (CR, PR, or SD) at a given time point while receiving protocol treatment, divided by the number of patients receiving treatment at the same time point. Duration of response was defined as the time from first CR or PR to date of PD. PFS was defined as the time from start of treatment to date of PD or death. Patients who discontinued treatment for reasons other than PD, such as adverse events, were censored at the date of their most recent disease evaluation prior to receiving any later systemic treatment regimens. OS was calculated as the time between start of treatment and date of death. Patients lost to follow-up were censored for survival at the date last known to be alive. Patients remaining in active treatment were censored for duration of treatment on their most recent date of treatment. All analyses excluded any data collected beyond the date of withdrawal for patients having withdrawn consent. After discontinuation of protocol treatment, survival status was assessed every 3 months for a year after the last dose.

### Statistical analyses of clinical outcomes

Eligible patients who had initiated study treatment were considered evaluable for safety endpoints. Efficacy endpoints include only patients receiving ≥1 post-treatment assessment. In the cohorts with *n* = 10 patients, groups with two confirmed responses were considered worthy of further study, while in the cohorts with *n* = 15 patients (SBRCT/synovial sarcoma and other), groups with three confirmed responses were worthy of further study. Categorical data analyses and summary statistics were used to report adverse events. When patients were found ineligible after initiating study treatment, safety endpoints included only data prior to the date of ineligibility. The analysis of secondary endpoints included only eligible, treated patients. Kaplan–Meier methodology was used to estimate the distributions of all time to event endpoints. All patients included in the primary endpoint evaluation were also included in time-to-event endpoints. For all statistical estimates, 95% confidence intervals (CIs) were calculated. All statistical analyses were performed using statistical analysis system (SAS) R version 4.0.2.

### Whole-exome sequencing

Frozen tissues were weighed and 20–30 mg homogenized in RLT buffer before nucleic acids were extracted using the AllPrep DNA/RNA Mini Kit (QIAGEN catalog # 80204) according to the manufacturer’s instructions. RNA was eluted in nuclease-free water and DNA in 0.5X Buffer EB. Viably frozen cells were thawed and pelleted and incubated for ≥30 min in 360 μL Buffer ATL + 40 μL proteinase K at 55 °C. DNA was isolated with the DNeasy Blood & Tissue Kit (QIAGEN catalog # 69504) according to the manufacturer’s protocol with 1 h of incubation at 55 °C for digestion. DNA was eluted in 0.5X Buffer AE. After PicoGreen quantification and quality control by Agilent BioAnalyzer, 100–250 ng of DNA were used to prepare libraries using the KAPA Hyper Prep Kit (Kapa Biosystems KK8504) with 8 cycles of polymerase chain reaction (PCR). After sample barcoding, 100–500 ng of library were captured by hybridization using the xGen Exome Research Panel v1.0 (IDT) according to the manufacturer’s protocol. PCR amplification of the post-capture libraries was carried out for 12 cycles. Samples were run on a HiSeq 4000 or HiSeq 2500 in Rapid mode in a PE100 run, using the HiSeq 3000/4000 SBS Kit or HiSeq Rapid SBS Kit v2 (Illumina). Normal and tumor samples were covered to an average of 100X and 204X, respectively.

The resulting fastqs were aligned and processed using TEMPO^44^ (code for processing whole exome sequencing data can be found at https://github.com/mskcc/tempo). Briefly, reads were aligned using Burroughs-Wheeler Aligner (BWA)-MEM^45^ to the GRCh37 reference genome. Genome Analysis Toolkit (GATK)^46^ best practices were used for base recalibration. High contamination of exome sequencing led to the removal of three samples from two patients from this analysis.

Somatic genome variants were called using the union of Mutect2^47^ and Strelka2^48^. Variants were then filtered by several measures including variant allele frequency <0.05, tumor read depth of 20, tumor alternate read count of 3, and normal read depth of 10, along with filtering out repeated regions from RepeatMasker^49^ and variants that appear at allele frequencies >0.01 in GNOMAD^50^. Variants were considered shared between samples of the same patient if there was ≥1 supporting read in the other sample from the same patient. Variants were then annotated using OncoKB^51^ for oncogenicity. Variants classified as “predicted/likely/oncogenic” in OncoKB or that cause truncation of tumor suppressor genes by OncoKB were considered drivers. Truncating mutations were defined as those that create a stop codon or were an out-of-frame indel. Clonality differences between baseline and on-treatment samples were calculated by estimating the uncertainty of the baseline variant allele frequency using the Wilson 95% CI on the binomial probability. If the on-treatment variant allele frequency was not within the 95% CI, it was considered discordant. TMB was calculated by TEMPO as the number of non-synonymous mutations in coding regions covered by the IDT baits. TMB was considered at baseline in analyses unless otherwise specified.

Somatic copy number alterations were called using FACETS (Fraction and Allele-Specific Copy Number Estimates from Tumor Sequencing) v0.5.14^52^. Each tumor and matched normal pair was processed in a two-pass manner: an initial run for purity and ploidy estimation followed by a second run for focal event detection. Each fit was manually reviewed to minimize false positives and to evaluate the quality of the fit, leading to eight samples from seven patients being removed from the analysis. FGA and whole-genome doubling were calculated from the resulting FACETS using facets-suite V2^53^. Baseline FGA was used in analyses unless otherwise specified. In the oncoprint, amplifications in tumor suppressor genes and homozygous deletions in oncogenes were removed as likely passenger events.

HLA typing was performed using PolySolver^54^. Neoantigens were identified using NetMHC-pan^55^. They were considered expressed if ≥1 RNA-seq read was identified to support the mutated base. Correlation between TMB and number of neoantigens was performed in R using “cor.test”.

### Analysis of IHC

Paraffin-embedded tumor specimen obtained prior to and during treatment was evaluated by IHC for the following markers: PD-L1 (rabbit clone 28-8), CD8 (mouse clone C8/144B), CD68 (mouse clone KP1), FOXP3 (mouse clone 236 A/E7), Ki67 (clone MIB-1) and PD-1 (rabbit clone EPR48772) by Mosaic laboratories (catalog numbers in parenthesis; Mosaic labs does not supply dilutions, as they are proprietary). The PD-L1 and Ki-67 assays were evaluated by measuring the percentage of cells staining in the following fashion: 0 (no staining), 1 + (weak staining), 2 + (moderate staining) and 3 + (strong staining). To calculate the H score, the following formula was used: (3 x % cells staining at 3 + ) + (2 x % cells staining at 2 + ) + (1 x % cells staining at 1 + ). Other cell types were also evaluated using maximum staining intensity (Max SI) which included evaluation of normal adjacent tissue, endothelia, smooth muscle, fibroblasts, stroma, inflammatory cells, and nerve. Not all samples were able to be tested for all markers: 84 samples were tested for PD-1 (48 baseline, 33 on-treatment, 3 progression), 93 for PD-L1 (53 baseline, 37 on-treatment, 3 progression), 81 for CD8 (47 baseline, 32 on-treatment, 2 progression), 84 for FOXP3 (48 baseline, 34 on-treatment, 2 progression). P-values were determined by linear modeling including sarcoma subtype cohort as a covariate.

### RNA sequencing

After RiboGreen quantification and quality control by Agilent BioAnalyzer, 107–500 ng of total RNA with RIN values of 5.4–10 underwent polyA selection and TruSeq library preparation according to instructions provided by Illumina (TruSeq Stranded mRNA LT Kit, catalog # RS-122-2102), with 8 cycles of PCR. Samples were barcoded and run on a HiSeq 4000 in a PE100 run, using the HiSeq 3000/4000 SBS Kit (Illumina). An average of 43 million paired reads was generated per sample. Ribosomal reads represented 1.9–19% of the total reads generated and the percent of mRNA bases averaged 69%.

The resulting fastqs were processed using an in-house RNA sequencing pipeline. Briefly, fastqs were aligned using STAR 2.7.0^56^ to Ensembl v75^57,58^. The resulting bams were used for quality control by Picard^59^ and to identify fusions using the intersection of calls from FusionCatcher^60^ and Arriba^61^. Expression was quantified using Kallisto^62^ and summarized at the gene level using Enembl v75.

Normalized transcripts per million (TPM) were calculated using Sleuth (sleuth_to_martix). Immune populations were quantified using the R package “immunedeconv”^63^. Within this package, we used MCPcounter^20^, from which Z-scores were calculated from the quantification of each population. Correlation between IHC values and those derived from MCPcounter was performed in R using “cor.test”. These scores were used for hierarchical clustering of samples at each time point using Manhattan distance and Wald D clustering to classify the overall immune infiltration into each tumor. Differences in PFS were evaluated by log-rank test.

First model to identify differentially expressed genes in Sleuth:^64^

${{{{{\rm{Expression}}}}}} \sim {{{{{\rm{TrialCohort}}}}}}+{{{{{\rm{SampleTimepoint}}}}}}+{{{{{\rm{PatientID}}}}}}+{{{{{\rm{Purity}}}}}}+{{{{{\rm{PartialResponder}}}}}}$ Where TrialCohort is sarcoma subtype, Sample Timepoint is either baseline, on treatment, or progression; Patient ID allows for multiple samples from the same patient; and PartialResponder is yes or no according to whether the patient met criteria for partial response. Purity was derived from FACETS (in exome data) except for four samples that did not have an acceptable FACETS fit and for which maximum variant allele frequency was used instead. Two samples did not have corresponding exome data and so were removed from this differential expression analysis. This model was chosen after considering several covariates, including cohort, sample time point, patient, purity, age at study entry, sex, number of prior treatments, relative amount of cancer fibroblasts (calculated from MCPcounter) and batch. To explain partial response, we used stepwise Akaike Information Criterion (AIC) to identify the optimal covariates^65^; these included batch, cohort and patient. Batch and cohort could not be modeled together because they produced a computationally singular system. We considered the linear model defined by stepwise AIC and found that while cohort and patient contributed significant effects on modeling partial response (*p* < 0.05), batch did not (*p* > 0.3).To gain further confidence in our results, we performed a linear model using “lm” in R. We modeled the log_2_ of the normalized TPM that was used for the immune deconvolution above. We limited the genes considered to those that passed sleuth filtering, which filters genes that do not have at least five estimated counts in at least 47% of samples. To compare partial responders and non-partial responders, we used the model

$${{{{{\rm{Expression}}}}}} \sim {{{{{\rm{PartialResponder}}}}}}+{{{{{\rm{TrialCohort}}}}}}+{{{{{\rm{SampleTimepoint}}}}}}+{{{{{\rm{PatientID}}}}}}+{{{{{\rm{Purity}}}}}}+{{{{{\rm{TrialBatch}}}}}}+{{{{{\rm{AgeAtStudyEntry}}}}}}+{{{{{\rm{Sex}}}}}}+{{{{{\rm{\#PriorTreatments}}}}}}+{{{{{\rm{CancerFibroblasts}}}}}}$$

We used the rank of genes from this model to perform GSEA and confirm results from the previously discussed model that resulted from sleuth filtering (Supplementary Fig. 3B).

A Wald test was used to identify differentially expressed genes between samples from partial responders and those from other patients. A gene was considered differentially expressed if *q* < 0.05. We utilized a similar model to compare samples with progressive disease and those with clinical benefit (including samples with partial response or stable disease). In this model, we replace “PartialResponder” with “ProgressiveDisease,” such that genes with a positive effect size are more highly expressed in samples with progressive disease.

Pathway enrichment was performed on Hallmark pathways^66^ pulled using R package msigdb (version 7.2.1)^65^. GSEA was performed using the R package fgsea^21^ on genes ranked by effect size (beta) from the above model. Minimum size was set to 15 and maximum was set to 600, and 10,000 permutations were performed. Figure 3B shows the top 10 significant pathways with a positive or negative normalized enrichment score. The R package GSVA^67^ was used to calculate ssGSEA values for each sample to cluster samples based on expression of enriched pathways. Heatmap shows ssGSEA calculated on only genes in the leading edge for each pathway, as found by fgsea analysis.

The model best associated with PFS was found using cv.glmnet from glmnet R package (version 4.1)^68^. Immune population quantification via MCPcounter and ssGSEA scores shown in Fig. 3C were combined in a Cox proportional hazards model to predict PFS for baseline and on-treatment samples, separately. The minimum lambda was used. Figure 3D, E were created using the same values for CD8 + cells and Hedgehog signaling as used in the model, but the high vs low samples were identified within each time point.

### TCR sequencing

After RiboGreen quantification and quality control by Agilent BioAnalyzer, 11–259 ng of total RNA were prepared using the Immunoverse TCR-HS α/δ/β/γ Kit, for Illumina (ArcherDX catalog # DB0219) according to the manufacturer’s instructions. Briefly, cDNA was synthesized using TCR-specific priming for reverse transcription. Molecular barcode adapters were ligated to cDNA fragments and multiplex PCR with primers targeting the CDR3 sequence of interest was used for enrichment and library preparation. Barcoded samples were pooled equimolar and sequenced on a MiSeq, NextSeq 500, or NovaSeq 6000 in a PE150 run, using the MiSeq Reagent Kit v3 (300 cycles), NextSeq 500/550 Mid Output Kit v2.5 (300 cycles), or NovaSeq 6000 S3 Reagent Kit (300 Cycles) (Illumina). Each sample yielded on average 3.2 M reads and fastq files were uploaded to the Archer Analysis bioinformatics suite for processing.

Archer delivered trimmed and deduplicated fastqs, which were then used to quantify TCR diversity for the 4 TCRs analyzed by the platform (*α*, *β*, *γ*, and *δ*). The MiXCR pipeline was used to process, align, assemble, and export clones for each TCR type separately^69^. Next, the VDJtools pipeline^70^ was utilized to calculate basic statistics including TCR diversity. Differences in TCR diversity were compared by two-sided *t*-test between each response group (PR, SD, or PD) vs. others.

### Reporting summary

Further information on research design is available in the Nature Research Reporting Summary linked to this article.

## Supplementary information

Supplementary Information

Description of Additional Supplementary Files

Supplementary Data 1

Supplementary Data 2

Supplementary Data 3

Reporting Summary

## Data Availability

De-identified exome sequencing data from patients treated at Memorial Sloan Kettering Cancer Center have been deposited in the NCBI dbGaP archive under accession number phs001783. De-identified exome sequencing data from patients treated at MD Anderson Cancer Center and all de-identified RNA sequencing and TCR sequencing data have been deposited in the NCBI dbGaP archive under accession number phs002852. The data are available under controlled access, which can be obtained through dbGaP upon request. The raw sequencing data from 3 patients are not deposited in dbGaP because they did not consent to future use. De-identified individual participant-level clinical data will be made available upon request to the corresponding author. Mapping of each sample, along with IHC values, can be found in Supplementary Data 1. The study protocol is available as Supplementary Note 1 in the Supplementary Information file. The source data are provided in the Source Data file and can also be found at https://github.com/mskcc/ImmunoSarc. The remaining data are available within the Article and Supplementary Information. Source data are provided with this paper.

## Source data

Source Data

## Code availability

Code for processing RNA-seq data and for subsequent analysis throughout the manuscript can be found at https://github.com/mskcc/ImmunoSarc.
