# Supplementary material for: Pilot study of bempegaldesleukin in combination with nivolumab in patients with metastatic sarcoma
Source: Nat Commun. 2022 Jun 16;13:3477. doi: 10.1038/s41467-022-30874-8 (PMC9203519; doi:10.1038/s41467-022-30874-8)
Supplement: Supplementary file 1 — Supplementary Information [file 41467_2022_30874_MOESM1_ESM.pdf]

Supplementary Table 1. Treatment-related adverse events (possible, probable and definitively related) (N=84)

CTCAE v4.03 AE Term	Grade 1-2	Grade 3	Grade 4	Grade 5
Abdominal pain	3 (4%)	1 (1%)	-	-
Acute coronary syndrome	-	1 (1%)	-	-
Acute kidney injury	-	2 (2%)	-	-
Alanine aminotransferase increased	19 (23%)	-	-	-
Alkaline phosphatase increased	9 (11%)	-	-	-
Allergic reaction	6 (7%)			
Amylase increased	7 (8%)	3 (4%)		
Anemia	21 (25%)	1 (1%)	-	-
Anorexia	11 (13%)	-	-	-
Arthralgia	52 (62%)	1 (1%)	-	-
Arthritis	4 (5%)	1 (1%)	-	-
Aspartate aminotransferase increased	17 (20%)	1 (1%)	-	-
Atrial fibrillation	1 (1%)			
Bilirubin increased	2 (2%)			
Bursitis	2 (2%)			
Chills	41 (49%)	-	-	-
Colitis	1 (1%)	1 (1%)	-	-
Constipation	2 (2%)	-	-	-
Cough	15 (18%)	-	-	-
Creatinine increased	2 (2%)			
Dehydration	1 (1%)	1 (1%)	-	-
Diarrhea	13 (15%)	-	-	-
Diplopia	1 (1%)			
Dry eye	2 (2%)			
Dry mouth	6 (7%)	-	-	-
Dry skin	7 (8%)	-	-	-
Dysgeusia	3 (4%)			
Dysphasia	1 (1%)			
Dyspnea	2 (2%)	-	-	-
Eczema	1 (1%)			
Edema	8 (10%)	-	-	-
Electrocardiogram QTC interval prolonged	4 (5%)	-	-	-
Eosinophilia	2 (2%)	-	-	-
Erythema	1 (1%)			
Fall	1 (1%)			
Fatigue	84 (100%)	-	-	-
Fever	84 (100%)	1 (1%)	-	-
Floaters	1 (1%)			
Flu-like symptoms	48 (57%)	-	-	-
Gastroesophageal reflux disease	1 (1%)	-	-	-
Generalized muscle weakness	2 (2%)	-	-	-
Ground glass opacity	1 (1%)	-	-	-
Heart failure	1 (1%)	-	-	-

CTCAE v4.03 AE Term	Grade 1-2	Grade 3	Grade 4	Grade 5
Hemorrhoids	1 (1%)			
Hoarseness	1 (1%)			
Hypertension	4 (5%)	1 (1%)		
Hyperthyroidism	3 (4%)			
Hypomagnesemia	5 (6%)	-	-	-
Hypophosphatemia	6 (7%)	1 (1%)	-	-
Hypotension	8 (10%)	2 (2%)	-	-
Hypothyroidism	6 (7%)			
Infusion reaction	2 (2%)	-	-	-
Insomnia	1 (1%)	-	-	-
Lipase increased	1 (1%)	5 (6%)	-	-
Malaise	19 (23%)	-	-	-
Mucositis	5 (6%)			
Myalgia	41 (49%)	2 (2%)	-	-
Nasal congestion	5 (6%)			
Nausea	26 (31%)	-	-	-
Neck pain	-	-	-	-
Neutrophil count decreased	6 (7%)	1 (1%)	-	-
Pain	9 (11%)	1 (1%)	-	-
Palmar-plantar erythrodysesthesia syndrome	2 (2%)			
Pancreatitis	2 (2%)			
Paresthesia	4 (5%)			
Parotitis	1 (1%)	1 (1%)	-	-
Peripheral neuropathy	5 (6%)	-	-	-
Phlebitis	1 (1%)			
Platelet count decreased	11 (13%)	-	-	-
Pleural effusion	1 (1%)	-	-	-
Pleuritic pain	2 (2%)			
Pneumonitis	1 (1%)	1 (1%)	-	-
Pneumothorax	1 (1%)	-	-	-
Presyncope	1 (1%)			
Proteinuria	1 (1%)			
Pruritus	60 (71%)	-	-	-
Rash maculo-papular	63 (75%)	1 (1%)	-	-
Respiratory failure	-	-	-	1 (1%)
Sinus tachycardia	1 (1%)	-	-	-
Sore throat	1 (1%)	-	-	-
Tendonitis	1 (1%)	-	-	-
Throat constriction	1 (1%)	-	-	-
Tremor	1 (1%)	-	-	-
Urinary retention	2 (2%)	-	-	-
Vomiting	12 (14%)	-	-	-
Syncope	-	1 (1%)	-	-
Watery eyes	1 (1%)	-	-	-
White blood cell decreased	3 (4%)	-	-	-

Frequency reported as n (%).

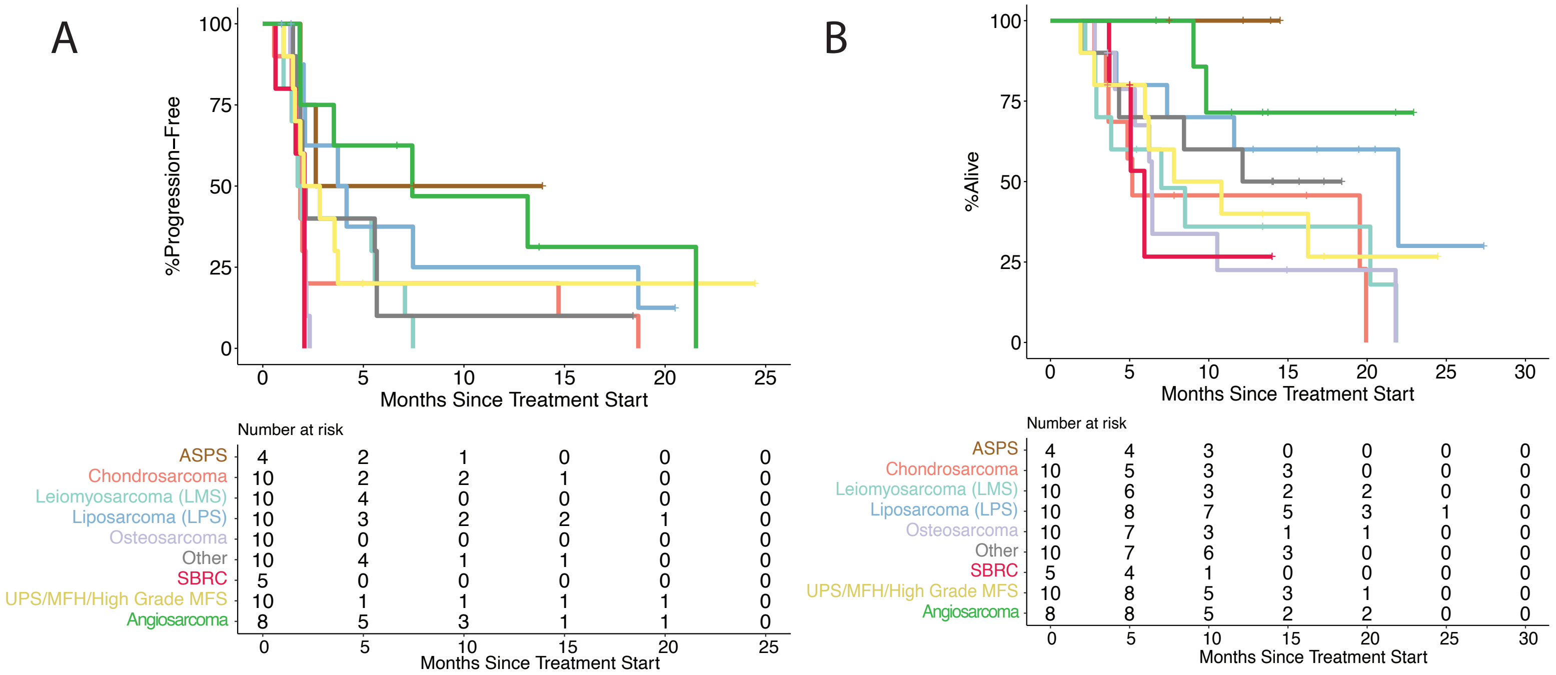

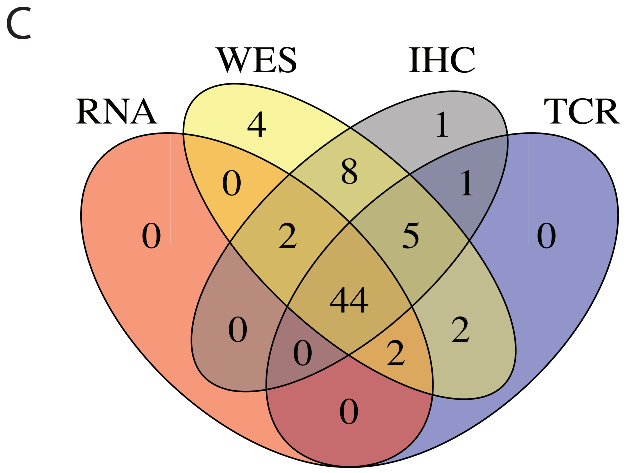

Supplementary Figure 1. Progression-free and overall survival. A–B, Kaplan-Meier estimates of the distribution of (**A**) progression-free survival and (**B**) overall survival (OS). **C.** Venn diagram illustrating analysis of samples by various methods. RNA-seq, RNA sequencing; WES, whole exome sequencing; TCR, T cell receptor sequencing. Source data are provided as a Source Data file.

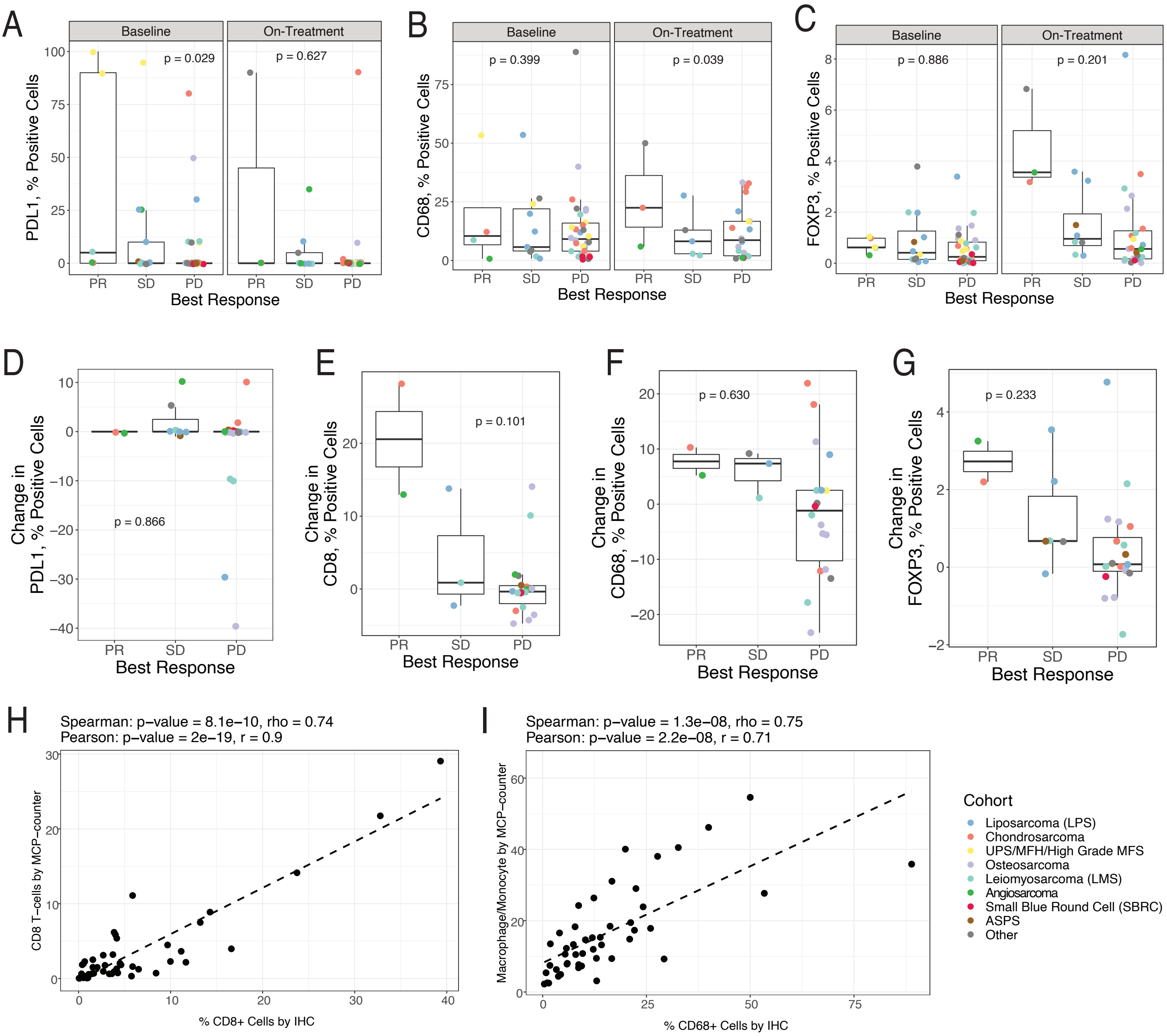

Supplementary Figure 2. Non-predictive immune markers and immune cell abundance clustering. A-C. Proportion of cells positive for (**A**) PD-L1 (Baseline n = 5 patients [PR], 13 [SD], 35 [PD], On-Treatment n = 3 [PR], 9 [SD], 25 [PD]), (**B**) CD68 (Baseline n = 4 patients [PR], 11 [SD], 30 [PD], On-Treatment n = 3 [PR], 5 [SD], 21 [PD]), and (**C**) FOXP3 (Baseline n = 5 patients [PR], 12 [SD], 31 [PD], On-Treatment n = 3 [PR], 8 [SD], 23 [PD]) by IHC according to best response and sample time point. **D-G**. Change between on-treatment and baseline in proportion of cells positive for (**D**) PD-L1 (n = 2 patients [PR], 7 [SD], 22 [PD]), (**E**) CD8 (n = 2 patients [PR], 3 [SD], 18 [PD]), (**F**) CD68 (n = 2 patients [PR], 3 [SD], 18 [PD]), and (**G**) FOXP3 (n = 2 patients [PR], 6 [SD], 20 [PD]), by IHC. P-values are nominal and were derived from a linear model of positive cells with ORR that included sarcoma subtype as a covariate. Boxplot shows the median with hinges at 25th and 75th percentile with whiskers extending to smallest or largest value, no more that 1.5 times interquartile range from the hinges. All values are shown with points. **H-I**. Correlation between IHC marker expression (x-axis) and immune cell content as derived by MCPcounter for (**H**) CD8 (n = 51 samples) and (**I**) CD68 (n = 47 samples). Source data are provided as a Source Data file.

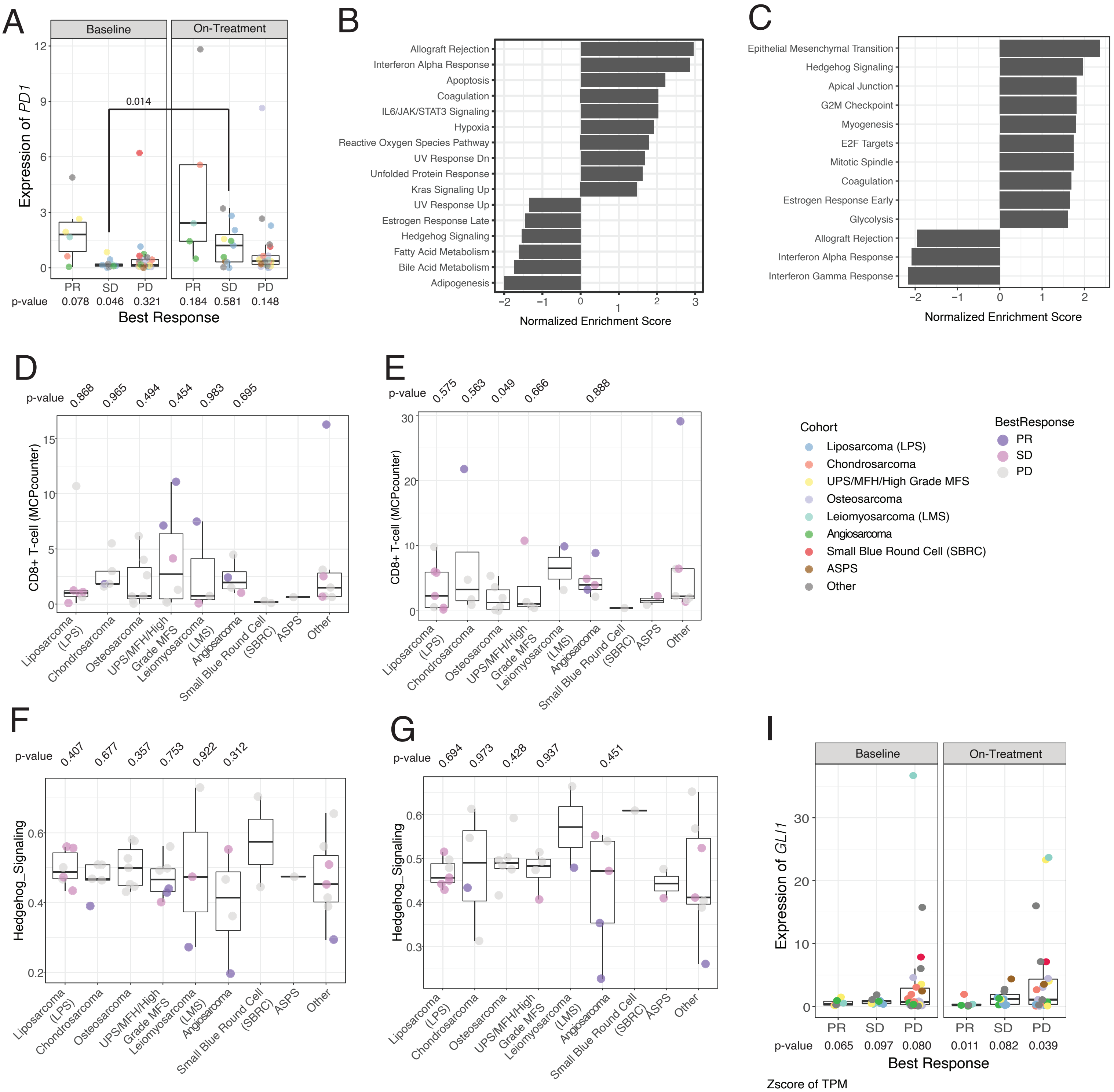

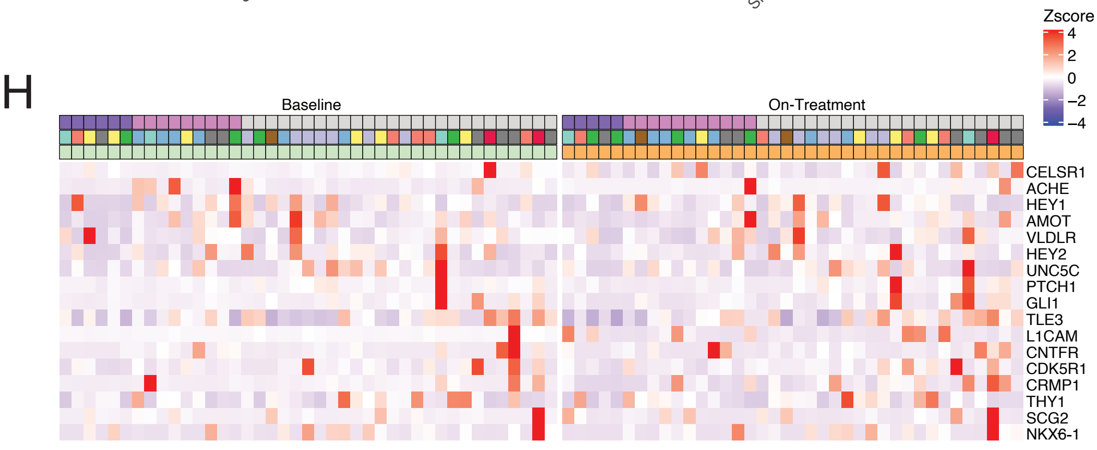

Supplementary Figure 3. Expression patterns differ by response and conversion. A. Expression (in transcripts per kilobase million [TPM]) of PD1 across responses and sample time points. P-values below graph are calculated by two-sided t-test vs. all other samples. p-value between time points of the same best response are shown in graph for SD (PR p-value = 0.324, PD p-value = 0.327). **B.** Top 10 upregulated and all 6 downregulated Hallmark pathways between patients with partial response and those without using the linear model with all possible covariates. Analysis performed using fgsea in R. All pathways are significantly enriched (BH adjusted p < 0.05). **C.** Top 10 upregulated and all 3 downregulated Hallmark pathways between patients with progressive disease and those with clinical benefit (PR and SD). Analysis performed using fgsea in R. All pathways are significantly enriched (BH adjusted p < 0.003). **D-E.** CD8+ T cells by MCPcounter by subtype at baseline (**D**) and on-treatment (**E**). **F-G.** Hedgehog signaling enrichment score via ssGSEA by subtype at baseline (**F**) and on-treatment (**G**). p-values calculated by two-sided t-test vs all other subtypes. Subtype "Other" and subtypes with fewer than 3 samples were not tested. **H.** Heatmap of Z-score of TPM for each gene in the leading edge of the Hedgehog pathway in the comparison of responders to non-responders, as found by fgsea analysis. Samples are ordered by overall response and separated by time point (Baseline n = 41 patients, On-Treatment n = 38 patients). **I.** Expression in TPM of GLI1 across responses and sample time points. P-values below graph are calculated by two-sided t-test vs all other samples. The same number of samples was used in tests for (**A**) and (**I**) (Baseline n = 6 samples [PR], 9 [SD], 26 [PD], On-Treatment n = 5 samples [PR], 11 [SD], 22 [PD]). The same number of samples was used in tests for (**D**) and (**F**) (n = 6 samples [Liposarcoma], 5 [Chondrosarcoma], 7 [Osteosarcoma], 6 [UPS], 3 [Leiomyosarcoma], 4 [Angiosarcoma]) and in tests for (**E**) and (**G**) (n = 7 samples [Liposarcoma], 4 [Chondrosarcoma], 6 [Osteosarcoma], 4 [UPS], 5 [Angiosarcoma]). Boxplots in (**A**), (**D-G**), and (**I**) show the median with hinges at 25th and 75th percentile with whiskers extending to smallest or largest value, no more that 1.5 times interquartile range from the hinges. All values are shown with points. Source data are provided as a Source Data file.

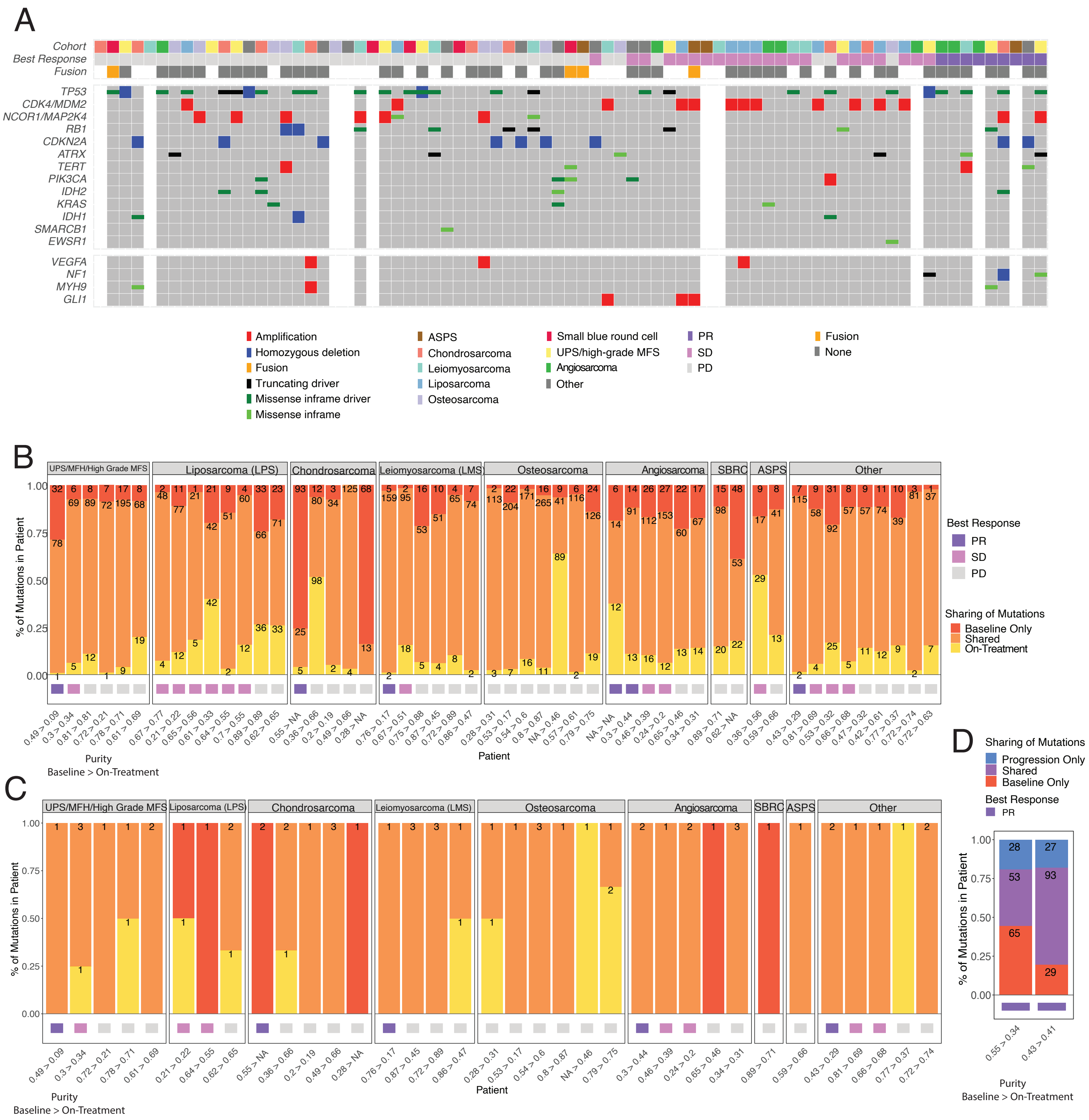

Supplementary Figure 4. Agreement between baseline and on-treatment sequencing. A. Oncoprint showing the union of genomic alterations across samples of 77 patients. Fusion status shows only fusions that are indicative of sarcoma subtype. Genes shown were selected based on outside evidence of impact on sarcoma and prevalence of alteration in this cohort. Four genes separated below are found in Hallmark Sonic Hedgehog pathway. Empty positions indicate missing data. ASPS, alveolar soft part sarcoma; UPS, undifferentiated pleomorphic sarcoma; MFS, myxofibrosarcoma. **B.** Percent shared mutations between baseline and on-treatment samples of the same patient. Numbers of mutations indicated on each bar. **C.** Percent shared driver mutations between baseline and on-treatment samples of the same patient. Numbers of mutations indicated on each bar. **D.** Percent shared mutations between baseline and progression of the two patients with progression samples. For (**B**), (**C**), and (**D**), numbers of mutations are indicated on each bar and purity is derived from FACETS where purity of "NA" indicates samples for which FACETS fails to quantify it. UPS, undifferentiated pleomorphic sarcoma; MFS, myxofibrosarcoma; SBRCT, small blue round cell tumor; ASPS, alveolar soft part sarcoma. Source data are provided as a Source Data file.

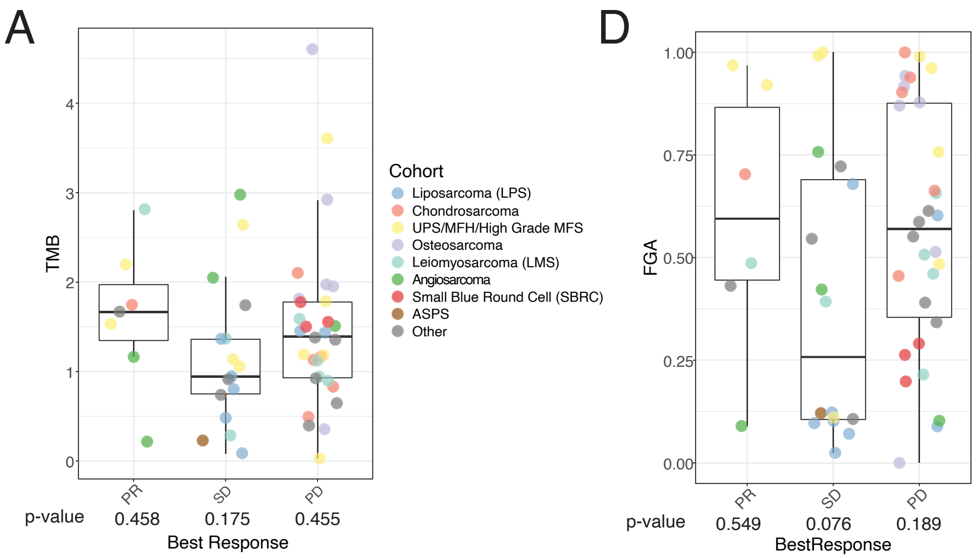

Supplementary Figure 5. Tumor mutation burden (TMB) and fraction of genome altered (FGA) differ according to sarcoma subtype but not treatment response. A. TMB according to best response (n = 7 samples [PR], 17 [SD], 31 [PD]). **B.** TMB according to sarcoma subtype (n = 8 samples [liposarcoma], 7 [chondrosarcoma], 8 [osteosarcoma], 10 [UPS], 8 [leiomyosarcoma], 6 [angiosarcoma], 3 [SBRC]). *C.* Kaplan-Meier plot comparing PFS across patients with varying TMB at baseline. **D.** FGA according to best response (n = 6 samples [PR], 16 [SD], 30 [PD]). **E.** FGA according to sarcoma subtype (n = 8 samples [liposarcoma], 7 [chondrosarcoma], 8 [osteosarcoma], 9 [UPS], 7 [leiomyosarcoma], 5 [angiosarcoma], 3 [SBRC]). **F.** Kaplan-Meier plot comparing PFS across patients with varying FGA at baseline. In (**A**) and (**D**), patients with PD by non-target lesions are not included. In (**A**), (**B**), (**D**) and (**E**), nominal p values are shown as calculated by two-sided t-test vs. all other subtypes. Subtypes with fewer than 3 samples were not tested. Boxplots in (**A-B**) and (**D-E**) show the median with hinges at 25th and 75th percentile with whiskers extending to smallest or largest value, no more that 1.5 times interquartile range from the hinges. All values are shown with points. Logrank p-values between each quartile are shown below (**C**) and (**F**). UPS, undifferentiated pleomorphic sarcoma; MFS, myxofibrosarcoma; ASPS, alveolar soft part sarcoma. Source data are provided as a Source Data file.

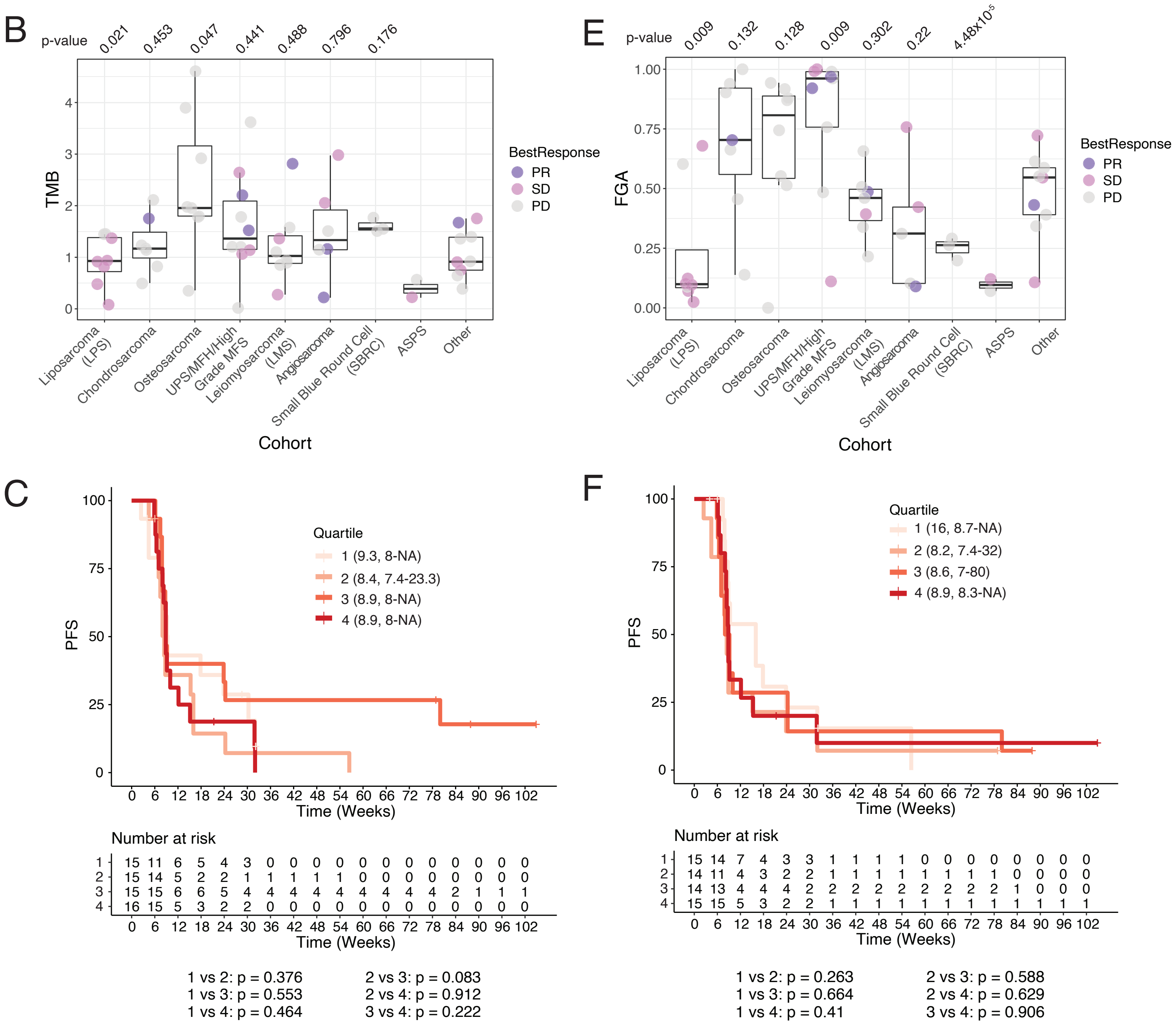

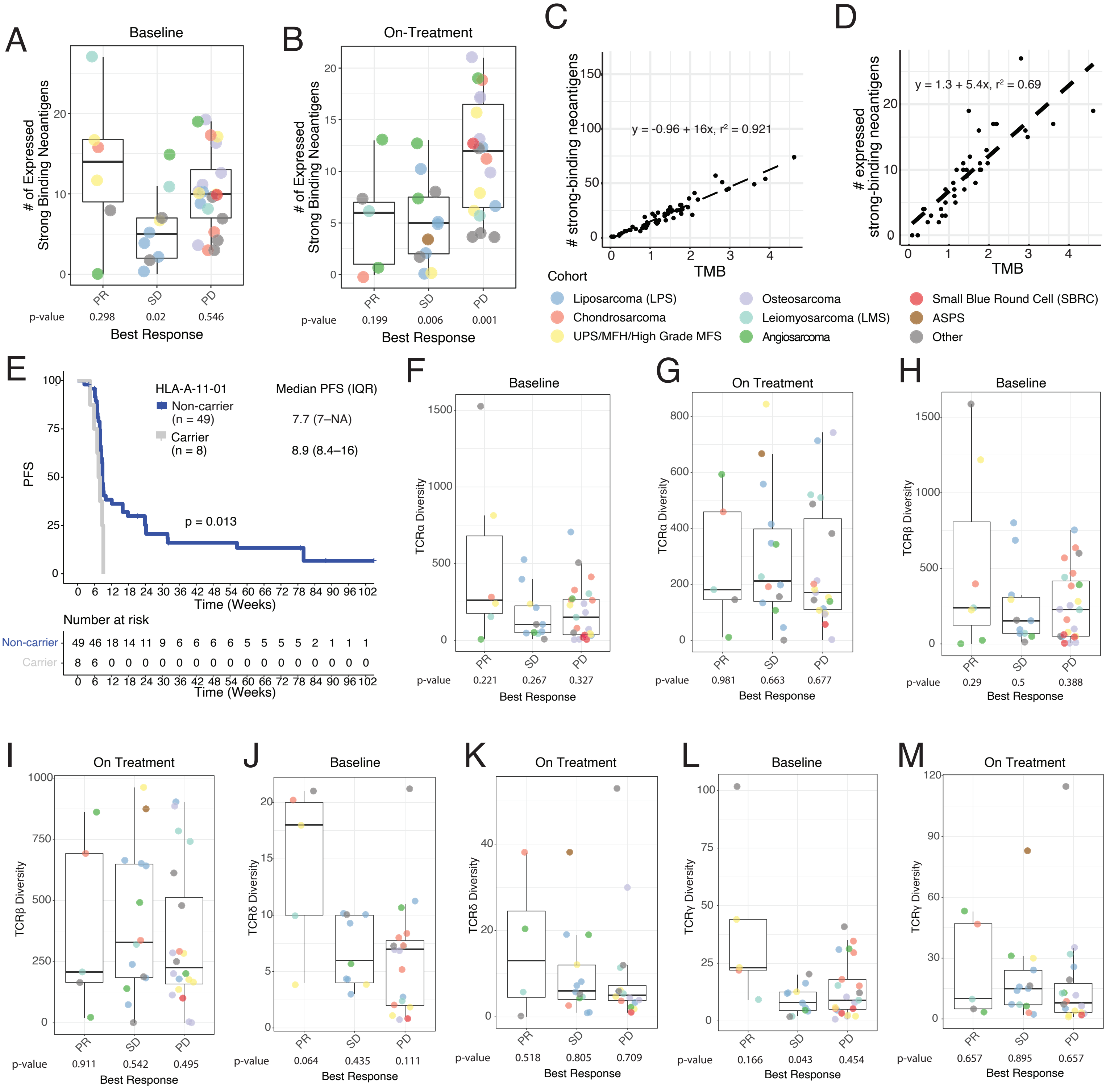

Supplementary Figure 6. Neoantigen expression and HLA genotype differences between responders and non-responders. A–B. Number of expressed strong-binding neoantigens at (**A**) baseline (n = 6 samples [PR], 9 [SD], 21 [PD]) and (**B**) 3 weeks on-treatment (n = 5 samples [PR], 11 [SD], 19 [PD]). Nominal p-values are shown as derived from two-sided t-test vs. all other response groups. **C–D.** Correlation between TMB and (**C**) predicted strong binding neoantigens or (**D**) expressed strong binding neoantigens at baseline. **E.** Kaplan-Meier plot showing the effect of HLA-A-11-01 on progression-free survival. **F-M.** Diversity of T cell receptors α (**F–G**) (Baseline n = 6 samples [PR], 11 [SD], 23 [PD]; On-Treatment n = 5 samples [PR], 14 [SD], 19 [PD]), β (**H–I**) (Baseline n = 7 samples [PR], 11 [SD], 23 [PD]; On-Treatment n = 5 samples [PR], 14 [SD], 20 [PD]), δ (**J–K**) (Baseline n = 5 samples [PR], 9 [SD], 18 [PD]; On-Treatment n = 4 samples [PR], 13 [SD], 16 [PD]) and γ (**L–M**) (Baseline n = 5 samples [PR], 11 [SD], 21 [PD]; On-Treatment n = 5 samples [PR], 13 [SD], 18 [PD]). In (**A-B**) and (**F-M**), patients with PD by non-target lesions are not included. Boxplots in (**A-B**) and (**F-M**) show the median with hinges at 25th and 75th percentile with whiskers extending to smallest or largest value, no more that 1.5 times interquartile range from the hinges. All values are shown with points. p-values calculated by two-sided t-test vs other response categories. Source data are provided as a Source Data file.

MSK PROTOCOL COVER SHEET

A PILOT STUDY OF NKTR-214 AND NIVOLUMAB IN SELECTED PATIENTS WITH LOCALLY ADVANCED/METASTATIC SARCOMA (CA209-9EM)

Sandra D'Angelo, MD/Medicine:

Memorial Sloan Kettering Cancer Center
1275 York Avenue
New York, New York 10065

Table of Contents

1.0 PROTOCOL SUMMARY AND/OR SCHEMA ... 3
2.0 OBJECTIVES AND SCIENTIFIC AIMS ... 5
Primary Objectives ... 5
Secondary Objectives ... 5
3.0 BACKGROUND AND RATIONALE ... 6
Rationale for the Combination of NKTR-214 and Immune Checkpoint Inhibitors ... 9
Clinical Experience with IL-2 and Checkpoint Inhibitors ... 10
Nonclinical ... 10
Clinical Experience with NKTR-214 ... 12
3.3 Nivolumab ... 14
Clinical Experience with Nivolumab ... 14
Flat Dose Regimens with Nivolumab ... 14
Additional details are provided in the nivolumab Investigator Brochure. ... 16
Nivolumab Shorter Infusion Duration ... 16
Nivolumab Safety Summary ... 17
4.0 OVERVIEW OF STUDY DESIGN/INTERVENTION ... 20
4.1 Design ... 20
4.2 Intervention ... 22
5.0 THERAPEUTIC/DIAGNOSTIC AGENTS ... 22
Storage and Stability ... 24
Preparation ... 24
6.0 CRITERIA FOR SUBJECT ELIGIBILITY ... 24
6.1 Subject Inclusion Criteria ... 24
6.2 Subject Exclusion Criteria ... 26
7.0 RECRUITMENT PLAN ... 28
8.0 PRETREATMENT EVALUATION ... 29
9.0 TREATMENT/INTERVENTION PLAN ... 30
10.0 EVALUATION DURING TREATMENT/INTERVENTION ... 36
11.0 TOXICITIES/SIDE EFFECTS ... 49

11.2 Dose Delay and Reduction Criteria 50
Nivolumab Dose Delay Criteria 51
NKTR-214 Dose Delay and Reduction Criteria 52
11.3 Criteria to Resume NKTR-214 or Nivolumab 52
11.4 Permanent Treatment Discontinuation Criteria 53
11.5 Management Algorithms for Immuno-Oncology Agents 55
11. 6 Treatment of NKTR-214 or Nivolumab-Related Infusion Reactions 55
11.7 Prior and Concomitant Medications 58
11.8 Permitted Medications 58
Prohibited Medications 59
12.0 CRITERIA FOR THERAPEUTIC RESPONSE/OUTCOME ASSESSMENT 60
13.0 CRITERIA FOR REMOVAL FROM STUDY 69
14.0 BIOSTATISTICS 71
15.0 RESEARCH PARTICIPANT REGISTRATION AND RANDOMIZATION PROCEDURES 74
15.1 Research Participant Registration 74
16.0 DATA MANAGEMENT ISSUES 75
16.1 Quality Assurance 75
16.2 Data and Safety Monitoring 75
17.0 PROTECTION OF HUMAN SUBJECTS 77
17.1 Privacy 77
17.2 Serious Adverse Event (SAE) Reporting 77
17.2.1 Special SAE Reporting 79
Specific Liver Function Abnormalities 79
17.2.2 SAE Reporting Procedures for BMS (MSK ONLY) 79
17.2.2 SAE Reporting Procedures for Nektar (MSK ONLY) 80
18.0 INFORMED CONSENT PROCEDURES 82
19.0 REFERENCES 82
20.0 APPENDICES 84

1.0 PROTOCOL SUMMARY AND/OR SCHEMA

Study Title:
A Pilot Study of NKTR-214 and Nivolumab in Selected Patients with Locally Advanced/Metastatic Sarcoma (CA209-9EM)

Study Objectives:
Sarcomas comprise a very diverse group of malignancies including more than 50 subtypes of bone and soft tissue origin. Despite optimal approach, approximately 30-80% of patients will develop recurrent and metastatic disease after receiving initial therapy with curative intent. In this setting, responses to standard front-line cytotoxic chemotherapy occur in 10-30% of the cases and median survival is 10-18 months. Sarcomas are immunogenic neoplasms with a need for more therapeutic options. Prior immunotherapeutic agents have shown promise in select sarcoma patients.

Using a combination of NKTR-214, a pegylated IL-2 administered concurrently with the anti-PD1 monoclonal antibody nivolumab to treat patients with selected bone and soft tissue sarcomas and our planned scientific correlates, we believe we can assess the safety and efficacy of immune activating therapy and better understand the effects of combined therapy in antitumoral immune response.

Study Design:
This is an open-label, multi-center, pilot study to evaluate the efficacy of NKTR-214 in combination with nivolumab in patients with selected locally advanced/metastatic, high grade sarcoma **(Figure 1. Study Schema).**

Patient Population:
There will be 9 different cohorts including osteosarcoma, chondrosarcoma, undifferentiated pleomorphic sarcoma/malignant fibrous histiocytoma/high grade myxofibrosarcoma, dedifferentiated/pleomorphic liposarcoma, vascular, leiomyosarcoma, alveolar soft part sarcoma (ASPS), small blue round cell/synovial, and other. A total of 84 evaluable subjects will participate in this study. A total of 88 participants were enrolled due to several inevaluable participants. Participants will include all patients 12 and older.

Treatment Plan:
One treatment cycle will consist of 21 days. Patients will start both study drugs on day one of the first cycle. For both adults (ages ≥ 18) and patients age 12-17, treatment will include nivolumab 360 mg (flat dose) IV and NKTR-214 0.006 mg/kg IV on day 1 and every 3 weeks thereafter.

Patients will be reassessed with imaging studies at week 8 and every 8 weeks thereafter until week 56, and then every 12 weeks thereafter or as per the discretion

of the treating investigator. Treatment will be repeated until the patient develops progressive disease or unacceptable toxicity.

Figure 1: Study Schema

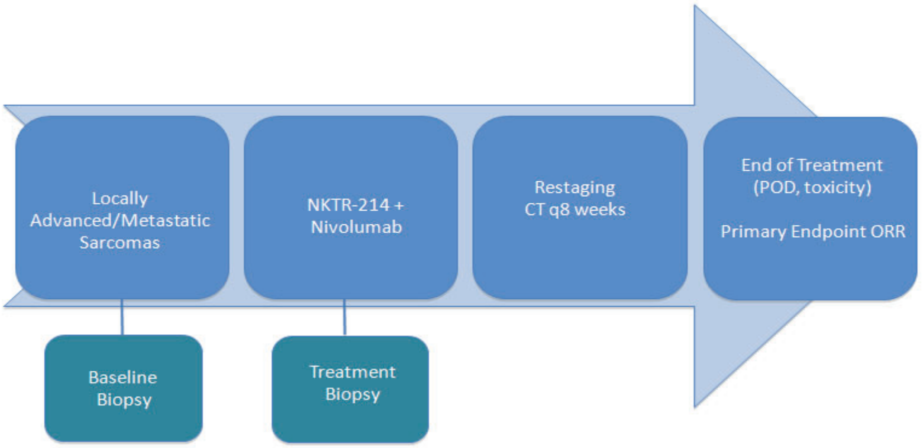

2.0 OBJECTIVES AND SCIENTIFIC AIMS

Primary Objectives

The primary objectives are:

- To evaluate the efficacy of NKTR-214 in combination with nivolumab in patients with selected advanced bone or soft tissue sarcoma, as assessed by the best ORR according to RECIST 1.1 by 24 weeks.

Secondary Objectives

The secondary objectives are:

- To assess the safety and tolerability of NKTR-214 in combination with nivolumab in patients with select advanced bone or soft tissue sarcoma.
- To evaluate the efficacy NKTR-214 in combination with nivolumab in patients with select advanced bone or soft tissue sarcoma, as assessed by ORR according to Immune-related Response Evaluation Criteria in Solid Tumors (irRECIST.)
- To determine the progression-free survival (PFS) at 24 weeks and median PFS according to RECIST 1.1 and to determine overall survival (OS) rate at 12 months and median OS for patients treated with NKTR-214 in combination with nivolumab.

Correlative Objectives

- To determine the baseline characteristics of sarcoma tumors (pre-treatment biopsy sample) evaluated in this study including the level PD-1/PD-L1 expression, presence of tumor infiltrating lymphocytes (TILs) and tumor antigens, gene expression profile, and the T-cell receptor clonality in tumor-infiltrating lymphocytes (TIL).

- To assess the potential effect of NKTR-214 and nivolumab on selected biomarker expression measured in post-treatment tumor tissue and the association between these biomarkers (baseline level of expression and the change in biomarker level of expression following treatment) and clinical outcome, including characterization of PD-1/PD-L1 expression, tumor infiltrating lymphocytes (TILs) and tumor antigens, gene expression profiling, and characterization of T-cell receptor clonality in tumor-infiltrating lymphocytes (TIL).
- To evaluate associations between selected biomarkers measured in serial peripheral blood and with clinical efficacy, including immunophenotyping and functional analyses, evaluation of serum levels of chemokines, cytokines and other immune mediators, and characterization of T-cell receptor clonality in peripheral blood.
- To evaluate the association between baseline tumor mutational burden and neoantigen production with clinical efficacy of the study therapy.

3.0 BACKGROUND AND RATIONALE

3.1 Sarcoma

Sarcomas: New therapies are desperately needed

Sarcomas represent a collection of rare, heterogeneous tumors of mesenchymal origin. There are more than 50 distinct sarcoma subtypes. Approximately 13,000 patients are diagnosed with bone and soft tissue sarcomas every year in the United States. Surgery is the cornerstone of treatment for patients with localized, resectable sarcomas. Adjuvant radiation and chemotherapy are considered in very particular situations. However, despite combined modailty treatment 30-80% of patients develop a recurrence and/or metastatic disease.[1, 2] The median survival for patients with metastatic sarcoma is 10-18 months.[3, 4] Frontline standard cytotoxic chemotherapy agents such as doxorubicin, ifosfamide, and dacarbazine result in objective responses in 10-30% of the patients, and this is significantly influenced by histologic variant.[5] The development of novel and effective therapies is desperately needed for patients with advanced/metastatic sarcomas.

Sarcoma: an immunogenic tumor

Immunotherapeutic strategies may be a promising approach to treating this disease. The role of the immune system as a mechanism of cancer therapy was first observed in a sarcoma patient who had a response after an erysipelas infection.[6] Sarcomas are more common in patients who are immunosuppressed.[7] Infiltration of lymphocytes has been demonstrated in sarcomas.[8] Furthermore, tumor-infiltrating lymphocytes have been associated with improved survival in Ewing's sarcoma and GIST.[9-11] Spontaneous regression of primary tumors have been seen in desmoid tumors and osteosarcomas.[12, 13] There have been multiple clinical trials evaluating the role of immune stimulants such as IL-2, interferon, and liposomal-muramyl tripeptide phosphatidylethanolamine showing some benefit in sarcoma patients.[14-18] T cells genetically engineered to target NY-ESO-1 expressing synovial sarcoma have shown some promise.[19] In patients with GIST, tyrosine kinase inhibitors such as imatinib can have stimulating effects on multiple immune cells.[11]

Immunotherapy in Sarcoma

The results of two clinical trials evaluating the efficacy of single agent PD-1 blockade in the setting of advanced sarcoma were recently presented in 2016 at the annual meeting of the American Society of Clinical Oncology. The first trial, a phase II study, investigated the role of immunotherapy in patients with advanced soft tissue and bone sarcomas. A signal of efficacy for pembrolizumab monotherapy was observed in patients with undifferentiated pleomorphic sarcoma, liposarcoma, osteosarcoma and chondrosarcoma. However, no efficacy was observed in other subtypes such as leiomyosarcoma, synovial sarcoma and Ewing's sarcoma.[20] Nivolumab monotherapy was also deemed to be ineffective in the setting of patients advanced uterine leiomyosarcoma.[21] Immunotherapy research efforts in sarcoma are now focusing on combination immunotherapy strategies.

3.2 NKTR-214

In addition to immune activation using checkpoint inhibitor antibodies, direct immune stimulation using cytokines can also drive immune-mediated cancer cures.[22, 23] Aldesleukin (recombinant human IL-2) directly stimulates the immune system and has been shown to lead to durable responses in ~10% of people with metastatic melanoma and renal cancer.[24] However, in addition to aldesleukin acting as a stimulator of the immune system by activating tumor killing CD8+ T cells (CD8T), it also suppresses the immune system by activating regulatory T (Treg) cells.[25, 26] Despite favorable clinical outcomes associated with aldesleukin, it has several therapeutic limitations including the need for inpatient hospital administration, 5 consecutive days of dosing, and the potential for serious toxicities, comprising capillary leak syndrome, hypotension, and pulmonary edema requiring medical management in the intensive care unit.

A novel cytokine with enhanced immune system activation and the targeted profile of NKTR-214 (i.e., a superior safety profile allowing for outpatient administration and a longer duration of action requiring less frequent dosing) would potentially be an important advancement for the treatment of patients with cancer. NKTR-214 consists of IL-2, which has the same amino acid sequence as aldesleukin, conjugated at a defined region within the protein to releasable polyethylene glycol (PEG) chains. The PEG chains render the molecule inactive. After administration in vivo, the PEG chains are slowly hydrolyzed to generate active cytokine conjugates. The most active IL-2 conjugates are the 2-PEG-IL2 and 1-PEG-IL2. Presumably, the location of the PEG chains on the active conjugated IL-2 reduces the affinity to the IL-2 receptor alpha subunit (IL2Rα), responsible for activating the undesirable Treg cells to a greater extent than the affinity to the IL-2-receptor beta subunit (IL2Rβ) relative to aldesleukin. In the tumor, NKTR-214 preferentially activates CD8T over Tregs. In addition, NKTR-214 provides sustained exposure to active 1-PEG and 2-PEG-IL2 in tumor.

The safety of NKTR-214 was evaluated in rats and monkeys. The MTD was determined to be 0.3 mg/kg in the rat and 0.1 mg/kg in the monkey (see NKTR-214 Investigators Brochure for additional information). Although no combination toxicology studies were conducted with NKTR-214 and nivolumab, no concerns were noted when anti-PD1 antibodies were combined with NKTR-214 in pharmacology studies.

Pre-clinical data for osteosarcoma

A K7M2 syngeneic mouse model of osteosarcoma lung metastasis was used to evaluate the efficacy of NKTR-214. Balb/c mice were given K7M2 via IV route. NKTR-214 was administered at 0.8 mg/kg dosed IV on Days 1, 10, 19 and monitored for survival. The overall survival at day 42 was 82% for NKTR-214 vs 18% for the vehicle control. (Figure 2) NKTR-214 showed clear efficacy as single agent in this disseminated model of metastatic osteosarcoma. In addition, NKTR-214 increased effector cells in metastasized lung tumors.

Figure 2: NKTR-214 in Osteosarcoma

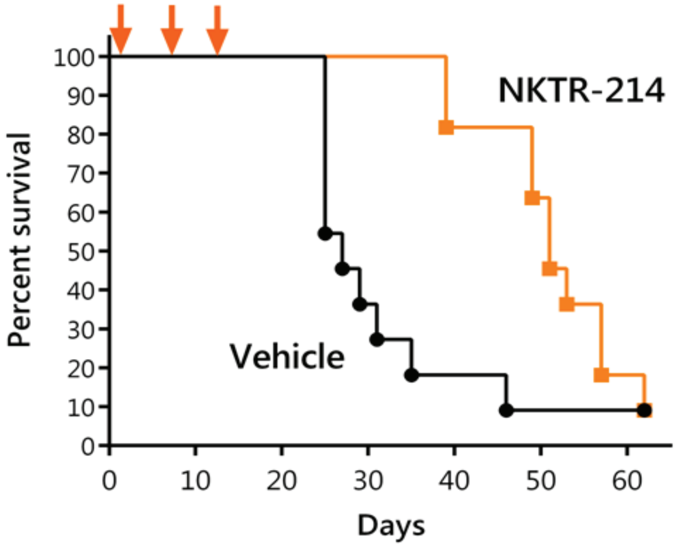

Figure 2: NKTR-214 provides survival benefit as a single agent in a model of metastatic osteosarcoma.Balb/c mice were inoculated with K7M2 cells via IV route on Day 0 and monitored for survival. Arrows: NKTR-214 0.8 mg/kg (or vehicle) dosed IV on Days 1, 10, 19.

Rationale for the Combination of NKTR-214 and Immune Checkpoint Inhibitors

Accumulating evidence suggests that patients with low baseline CD8+ T cells within the tumor microenvironment (tumor infiltrating lymphocytes [TILs]) predict poor response to checkpoint inhibitor immunotherapies, thus, agents designed to specifically activate and expand CD8+ T cells may improve clinical outcomes in patients with low TILs.[27, 28] NKTR-214 targets the IL-2 pathway and is designed to provide biased sustained signaling through the heterodimeric IL-2 receptor pathway (IL-2Rβγ) to preferentially activate and expand NK and effector CD8+ T cells over Treg cells. Preliminary analyses of patients' blood and tumor by flow cytometry and immunohistochemistry (IHC) demonstrate that NKTR-214, as a single agent, increases activated CD4+ and CD8+ T cells in peripheral blood with an increase in T cell infiltrates within the tumor tissue after 1 dose of NKTR-214. In addition, there is an increase in programmed cell death receptor-1 (PD-1) expression on T cells in the blood and tumor after treatment with NKTR-214. The

ability to alter the immune environment and increase PD-1 expression on effector T cells may improve the effectiveness of anti-PD-1 blockade.

Clinical Experience with IL-2 and Checkpoint Inhibitors

Simultaneous administration of IL-2 with immune checkpoint inhibitors directed against PD-1 (nivolumab and pembrolizumab) or its ligand has not been reported as of the date of this protocol, although several studies are ongoing in which IL-2 and an anti-PD-1 antibody are co-administered following an infusion of either adoptive CD8+ T cells or tumor-infiltrating lymphocytes (NCT02757391, NCT02500576).

Published data on the sequential administration of IL-2 with an anti-PD-1 inhibitor have been reported recently. A case report noted a near-complete response (near-CR) with extended duration of response when a patient with renal cell carcinoma (RCC; non-responsive to nivolumab) was treated with high-dose IL-2.[29] A larger experience from an observational clinical trial reported that in patients with metastatic melanoma who received high-dose IL-2 and received either ipilimumab or an anti-PD-1 inhibitor experienced a differential increase in median overall survival, 15.8 vs 28.7 months, respectively. The 12-month survival rate was 64% for patients receiving ipilimumab post high-dose IL-2 compared with 97% for patients receiving an anti-PD-1 inhibitor, suggesting that these two checkpoint inhibitors do not have overlapping mechanisms of action and that IL-2 may have enhanced the curative potential of the anti-PD-1 inhibitor.[30] In support of the hypothesis that IL-2 may have enhanced the curative potential of nivolumab/pembrolizumab, a recent report demonstrated that IL-12, which has been shown to induce tumor infiltrating lymphocytes (TILs) and anti-tumor immunity similar to IL-2, appeared to prime response to anti-PD-1/PD-L1 inhibitors and demonstrated a clinical benefit rate of 75% (50% CR and 25% partial response [PR]).[31]

Nonclinical

Nonclinical studies have highlighted the important role of IL-2 in combination regimens, especially with immune checkpoint inhibitors. Using a nonclinical murine model, showed that combinations of CTLA-4 with PD-1/PD-L1 or IDO blockade was superior to single-agent therapy and that this was due to restoration of IL-2 production and proliferation of CD8+ T cells within the tumor microenvironment.[32] The combination of low dose IL-2 and PD-L1 blockade was tested in a mouse model of chronic lymphocytic choriomeningitis virus and showed a significant expansion of preexisting exhausted CD8 T cells that was dependent on IL-2.[33] These observations provide a nonclinical rationale for combining IL-2 with checkpoint blockade treatment.

The effects of NKTR-214 in combination with murine anti-CTLA-4, anti-PD-1, or anti-PD-L1 antibody were evaluated in different murine tumor models including lung and

breast (Figure 3, and osteosarcoma (Figure 4). The combination of NKTR-214 with anti-CTLA4, anti-PD-1, or anti-PD-L1 was more effective than either agent alone, resulting in tumor-free animals. In all nonclinical species tested, NKTR-214 resulted in stimulation of several markers of immune system activation, in particular lymphocyte counts and sCD25.

Figure 3: NKTR-214 and Anti-PD-1 Antibody Are Synergistic and Result in Tumor-Free Animals

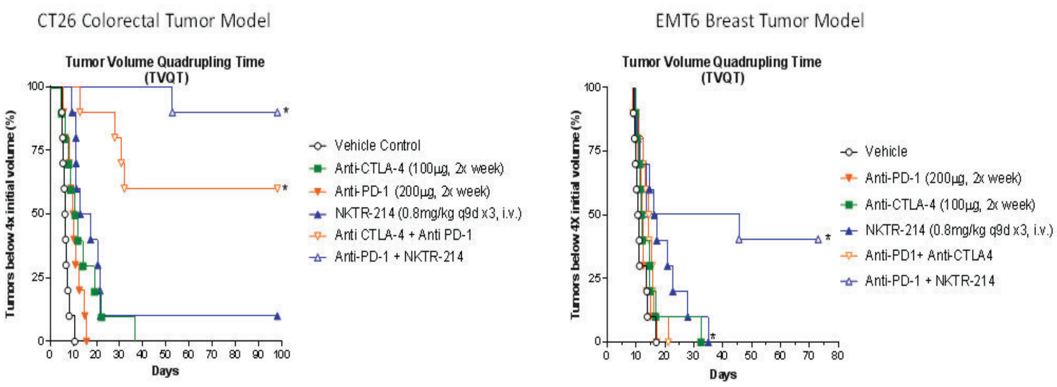

Figure 3: Tumors were allowed to grow in excess of 100 mm^3 before dosing was initiated on Day 0. For combinatorial treatment, anti-PD1 antibody was initiated on Day 0 followed by NKTR-214 initiation on Day 4. Anti-PD-1 (200 µg) was administered i.p., twice weekly. NKTR-214 (0.7 mg/kg) was administered IV every 9 days. Individual Tumor Volume Quadrupling Time (TVQT) was assessed by extracting the second order polynomial quadratic equation for each tumor's growth curve and interpolating the time to achieve 4x initial volume, with graphical representation on a Kaplan-Meier curve. Statistical analysis was performed by the Log-Rank test (* $p < 0.05$ relative to vehicle).

Figure 4: NKTR-214 shows efficacy in combination with checkpoint inhibitor in a solid tumor (LM8) model of osteosarcoma

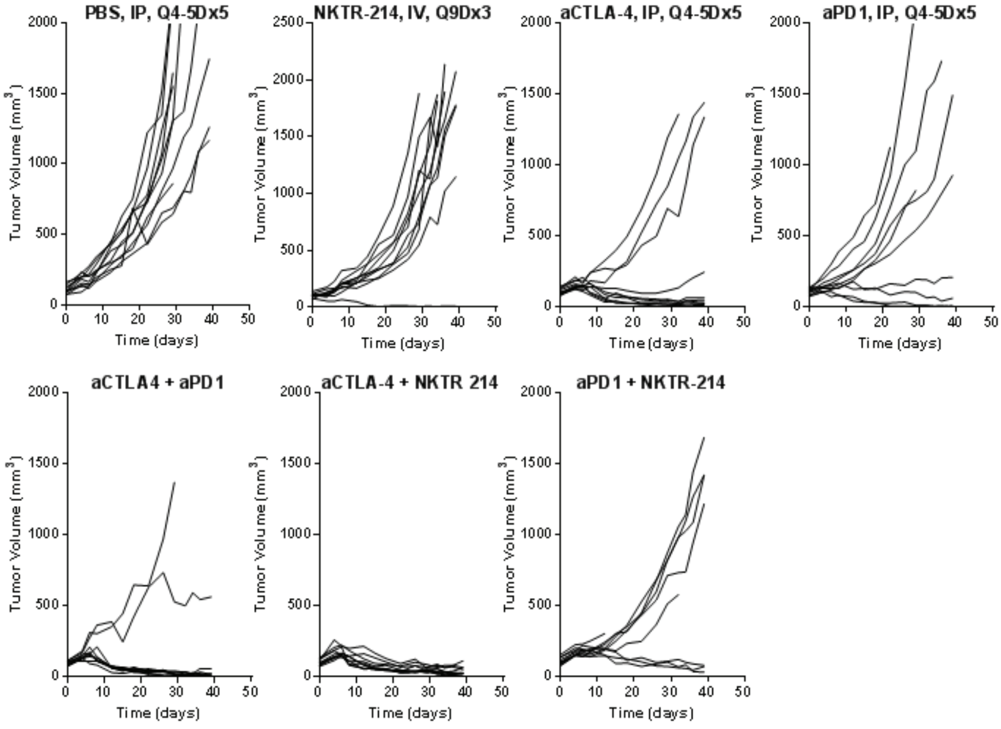

Clinical Experience with NKTR-214

Study 15-214-01 (NCT02869295) is a Phase 1/2 open-label, multicenter, dose escalation and dose expansion monotherapy study of NKTR-214 in patients with locally advanced or metastatic solid tumors. The objectives of the study are to evaluate the safety and tolerability of NKTR-214 to determine the maximum tolerated dose (MTD) as well as to assess the objective response rate at or below the MTD, or to identify the recommended Phase 2 dose (RP2D). The RP2D has determined to be NKTR-214 0.006mg/kg.

In Study 15-214-01, hypotension has been identified as a principal toxicity. Hypotension is a known adverse event (AE) associated with both IL-2 and engineered cytokines. Instances of hypotension most commonly appeared 2-3 days following the first infusion, coinciding with the peak plasma concentration of the active metabolites formed after administration of NKTR-214. Patients for whom

intravenous (IV) fluid administration was clinically indicated responded rapidly (in less than 24 hours) to IV hydration. To mitigate the risk of hypotension, management guidelines were implemented and included the following recommendations:

- Discontinuation of antihypertensive therapy and drugs with hypotensive properties, prior to administration of NKTR-214.
- Maintaining adequate oral fluid intake, particularly during the first 5 days post-dose.
- Avoiding activities that could lead to dehydration (e.g., physically strenuous activity) or vasodilation (e.g., hot showers, sauna). Providing additional corticosteroid support for patients with adrenal insufficiency on corticosteroid replacement therapy.
- Since the implementation of these mitigation measures, the frequency of hypotension, particularly Grade 3 hypotension, has been reduced.

As of 26 May 2017, 28 patients were dosed and completed at least one cycle in the dose escalation phase of ongoing monotherapy Study 15-214-01. In Part 1, dose escalation, patients are enrolled in groups of at least 3 patients per cohort with doses administered as an IV infusion over 15 (± 5) minutes every 21 days (q21d). No dose-limiting toxicities (DLTs) were observed at 0.003, 0.006, or 0.009 mg/kg. One patient dosed a 0.012 mg/kg experienced cytokine release syndrome and the DLTs of syncope and hypotension; this patient received another 2 cycles of NKTR-214 at a lower dose of 0.006 mg/kg and tolerated treatment well.

In contrast to aldesleukin, NKTR-214 does not appear to produce capillary leak syndrome; the severity and duration of any temporally associated hypotension is markedly reduced. The pattern of AEs attributable to NKTR-214 observed in Study 15-214-01 is dissimilar from the immune-mediated AEs (imAEs) that commonly develop with the use of checkpoint inhibitors. Although limited clinical data currently exist with NKTR-214, neither clinical nor nonclinical data predict “immune-mediated inflammatory events,” such as hypophysitis, colitis, nephritis, and hepatitis to occur with NKTR-214. The non-overlapping toxicities observed with NKTR-214 and nivolumab may therefore result in a reasonably well-tolerated immuno-oncology combination.’

Additional details on the safety profile of NKTR-214 are provided in the NKTR-214 Investigator’s Brochure.

3.3 Nivolumab

Clinical Experience with Nivolumab

Nivolumab is indicated for the treatment of patients with metastatic NSCLC with progression on or after platinum-based chemotherapy. Nivolumab added to chemotherapy has been evaluated in several cohorts of chemotherapy-naive patients with advanced NSCLC at a frequency of every 3 weeks (q3w). Nivolumab 10 mg/kg was combined with gemcitabine + cisplatin and pemetrexed + cisplatin. Nivolumab 10 mg/kg and nivolumab 5 mg/kg were combined with paclitaxel and carboplatin. The frequency of most immune-related select AEs was higher than what has been observed for nivolumab monotherapy. However, these treatment-related AEs, including pneumonitis, were effectively managed and did not lead to any deaths.

Population PK (PPK) analyses have shown that the PK of nivolumab is linear with proportional exposure over a dose range of 0.1 to 10 mg/kg, and no differences in PK across ethnicities and tumor types were observed. As the PK of nivolumab is linear, the corresponding flat dose for a q3w dosing regimen is nivolumab 360 mg. Using the PPK model, the exposures following administration of several dosing regimens of nivolumab administered as a flat dose were simulated, including 360 mg administered q3w. The simulated steady-state average concentration (C_{avgss}) following administration of nivolumab 360 mg q3w are expected to be similar to those following administration of nivolumab 3 mg/kg q2w to patients weighing 80 kg, the approximate median weight of patients used in the PPK analyses. The predicted steady-state peak (C_{maxss}) concentrations following nivolumab 360 mg q3w are predicted to be less than those following the administration of nivolumab 10 mg/kg q2w, providing sufficient safety margins. Currently, nivolumab is being studied as nivolumab 360 mg combined with platinum-doublet chemotherapy administered q3w for the treatment of NSCLC in a Phase 3 study.

Long infusion times place a burden on patients and treatment centers. In a Phase 2, randomized, double blinded, dose-ranging study of nivolumab in patients with advanced/metastatic clear cell RCC, a dose association was observed for infusion site reactions and hypersensitivity reactions (1.7% at 0.3 mg/kg, 3.7% at 2 mg/kg, and 18.5% at 10 mg/kg). All the events were Grade 1-2 and were manageable. An infusion duration of 30 minutes for 360 mg flat dose of nivolumab (~ 45% of the dose provided at 10 mg/kg) are not expected to present new safety concerns compared with 10 mg/kg nivolumab dose infused over 60 minutes.

Flat Dose Regimens with Nivolumab

The safety and efficacy of 240 mg every 2 weeks (q2w) flat dose of nivolumab is expected to be similar to the 3 mg/kg q2w dosing regimen. A flat dose of nivolumab

of 240 mg q2w is identical to a dose of 3 mg/kg for patients weighing 80 kg, the observed median body weight in nivolumab-treated cancer patients. Using a population pharmacokinetics (PPK) model, the overall distribution of nivolumab exposures are comparable after treatment with either 3 mg/kg or 240 mg nivolumab. The predicted range of nivolumab exposures (median and 90% prediction intervals) resulting from a 240 mg flat dose across the 35 to 160 kg weight range is maintained well below the corresponding exposures observed with the well-tolerated 10 mg/kg nivolumab q2w dose. Across the various tumor types in the nivolumab clinical program, nivolumab has been shown to be safe and well tolerated up to a dose of 10 mg/kg, and the relationship between nivolumab exposure produced by 3 mg/kg and efficacy and safety is relatively flat. Given the similarity of nivolumab pharmacokinetics (PK) across tumor types and the similar exposures predicted following administration of a 240 mg flat dose compared with 3 mg/kg q2w, the safety and efficacy of 240 mg q2w is expected to be similar to 3 mg/kg q2w. Hence, a flat dose of 240 mg nivolumab is under investigation. Further, the flat dosing was recently FDA approved for the treatment of metastatic melanoma which was previously 3mg/kg.

Nivolumab 480 mg administered every 4 weeks (q4w) is currently under investigation. The less frequent dosing schedule is designed to be more convenient for patients. The 480 mg dose was chosen based on clinical data as well as modeling and simulation approaches using PPK and exposure-response analyses of data from studies in multiple tumor types (melanoma, non-small cell lung cancer [NSCLC], and RCC) to provide an approximately equivalent dose as 3 mg/kg q2w. Exposures with the 480 mg q4w regimen are predicted to be within the exposure ranges observed at doses up to 10 mg/kg q2w assessed in the nivolumab clinical program and, therefore, are not considered to cause an increased risk to patients.Additional details are provided in the nivolumab Investigator’s Brochure.

Nivolumab Clinical Experience in Pediatric Patients

Nivolumab administration in pediatric patients with relapsed/refractory solid tumors or sarcomas is currently under investigation in the phase I/II study ADVL1412 (NCT02304458). As of Decemeber 2018, 83 patients were dosed and completed at least one cycle. Patients received nivolumab infusions at the adult RP2D, 3 mg/kg q2w which is as previously noted is equivalent to the flat dose of 240 q2w. No dose-limiting toxicities (DLTs) were observed at 3 mg/kg. Pediatric patients will received a flat dose regiment based on FDA guidance. Pharmacokinetic data demonstrated age and body weight had no clinically meaningful effect on the steady state exposure of nivolumab and that the drug exposure is generally similar between adults and

pediatric patients age 12 years and older for monoclonal antibodies. A flat dose regimen based on a population PK model that was developed by pooling adult data with CA209-070 pediatric patients receiving nivolumab monotherapy (N=1887). The exposure for the pediatric subjects with solid tumor (N=27, Age ranging from 2-17 yrs) was predicted for 240 mg Q2W vs 3 mg/kg Q2W.

For patients with body weight >=40 kg (N=17), the exposure generally increased ~33% with 240 mg Q2W treatment vs 3 mg/kg Q2W. Such increased exposure is still well below the safety margin we have established from the 10 mg/kg Q2W. To be specific, the geometric means of Cmin, Cmax and Cavg at steady state are predicted to be 95.7, 185 and 122 ug/mL, respectively, with nivolumab i.v. 240 mg Q2W treatment, compared to the geometric means of Cmin, Cmax and Cavg at steady state 71.7, 139 and 91.3, respectively, with nivolumab i.v. 3 mg/kg Q2W treatment. For patients with body weight < 40 kg (N=11), the exposure will be increased >350% and is still within the upper boundary of safety margin established by 10 mg/kg Q2W.

Pediatric pharmacokinetics of single agent nivolumab 3 mg/kg are shown to be similar to adults. Population PK (PPK) analyses have shown that the PK of nivolumab is linear with proportional exposure over a dose range of 0.1 to 10 mg/kg, and no differences in PK across ethnicities or tumor types were observed. As the PK of nivolumab is linear, the corresponding pediatric dose for q3w is the flat dose of 360mg..

Additional details are provided in the nivolumab Investigator Brochure.

Nivolumab Shorter Infusion Duration

Establishing that nivolumab can be safety administered using a shorter infusion time (30 minutes) is under investigation. Previous clinical studies of nivolumab monotherapy have used a 60-minute infusion duration, and nivolumab has been safely administered up to 10 mg/kg over long treatment periods. Infusion reactions including high-grade hypersensitivity reactions have been uncommon across the nivolumab clinical program. In study CA209010, a dose association was observed for infusion site reactions and hypersensitivity reactions (1.7% at 0.3 mg/kg, 3.7% at 2 mg/kg, and 18.5% at 10 mg/kg). All of the events were Grade 1-2 and were manageable. An infusion duration of 30 minutes for 3 mg/kg nivolumab (30% of the 10 mg/kg dose) is not expected to present any safety concerns based on the prior experience of 10 mg/kg infused over 60 minutes. The safety of 3 mg/kg nivolumab administered as a 30-minute infusion was assessed in CA209153 in patients (n = 322) with previously treated advanced NSCLC. Overall, there were no clinically meaningful differences in the frequency of hypersensitivity/infusion-related reactions

(of any cause or treatment related) between patients infused over 30 minutes versus the frequency reported for 60-minute infusions. Thus, nivolumab is consider safe to infuse over 30 minutes.

Additional details are provided in the nivolumab Investigator's Brochure.

Clinical Experience with nivolumab and NKTR-214.
Study 16-214-02 (NCT02983045) is A Dose Escalation and Cohort Expansion Study of NKTR-214 in Combination With Anti-PD-1 Antibody (Nivolumab) in Patients With Select Advanced or Metastatic Solid Tumors (PIVOT-02.) This is an on-going phase I/II study. As of 01 June 2017, 20 patients were dosed and completed at least one dosing cycle. There have been no dose limiting toxicities or grade 3 SAE demonstrated with NKTR 0.006mg/kg q3weeks and nivolumab 240 mg flat dose. With this dose level, there has been 1 patient that had evidence of a complete response. Adverse events of grade 1 included diarrhea, nausea, vomiting, chills, fatigue, fever, decreased appetite, back pain, myalgias, akathisia, headache, pruritus, rash, flushing. Adverse events of grade 2 included ascites, upper respiratory infection and rash.

In addition, safety has also been demonstrated with nivolumab 240mg q2 weeks and NKTR 0.006mg/kg q2weeks. There have been no grade 3 or higher treatment related adverse events. This likely will be the R2PD. At this time, there are on-going plans to treat additional patients at this dose level and evaluate the pK samples to finalize the dose.

Nivolumab Safety Summary
Overall, the safety profile of nivolumab monotherapy as well as combination therapy is manageable and generally consistent across completed and ongoing clinical trials with no MTD reached at any dose tested up to 10 mg/kg. There was no pattern in the incidence, severity, or causality of AEs to nivolumab dose level. Most AEs were low-grade (Grade 1 to 2) with relatively few related high-grade (Grade 3 to 4) AEs. Most high-grade events were manageable with the use of corticosteroids or hormone replacement therapy (endocrinopathies) as instructed in the Management Algorithms **(Appendix 3).**

A total of 39 and 306 patients with selected recurrent or treatment-refractory malignancies have been treated in a completed Phase 1 single-dose study (CA209001) and an ongoing Phase 1 multidose study (CA209003), respectively. Because the safety profile from CA209003 to date is consistent with that observed for CA209001, only data from the larger and more recent study, CA209003, is presented below.

In CA209003 (n=306, including 129 patients with NSCLC), as of the 05-Mar-2013 data base lock, drug related AEs of any grade occurred in 75% of patients. The most frequent drug-related AEs occurring in > 5% of patients included fatigue (28%), rash (15%), diarrhea (13%), pruritus (11%), nausea (9%), decreased appetite (9%), hemoglobin decreased (6%) and pyrexia (6%). The majority of events were low grade, with grade 3/4 drug-related AEs observed in 17% of patients. The most common Grade 3/4 drug-related AEs occurring in > 1% of patients were fatigue (2%), pneumonitis (1%), diarrhea (1%), abdominal pain (1%), hypophosphatemia (1%), and lymphopenia (1%). Drug-related serious AEs (SAEs) occurred in 14% of patients; 8% were of Grade 3/4 including pneumonitis (1%) and diarrhea (1%). The spectrum, frequency, and severity of drug-related AEs were generally similar across the dose levels tested. A review of the safety data by tumor type (RCC, NSCLC, mCRPC, CRC, and melanoma) also did not show any clinically meaningful differences in the proportion of patients with AEs noted across tumor type.

Select adverse events with potential immune-related causality, previously termed “immune-related adverse events” or “adverse events of special interest” were also analyzed taking into account multiple events, with rates adjusted for treatment duration. Most events occurred within the first 6 months of therapy; cumulative or novel toxicities were not observed with prolonged drug exposure. Nineteen of 306 patients (6%) experienced grade 3-4 treatment-related select adverse events. Fifty-two of 230 patients (23%) with drug-related adverse events required management with systemic glucocorticoids and/or other immunosuppressive agents. Twenty-one of 52 (40%) resumed nivolumab therapy after toxicity resolved, while others discontinued therapy.

Although tumor progression was the most common cause of mortality, there were 3 drug-related deaths associated with grade 3-4 pneumonitis. Pneumonitis (any grade) occurred in 12 of 306 patients (4%), and grade 3-4 pneumonitis occurred in 4 patients (1%), with clinical presentations ranging from asymptomatic radiographic abnormalities to progressive, diffuse pulmonary infiltrates associated with cough, fever, and/or dyspnea. No clear relationship between the occurrence of pneumonitis and tumor type, dose level, or treatment duration was noted. In 9 of 12 patients, pneumonitis was reversible with treatment discontinuation and/or immunosuppression (glucocorticoids, infliximab, mycophenolate). There were no pneumonitis-associated deaths between November 2011 and March 2013, the point of data analysis, 79 patients continued to receive nivolumab during this time (median 29 weeks, range 2-69 weeks).

NKTR-214 was designed to mitigate the serious toxicities associated with rapid systemic immune activation seen with administration of aldesleukin. The goal of engineering a PEGylated form of IL-2 that reduces the treatment-limiting toxicities

of aldesleukin, necessitating in-hospital administration, appears to have been successfully realized with NKTR-214 at the doses tested.

The safety profile of nivolumab is well characterized and manageable, including combination with other immuno-oncology products. Nonclinical data as well as clinical experience with high-dose IL-2 and checkpoint inhibitor combinations indicate the potential for improvement in therapeutic response. Thus, the potential benefit of combination therapy appears to outweigh the known risks of these agents and warrants clinical investigation.

Rationale for Expansion Dose

There have been 28 patients treated with NKRT-214 single agent, and has been well tolerated with a favorable safety profile. 22 patients have been treated at q3w, 4@0.003, 11@0.006, 6@0.009 and 1@0.012 mg/kg. Six patients received 0.006 mg/kg q2w. The most common Grade 1-2 TRAEs were fatigue (73%) and pruritus (64%), and decreased appetite (46%). One patient experienced Gr3 syncope and hypotension at the highest dose tested and continued treatment at a lower dose. Immune-related AEs and capillary leak syndrome were not observed. NKTR-214-related hypotension was predictable, occurring within 3-4 days post NKTR-214, short-lived and managed with oral or IV fluids. Mitigation measures have been instituted which have substantially reduced the frequency and severity of hypotensive episodes further facilitating the outpatient administration of NKTR-214. Based on these data, both q2w and q3w dosing schedules, at 0.003 and0.006 mg/kg, of NKTR-214 have been deemed safe per Safety Review Committee (see Table).

As of May 11, 2017, 18 patients have been dosed with NKTR-214 and nivolumab in combination. Four patients have been dosed with NKTR-214 0.006 mg/kg q3w combined with nivolumab 240 mg q2w, 6 patients have been dosed with NKTR-214 0.003 or 0.006 mg/kg combined with nivolumab q2w, and 8 patients have been dosed with NKTR-214 0.006 mg/kg and nivolumab q3w (see Table). The Safety Review Committed has performed a formal safety review in 8 patients, and no dose limiting toxicities were observed, no immune-related adverse events or treatment-related Grade 3 or 4 adverse events have been reported.

NKTR-214 Dosing frequency	Nivolumab (*flat dose) Dosing frequency	NKTR-214 dose (mg/kg)	No. of patients dosed	No. patients experiencing a DLT	No. patients examined for safety	Positive biomarker data and/or evidence of clinical activity

Q3w	N/A	0.003, 0.006, 0.009, 0.012	22	0	22	+
Q2w	N/A	0.006	6	0	6	+
Q3w	Q2w	0.006	4	0	4	+
Q2w	Q2w	0.003	3	0	N/A	+
Q2w	Q2w	0.006	3	0	N/A	+
**Q3w**	**Q3w**	**0.006**	8	0	4	+

*nivo q2w = 240 mg, q3w = 360 mg.

Population PK (PPK) analyses have shown that the PK of nivolumab is linear with proportional exposure over a dose range of 0.1 to 10 mg/kg. Hence, exposure of nivolumab 360 mg q3w is equivalent to that of nivolumab 240 mg q2w. Using the PPK model developed, the exposures following administration of several dosing regimens of nivolumab (administered as a flat dose) were simulated, including 360 mg administered q3w. The simulated steady state average concentration (Cavgss) following administration of nivolumab 360 mg q3w are expected to be similar to those following administration of nivolumab 3 mg/kg q2w to subjects weighing 80 kg, the approximate median weight of subjects used in the PPK analyses.

The FDA-approved dosing schedule of anti-PD1 is either q2w (nivolumab) or q3w (pembrolizumab) and appears to not impact clinical outcomes, thus the nivolumab schedule will be determined by the optimal dosing schedule for NKTR-214 based on the observed PK/PD. The biomarker data derived from the evaluation of blood and tumor tissue samples from the NKTR-214 monotherapy and combination studies support similar immune activation with q2w and q3w dosing schedules. Therefore, the recommended dose for the expansion cohorts will be NKTR-214 0.006 mg/kg q3w plus nivolumab 360 mg for both pediatric and adult patients.

4.0 OVERVIEW OF STUDY DESIGN/INTERVENTION

4.1 Design

This is a multi-center, pilot study to evaluate the efficacy of the NKTR-214, given in combination with the anti-PD1 monoclonal antibody, nivolumab, for patients with selected metastatic and/or locally advanced high-grade sarcomas **(Table 1)**.

For participants ≥18 years old NKTR-214 0.006mg/kg will be an intravenous (IV) infusion administered over 30 (±5) minutes every 3 weeks. Nivolumab 360mg flat dose IV infusion administered over 30 (±5) minutes every 3 weeks.

For participants 12 – 17 years old NKTR-214 0.006mg/kg will be an intravenous (IV) infusion administered over 30 (±5) minutes every 3 weeks. Nivolumab 360mg flat dose IV infusion administered over 30 (±5) minutes every 3 weeks.

The treatment period of the study is divided into multiple 21 day cycles every 3 weeks with associated evaluations and procedures.

**Table 1: Sarcoma Subtypes**
Osteosarcoma (n=10)
Chondrosarcoma (n=10)
Undifferentiated pleomorphic sarcoma/malignant fibrous histiosarcoma/ high grade myxofibrosarcoma (n=10)
Dedifferentiated/pleomorphic liposarcoma (n=10)
Leiomyosarcoma (n=10)
Vascular (n=10)
Alveolar Soft Part Sarcoma (n=4)
Small Blue Round Cell/Synovial (n=6)
Other (n=14)

Patients will be evaluated radiographically at baseline, week 8 (+/- 7 days) and every 8 weeks (+/- 7 days) subsequently until week 56 and then every 12 weeks thereafter or as per the discretion of the treating investigator. Best ORR will be assessed using RECIST v1.1 (primary response assessment) and irRECIST (secondary response assessment) by 24 weeks.

All adult study participants will undergo mandatory tumor biopsies at baseline and week 3 (Cycle 2) +/- 1 week. Optional biopsies at progression will be offered. Serial blood samples will be obtained prior to treatment, during treatment, and at the end of study treatment.

Research biopsies are not required for participants ages 12-17. Archival tissue will be collected if available. Research bloods samples will be obtained prior to treatment, during treatment, and at the end of study treatment. Additional, pharmacokinetic samples will be required for pediatric patients.

Study therapy should continue provided that the subject has no evidence of confirmed progression of disease by RECIST 1.1, has no clear clinical deteroriation/progression, no incurrent illness that prevents the administration of

further treatment. Treatment beyond progression will be allowed at the discretion of the clinical investigator as long as patient is clinically benefitting, is tolerating the drug well and continues to meet all study treatment criteria.

Patients with a PR or CR will continue treatment for up to 24 months unless one of the criteria for treatment discontinuation is met. After 24 months, patient's with a PR or CR per RECIST 1.1 criteria can be followed every 12 weeks (+/- 14 days) until confirmed disease progression with the following assessments:

- Standard imaging including CT chest/abdomen/pelvis (with or without contrast) and/or MRI of the affected area if deemed necessary by the study investigator.
- Routine laboratory tests including CBC with differential, comphrensive serum chemistry oanel (including glucose, blood urea nitrogen, creatinine, sodium, potassium, chloride, bicarbonate, calcium, total protein, albumin, serum bilirubin, alkaline phosphate, ALT, and AST) and TSH
- MD visit (physical examination)

If there is evidence of recurrence, treatment can be resumed.

Patients with SD will continue study treatment unless one of the criteria for treatment discontinuation is met.

4.2 Intervention

Patients will start both study drugs on day one of the first cycle and subsequent treatment cycles.

Each patient's NKTR-214 dose will be determined by the the patient's weight in kilograms. The patient's weight will be determined before the start of each cycle and dosing will remain the same unless there's a >10% change in weight.

For adult and pediatric participants, NKTR-214 0.006mg/kg and nivolumab 360mg will be administered intravenously on day 1 of week 1 of cycle one and every 3 weeks (±3 days) thereafter.

NKTR-214 will be administered first before nivolumab.

5.0 THERAPEUTIC/DIAGNOSTIC AGENTS

5.1 NKTR-214

Mechanism of Action of NKTR-214

A novel cytokine with enhanced immune system activation and the targeted profile of NKTR-214 (i.e., a superior safety profile allowing for outpatient administration and a longer duration of action requiring less frequent dosing) would potentially be an important advancement for the treatment of patients with cancer. NKTR-214 consists of IL-2, which has the same amino acid sequence as aldesleukin, conjugated at a defined region within the protein to releasable polyethylene glycol (PEG) chains. The PEG chains render the molecule inactive. After administration in vivo, the PEG chains are slowly hydrolyzed to generate active cytokine conjugates. The most active IL-2 conjugates are the 2-PEG-IL2 and 1-PEG-IL2. Presumably, the location of the PEG chains on the active conjugated IL-2 reduces the affinity to the IL-2 receptor alpha subunit (IL2Rα), responsible for activating the undesirable Treg cells to a greater extent than the affinity to the IL-2-receptor beta subunit (IL2Rβ) relative to aldesleukin. In the tumor, NKTR-214 preferentially activates CD8 T cells over Tregs. In addition, NKTR-214 provides sustained exposure to active 1-PEG and 2-PEG-IL2 in tumor.

NKTR-214 Drug Description. Formulation and Storage

The investigational drug product, NKTR-214, is formulated as a sterile lyophilized powder for reconstitution in single-use glass vials. Lyophilized NKTR-214 drug product will be diluted with commercially available Dextrose 5% in water for injection (D5W). Each 5 mL, clear glass, single-use vial contains 1.1 mg of recombinant human interleukin 2 (rhIL-2) equivalents (including 0.1 mg of overage). NKTR-214 is formulated in 10 mM citrate buffer, 7% (w/v) trehalose, pH 4.0.

The instructions for reconstitution and administration of NKTR-214 drug product are described in the Pharmacy Manual (Appendix 6).

NKTR-214 drug product should be stored in a secure, locked area with temperature at -20°C (± 5°C), as specified on the drug label.

See instructions for reconstitution and administration of NKTR-214 drug product as described in the Pharmacy Manual (**Appendix 6**).

For administration of NKTR-214, please refer to Section 9.1 Study Drugs.

5.2 Nivolumab

Mechanism of Action of Nivolumab

Nivolumab (BMS-936558, MDX-1106, and ONO-4538) is a fully human monoclonal immunoglobulin G4 (IgG4) antibody (HuMAb) that is specific for human programmed

death-1 (PD-1, cluster of differentiation 279 [CD279]) cell surface membrane receptor (Investigator Brochure, 2014). PD-1 is a negative regulatory molecule that is expressed transiently following T-cell activation and on chronically stimulated T cells characterized by an "exhausted" phenotype.

Formulation Storage and Packaging
Nivolumab Injection, 100 mg/10 mL (10 mg/mL), is a clear to opalescent, colorless to pale yellow liquid; light (few) particulates may be present. The drug is a sterile, nonpyrogenic, single-use, isotonic aqueous solution formulated in sodium citrate, sodium chloride, mannitol, diethylenetriamine pentaacetic acid (pentetic acid) and polysorbate 80 (Tween 80®), pH 6.0. Nivolumab injection is supplied as 100 mg vials (10 mg/mL) with a 0.7 mL overfill. It is supplied in 10 mL Type I flint glass vials, with butyl rubber stoppers and aluminum seals.

Storage and Stability
Store intact vials refrigerated at 2º to 8ºC (36º to 46ºF), protected from light. Do not freeze. Shelf-life surveillance of the intact vials is ongoing. The administration of undiluted and diluted solutions of nivolumab must be completed within 24 hours of preparation. If not used immediately, the infusion solution may be stored up to 24 hours in a refrigerator at 2º to 8ºC (36º to 46ºF) and a maximum of 4 hours of the total 24 hours can be at room temperature (20º to 25ºC, 68º to 77ºF) and room light. The maximum 4-hour period under room temperature and room light conditions includes the product administration period. Caution: The single-use dosage form contains no antibacterial preservative or bacteriostatic agent. Therefore, it is advised that the product be discarded 8 hours after initial entry.

Preparation
Nivolumab injection can be infused undiluted (10 mg/mL) or diluted with 0.9% Sodium Chloride Injection, USP or 5% Dextrose Injection, USP to protein concentrations as low as .35 mg/mL. Care must be taken to assure sterility of the prepared solution as the product does not contain any antimicrobial preservative or bacteriostatic agent. All vials used to prepare an infusion must have the same batch number. No incompatibilities between nivolumab and polyvinyl chloride (PVC), non-PVC/non-DEHP (di(2-ethylhexyl)phthalate) IV components, or glass bottles have been observed.

For administration of Nivolumab, please refer to Section 9.1 Study Drugs.

6.0 CRITERIA FOR SUBJECT ELIGIBILITY

6.1 Subject Inclusion Criteria

- Male or female age ≥ 12 years at the time of informed consent

- Be capable, willing, and able to provide written informed consent/assent. For patients < 18 years of age, their parents or legal guardians must sign a written informed consent. Assent, when appropriate, will be obtained according to institutional guidelines.
- Be willing to comply with clinical trial instructions and requirements
- Patients ≥ 18 years must be willing to comply with the mandatory biopsies.
- Patients must have a histologically confirmed metastatic and/or locally advanced sarcoma by the enrolling institution
- For histological specific cohorts, patients must have confirmed metastatic and/or locally advanced osteosarcoma, chondrosarcoma, undifferentiated pleomorphic sarcoma/malignant fibrous histiocytoma)high grade myxofibrosarcoma (UPS/MFH/MFS), vascular sarcoma, alveolar soft part sarcoma (ASPS), dedifferentiated/pleomorphic liposarcoma, Small Blue Round Cell/Synovial, or leiomyosarcoma (LMS) by the enrolling institution. Note: Patients with confirmed sarcoma with histologies not defined by the above cohorts will be enrolled into the “Other” cohort.
- Adequate performance status:
    - Participants ≥16 years - ECOG 0 or 1/KPS 100-70%
    - Participants 12-15 years - Lanksky 100-70%
- Patients must have at least one prior line of systemic therapy (e.g. chemotherapy, immunotherapy, targeted or biological therapy) for their sarcoma if standard treatment is appropriate. Treatment naïve patients may be enrolled if they have refused standard systemic treatment. Prior adjuvant therapy will not count provided it was completed more than 6 months previously.
- Presence of measureable disease per RECIST v1.1.Target lesions must not be chosen from a previously irradiated field unless there has been radiographically and/or pathologically documented tumor progression in that lesion prior to enrollment.
- On echocardiogram, documented left ventricular ejection fraction >45%. Patients may instead have a multigated acquisition (MUGA) scan instead of transthoracic echocardiogram (TTE).
- Adequate organ function as defined as per **Table 2 (below)**:
- Women of childbearing potential (WOCBP) † must have a negative urine or serum pregnancy test at screening and ≤ 72 hours prior to day 1 of study treatment. If the urine pregnancy test is positive or cannot be confirmed as negative, a serum pregnancy test will be required.

    † A woman of childbearing potential is a sexually mature female who: has not undergone a hysterectomy or bilateral oophorectomy; or has not been naturally postmenopausal for at least 24 consecutive months (i.e. has had menses at any time in the preceding 24 consecutive months).

- Male patients with WOCBP partners and female patients of childbearing potential must be willing to use an adequate method of contraception as outlined in Section 11.9, for the course of the study through 7 months (male participants) or 5 months (female participants) after the last dose of study medication.
  Note: Abstinence is acceptable if this is the usual lifestyle and preferred contraception for the patient.

Table 2: Adequate Organ Function

System	Laboratory Value
**Hematological**	
Absolute neutrophil count (ANC)	≥1,500 /mcL
Platelets	≥100,000 / mcL
Hemoglobin	≥9 g/dL or ≥5.6 mmol/L
**Renal**	
Serum creatinine **OR**<br>Measured or calculated[a] creatinine clearance<br>(GFR can also be used in place of creatinine or CrCl)	≤1.5 X upper limit of normal (ULN) **OR**<br><br>≥60 mL/min for patient with creatinine levels > 1.5 X institutional ULN
**Hepatic**	
Serum total bilirubin	≤ 1.5 X ULN **OR**<br>Direct bilirubin ≤ ULN for patients with total bilirubin levels > 1.5 ULN
AST (SGOT) and ALT (SGPT)	≤ 2.5 X ULN **OR**<br>≤ 5 X ULN for patients with liver metastases
Albumin	≥2.5 mg/dL
**Coagulation**	
International Normalized Ratio [34] or Prothrombin Time [35]	≤1.5 X ULN unless patient is receiving anticoagulant therapy<br>as long as PT or PTT is within therapeutic range of intended use of anticoagulants
Activated Partial Thromboplastin Time (aPTT)	≤1.5 X ULN unless patient is receiving anticoagulant therapy<br>as long as PT or PTT is within therapeutic range of intended use of anticoagulants
[a]Creatinine clearance should be calculated per institutional standard.	

6.2 Subject Exclusion Criteria

- History of unstable or deteriorating cardiac disease within the previous 6 months prior to screening including but not limited to the following:
  - Unstable angina or myocardial infarction.
  - Congestive heart failure (New York Heart Association [NYHA] Class III or IV).

    - Uncontrolled clinically significant arrhythmias.
- Evidence of clinically significant interstitial lung disease or has known history of, or any evidence of active, non-infectious pneumonitis.
- Has known active central nervous system (CNS) metastases and/or carcinomatous meningitis. Patients with previously treated brain metastases may participate provided:
    - No current brain metastasis lesion greated than 2 cm. Patients with prior metastasis lesions greater than 2 cm that have been removed by surgical and/or radiotherapy may be enrolled if the lesion has been stable since surgery or radiotherapy.
    - No new or progressing brain metastatis of any size
    - No stereotactic radiation or craniotomy within 4 weeks of Cycle 1 Day 1
    - They are stable (without evidence of progression by imaging for at least four weeks prior to the first dose of trial treatment)
    - No clinically significant symptoms secondary to brain metastases (This exception does not include carcinomatous meningitis which is excluded regardless of clinical stability.)
- Evidence of clinically significant immunosuppression such as the following:
    - Primary immunodeficiency state such as Severe Combined Immunodeficiency Disease
    - Concurrent opportunistic infection
    - Receiving systemic immunosuppressive therapy (> 2 weeks) including oral steroid doses > 10 mg/day of prednisone or equivalent within 2 months prior to enrollment. (Steroids for pre-medication for imaging studies are allowed.)
- History or evidence of symptomatic autoimmune disease (e.g., pneumonitis, glomerulonephritis, vasculitis, or other), or history of active autoimmune disease that has required systemic treatment (i.e., use of corticosteroids, immunosuppressive drugs or biological agents used for treatment of autoimmune diseases) in past 2 years prior to enrollment. Replacement therapy (e.g., thyroxine for hypothyroidism, insulin for diabetes or physiologic corticosteroid replacement therapy for adrenal or pituitary insufficiency) is not considered a form of systemic treatment for autoimmune disease.
- Known history of human immunodeficiency virus (HIV) (HIV 1/2 antibodies) disease
- Patients known to be positive for active Hepatitis B (HBsAg reactive), or Hepatitis C (HCV RNA (qualitative) is detected)
- Prolonged QTcF > 450 ms for men and > 470 ms for women at Screening.
- Patients who have received a live vaccine within 30 days of the start date of the planned study therapy. *Note: Seasonal influenza vaccines for injection are generally inactivated flu vaccines and are allowed; however intranasal influenza vaccines (e.g., Flu-Mist®) are live attenuated vaccines, and are not allowed.*
- Has a known history of active TB (Bacillus Tuberculosis)

- Is currently participating and receiving study therapy or using an investigational device or has participated in a study of an investigational agent and received study therapy or used an investigational device within 4 weeks of the first dose of treatment.
- Has had a prior anti-cancer monoclonal antibody (mAb) within 4 weeks prior to study Day 1 or who has not recovered (i.e., ≤ Grade 1 or at baseline) from adverse events due to agents administered more than 4 weeks earlier.
- Has had prior chemotherapy, targeted small molecule therapy, or radiation therapy within 3 weeks prior to study Day 1 or who has not recovered (i.e., ≤ Grade 1 or at baseline) from adverse events due to a previously administered agent.
    - Note: Patients with ≤ Grade 2 neuropathy are an exception to this criterion and may qualify for the study.
    - Note: If patient received major surgery, they must have recovered adequately from the toxicity and/or complications from the intervention prior to starting therapies.
- Hypersensitivity to nivolumab or any of its excipients.
- Hypersensitivity to NKTR-214 or any of its excipients.
- Need for > 2 antihypertensive medications for management of hypertension (including diuretics).
- Women who are pregnant or breast feeding

7.0 RECRUITMENT PLAN

The clinical trial will be listed on the clinicaltrials.gov website and on the websites of participating institutions. Patients will be identified through internal referrals and external referrals by Medical and Surgical Oncologists, nationally and internationally. At MSK, patients will be recruited through the Sarcoma Disease Management Team. The Sarcoma Service and the Sarcoma Disease Management Team each hold weekly interdepartmental meetings to identify study participants for open clinical trials. We will also discuss the trial and patient recruitment with several Sarcoma patient support groups. Pediatric patients will be recruited through the department of pediatrics in consultation with the principal investigator. The principal investigator will be available to all patients for further questions and information through a contact number, which will be provided on the consent form. Similar recruitment procedures will be conduct at participating institutions.

All eligible patients, regardless of sex and race, will be approached for participation. The investigators are aware of the NIH policy concerning inclusion of women and minorities in clinical research populations.

During the initial conversation between the investigator/research staff and the patient, the patient may be asked to provide certain health information that is necessary to the recruitment and enrollment process. Participation in the study is completely voluntary. Patients will be required to read, agree to, and sign an IRB-approved informed consent form prior to registration on this trial. Patients will not receive payment for their participation on this study. Patients are free to withdraw from the study without consequence at any time.

8.0 PRE-TREATMENT EVALUATION

Screening

All aspects of the screening evaluation must be completed prior to entering the study, unless otherwise noted:

The following must be completed within 28 days before starting treatment:

- Signed informed consent and assent, if applicable, for study participation. Confirmation of disease: documented presence of metastatic and/or locally advanced sarcoma with RECIST v1.1 measureable disease.
- Full medical history including all active conditions, and any conditions diagnosed within the past 10 years that are considered to be clinically significant to the investigator
- Review of concomitant medications including any prior medications taken by the patient within 28 days before starting treatment
- Physical exam (including height and weight)
- Vital signs (pulse, blood pressure, temperature, respiratory rate, and oxygen saturation). Note: height may be documented at any time prior to registration
- ECOG, KPS, or Lansky performance status
- Complete blood count with differential, including lymphocyte and eosinophil count
- Comprehensive metabolic panel (albumin, alkaline phosphatase, total bilirubin, bicarbonate, BUN, calcium, chloride, creatinine, glucose, potassium, total protein, SGOT [AST], SGPT [ALT], sodium), phosphorus, magnesium, amylase, and lipase.
- PT (or INR) and aPTT (for screening only)
- Thyroid function tests (TSH, T4 free, T3)
- HIV, Hepatitis B and Hepatitis C tests
- Urinalysis (dipstick)
- 12-lead electrocardiogram (ECG)
- Echocardiogram or MUGA scan
- Mandatory tumor biopsy for participants ≥ 18 years

- CT scan of the chest, abdomen and pelvis (with or without contrast)
- CT scan or MRI of brain if applicable.
- Serum β-HCG or urine pregnancy test for women with child-bearing potential. If the urine test is positive or cannot be confirmed as negative, a serum pregnancy test will be required

The following must be completed within 72 hours before starting treatment:

- Serum β-HCG or urine pregnancy test for women with child-bearing potential. If the urine test is positive or cannot be confirmed as negative, a serum pregnancy test will be required

9.0 TREATMENT/INTERVENTION PLAN

9.1 Study Drugs

Treatment will be administered on an outpatient basis. Reported adverse events and potential risks are described in Section 11.0. Modifications and dose delays due to toxicity are described further in Section 11.4. The schedule of evaluations and interventions is described in Section 10.

One treatment cycle will consist of 21 days. Patients will start both study drugs on day one of the first cycle. Treatment will include nivolumab and NKTR-214 on day 1 and every 3 weeks thereafter. NKTR-214 will be administered first followed by nivolumab.

A delay in the start of subsequent cycles due to holidays, weather, or other circumstances will be permitted up to 7 days. Dose delays will also be allowed to permit for patients to recover from related and unrelated adverse events as specified in Section 11.0. Patient's subsequent dosing schedule will be based off the date patient resumes study drug.

Administration of NKTR-214

- NKTR-214 (0.006mg/kg) will be administered every 3 weeks via an IV infusion over at least 30 (-5) minutes.
- Follow the vital sign monitoring and hydration guidelines in Section 9.5
- A Patient Wallet Card (Appendix 2) is available that contains important safety information.
- Should the patient experience hypotension at any time during a cycle, IV fluid administration has been effective. Vasopressors have not been required to manage hypotension. Patients with adrenal insufficiency requiring corticosteroid supplementation may benefit from additional short-term corticosteroid use of up to 5 mg/day of prednisone for the management of hypotension.

Administration of Nivolumab

- Nivolumab (anti-PD-1) 360mg will be administered every 3 weeks as an IV infusion over at least 30 (-5) minutes.
- Administer via intravenous infusion through an intravenous line containing a 0.2-micron to 1.2-micron pore size, low-protein-binding polyethersulfo ne membrane in-line filter. It is not to be administered as an IV push or bolus injection. Flush the intravenous line with 0.9% NaCl after each dose. Do not mix nivolumab with, or administer as an infusion with other medicinal products.
- Nivolumab should be administered within 30 (± 5) minutes after NKTR-214 administration.

9.2 Dose rationale

Dosing has been determined based on the on-going phase Ib study (NCT02983045).

Dosing for pediatric patients has been determined based on the on-going phase I/II study (NCT02304458).

Please refer to Section 3.3.

9.3 Duration of Therapy

Study treatment may continue until one of the following criteria applies:

- Clinical or radiographic disease progression as defined by RECIST 1.1. Patient may be allowed to continue treatment after progression if they are deriving clinical benefit (section 9.4).
- Intercurrent illness that prevents further administration of treatment
- Intolerance of study treatment
- Patient decides to withdraw from the study
- Physician decides to withdraw a patient from the study for a reason not listed here
- Pregnancy in patient
- The patient is lost to follow-up
- Inability of the patient to comply with the requirement of the protocol for treatment or evaluation.
- End of study, whichever occurs first.

9.4 Treatment Beyond Initial RECIST 1.1 Defined Progression of Disease

Due to the mechanism of action, patients may experience growth in existing tumors or the appearance of new tumors prior to maximal clinical benefit of the combination therapy of NKTR-214 and nivolumab. **The patient may be allowed to continue study treatment after initial RECIST 1.1 defined progression IF:**

a) They are assessed by the treating physician to be deriving clinical benefit and tolerating study treatment. The treating physician may consult with the MSK PI for help with assessing the patient.
b) There is an absence of symptoms and signs indicating clinically significant progression of disease.
c) There is no decline in patient's performance status.
d) There is an absence of symptomatic rapid disease progression requiring urgent medical intervention (e.g. symptomatic pleural effusion, spinal cord compression, etc).

Patients that meet these criteria and continue study treatment should discontinue study therapy upon further evidence of progression at the discretion of the treating physician.

9.5 General Concomitant Medication and Supportive Care Guidelines

Vital Signs Monitoring

Vital signs (VS) are to be monitored and recorded following the completion time of the nivolumab administration.

For Cycle 1 only, VS monitoring windows will be as follows:

- Cycle 1 Day 1 (dosing day) monitor every hour for 3 hours (+/- 15 mins (q1h x 3)
- Cycle 1 Day 3 monitor every hour for 2 hours (+/- 15 mins) (q1h x 2)

During Cycle 2 and beyond, on dosing days, monitor and record vital signs pre-dose and approximately 1 hour after administration of nivolumab.

If the patient experienced a Grade ≥ 2 infusion-related reaction or hypotension on the dosing day, the patient may be monitored overnight at the discretion of the Investigator. Longer periods of monitoring may be implemented at the discretion of the Investigator.

Hydration Guidelines

For Cycle 1 and 2, administer at least 1 L of IV fluid on Days 1 for patients ≥ 18 years Recommend at least 2 liters per day of self-administered oral hydration (e.g., fluid containing electrolytes) on Days 2 to 5. Pediatric participants ages 12 – 17 will be administered 1500 mL/m^2 IV fluids on Day 1 and are recommended at least 1500 mL/m^2 per day of self-administered oral hydration of Days 2 to 5.

During Cycle 3 and beyond, recommend at least 2 liters per day of self-administered oral hydration on Days 1 to 5. Pediatric participants are recommended 1500 mL/m^2 of oral hydration per day.

Advise patients to restrain from strenuous activity and avoid long hot showers and saunas for Days 1 to 5 of every cycle.

Per clinical judgment, IV fluids may be administered in any cycle. The investigator may decide to forego administering IV fluids to a patient if this is deemed in the best interest of the patient (e.g., evidence of fluid overload).

Blood pressure Management
Consideration of discontinuation of antihypertensive therapy and drugs with hypotensive properties, prior to administration of NKTR-214.

Supportive Care
Best supportive care and treatment will be given as appropriate to each patient as per Clinical Center and ASCO guidelines (antiemetics, antibiotics, packed red blood cell and platelet transfusions, nutritional support, non-radiation palliative treatment for pain).

Steroids
Doses up to 10mg of prednisone daily (or equivalent) will be allowed concurrently with continuous treatment with NKTR-214 and nivolumab for participants ≥18 years. Participants < 18 year should dose at 5 mg/m^2/day of prednisone daily (or equivalent) with a maximum dose of 10mg. If the patient requires corticosteroid dosing of >10 mg prednisone daily (or equivalent) and/or other immunosuppressive medication for related toxicities, NKTR-214 and nivolumab dosing must be held until the corticosteroid dose has decreased to ≤10 mg prednisone daily (or equivalent) and the administration of the other immunosuppressive medication has discontinued. Steroids for pre-medication for scans are allowed.

Radiation

Radiation therapy is only allowed during the study if required for palliation of symptoms due to underlying sarcoma.

Surgery & other local interventions
Patients must not schedule any elective surgeries during the treatment period and for at least 30 days after the last administration of study drugs. If a patient undergoes any unexpected surgery during the course of the study, all study treatments must be withheld and the investigator or designee should notify the sponsor' as soon as possible. A patient may be allowed to resume study drugs if s/he agrees to restart study therapy.

9.6 Drug Interactions

9.6.1 NKTR-214
There are no known drug interactions identified at this time.

9.6.2 Nivolumab
Medications or vaccinations specifically prohibited in the exclusion criteria are not allowed during the ongoing trial. If there is a clinical indication for one of these or other medications or vaccinations specifically prohibited during the trial, discontinuation from trial therapy or vaccination may be required. The investigator should discuss any questions regarding this with the MSK PI, who will in turn escalate any questions to BMS when appropriate. The final decision on any supportive therapy or vaccination rests with the investigator and/or the patient's primary physician.

All concomitant medications received within 28 days before the first dose of trial treatment and 30 days after the last dose of trial treatment should be recorded. Concomitant medications administered within 30 days after the last dose of trial treatment should be recorded for SAEs and ECIs.

Patients are prohibited from receiving the following therapies during the Screening and Treatment Phase (including retreatment for post-complete response relapse) of this trial:

- Antineoplastic systemic chemotherapy or biological therapy
- Immunotherapy not specified in this protocol
- Chemotherapy not specified in this protocol
- Investigational agents
- Radiation therapy
  Note: Radiation therapy to a symptomatic lesion may be allowed at the investigator's discretion.

- Live vaccines within 30 days prior to the first dose of trial treatment and while participating in the trial. Examples of live vaccines include, but are not limited to, the following: measles, mumps, rubella, varicella/zoster, yellow fever, rabies, BCG, and typhoid vaccine.
- Systemic glucocorticoids for any purpose other than to modulate symptoms from an event of clinical interest of suspected immunologic etiology. The use of physiologic doses of corticosteroids are allowed.

Patients who, in the assessment by the investigator, require the use of any of the aforementioned treatments for clinical management should be removed from the trial. Patients may receive other medications that the investigator deems to be medically necessary.

There are no prohibited therapies during the Post-Treatment Follow-up Phase.

1.0 EVALUATION DURING TREATMENT/INTERVENTION

Table 3. Study Calendar Evaluations during the treatment/intervention must be performed as outlined in table 3 below.

**Assessment Period**	**Screening**	**Cycles 1 and 2**			**Cycle 3 and Beyond** [13]	**Post-Treatment**	
**Study Days**	**Day -28 to 1**	**Day 1**	**Day 3**[12]	**Day 8**[16]	**Day 1**	**End of Treatment**[14]	**Follow Up Q3 months**[15]
Informed Consent	X						
Medical History	X						
Physical Examination[1]	X	X	X	X[15]	X	X	X
Vitals [2]	X	X	X	X[15]	X	X	
ECOG/KPS	X	X	X	X[15]	X	X	
Review of Concomitant Meds	X	X	X	X[15]	X		
Review of Adverse Events		X	X	X[15]	X		
Hematology[3] and Chemistry[4]	X	X	X	X[15]	X	X	X
HIV, Hepatitis B and Hepatitis C	X						
Pregnancy Test [5]	X	X			X	X	
Urinalysis (Dipstick)	X				X	X	
ECG	X	X			X		
ECHO/MUGA	X					X	
Research Bloods[6]		X[6]		X[6]	X[6]	X[6]	
PKs[7]		X[7]	X[7]	X[7]	X[7]		
Research Biopsy[8]	X[8]	X[8]				X[8]	
Tumor Assessment[9]	X				X[9]		X
Administer Fluids[10]		X[10]	Days 2-5		Days 1-5		
NKTR-214 [11]		X			X		
Nivolumab [11]		X			X		
Survival Status							X[15]

1. Physical Examination must include height and weight.
2. Vital signs include: pulse, blood pressure, temperature, respiratory rate, and oxygen saturation.
3. Hematology must include CBC with differentials and lymphocyte and eosinophil count.
4. Chemistry must include comprehensive metabolic panel (albumin, alkaline phosphatase, total bilirubin, bicarbonate, BUN, calcium, chloride, creatinine, glucose, potassium, total protein, SGOT [AST], SGPT [ALT], sodium, magnesium, phosphorus), amylase, lipase, and thyroid function tests (fT4, TSH, and T3). PT (or INR) and aPPT should be tested for screening only.
5. Serum β-HCG or urine pregnancy test for women with child-bearing potential. If the urine test is positive or cannot be confirmed as negative, a serum pregnancy test will be required. Screening pregnancy tests must be performed within 72 hours of starting treatment.
6. Research blood tests will be performed pre-dose on Day 1 and 8 of Cycles 1 and 2. Research blood test will also be taken pre-dose on Day 1 of Cycle 3, and pre-dose on Day 1 of all odd-numbered cycles thereafter, and end-of-treatment visit (+/- 3 days). Additional research bloods will be collected pre-dose on Day 1 of Cycle 8, Cycle 16, Cycle 24, Cycle 36 and Cycle 48. Please refer to Section 10.2 Research Blood Sample Collection for details
7. PKs samples will only be completed for patients < 18 years. Timed blood samples will be collected pre-dose and post infusion on Cycle 1 Day 1, 3, and 8. Timed blood samples will also be collected pre-dose and post infusion Cycle 2 Day 1 and every cycle thereafter.
8. All study participants ≥ 18 years will undergo mandatory tumor biopsies at baseline within 28 days of starting on treatment and before Cycle 2 Day 1 (±1 week). Patients will also be asked to participate in an optional biopsy at progression (±1 week). Archival tissue will be collected if available for participants <18 years.
9. Patients will be evaluated radiographically at baseline, week 8 (+/- 7 days) and every 8 weeks (+/- 7 days) subsequently until week 56 and then every 12 weeks thereafter or as per the discretion of the treating investigator. Imaging studies will include CT of chest, abdomen and pelvis (with or without contrast) and MRI of the affected area if deemed necessary by the treating physician. CT scan or MRI of brain will be done at screening, if applicable.
10. Mandatory 1L IV fluids for participants ≥ 18 years on Cycle 1 Day 1 and Cycle 2 Day 1 Only. For participants < 18 years please see section 9.5 for hydration guidelines. On Cycle 1 and Cycle 2, it is recommended that at least 2L of fluid are self-administered orally [e.g., fluid containing electrolytes] on Days 2-5. For Cycles 3 and beyond, at least 2 liters of fluid per day should be self-administered orally for Days 1 to 5. Advise patients to restrain from strenuous activity and avoid long hot showers and saunas for Days 1 to 5 of every cycle. Please refer to Section 9.5.
11. Treatment window is ± 3 days. NKTR-214 will be administered first before nivolumab. NKTR-214 will be administered over 30 minutes (+/- 5 minutes). Nivolumab will be given 30 minutes (+/- 5 mins) after NKTR-214 administration. Nivolumab will be administered over 30 minutes (+/- 5 minutes). Patients must be monitored for 1 hour post NKTR-214 dosing.
12. Day 3 visit only on Cycle 1 (+/-1 day).
13. Patients who achieve response/stability of disease lasting for at least 12 months will be followed every 2 months (±2 weeks) thereafter (includes all study assessments and evaluations).
14. End of Treatment visit, should be within 30 days (± 7 days) of last dose of NKTR-214 or Nivolumab, whichever is later.
15. Survival status will be confirmed every 12 weeks (± 28 days) following the end of treatment visit until death, the patient withdraws consent, or 12 months after the end of treatment, whichever occurs first. Patients that complete 24 months of treatment with a CR or PR will have follow up assessment every 12 weeks (+/- 14 days). See section 4.1 for details.
16. Day 8 office visit assessments (physical exam, ECOG, review of conmeds and AEs) are done on Cycle 1 ONLY. Cycle 2 Day 8 assessments are vitals, routine bloods and research bloods ONLY. Day 8 routine bloods only include CBC, Comp, PT (or INR) and aPTT. All assessments on Day 8 (+/- 2 days Window).

10.1 Required Laboratory Parameters for Treatment

A new dose of therapy may be initiated provided that the patient meets the following criteria on the day of treatment:

- ANC ≥ 1.0 K/mcL
- Platelets > 100 K/mcL

- All grade ≥ 2 non-hematologic adverse events (except for fatigue, nausea and vomiting, or laboratory values that are not clinically significant) must have resolved to CTCAE grade ≤ 1

10.2 Tumor and blood samples for correlative/companion studies

All patients ≥18 years enrolled on this study will undergo paired tumor biopsies and blood sample collection for research purposes (correlative/companion studies) where feasible.

Biopsies for research purposes will be done at baseline (within 28 days of the first dose of NKTR-214 and/or nivolumab) and at Week 3 (Cycle 2 Day 1) in all patients enrolled in this study where feasible. Up to 6 cores from each tumor biopsy site should be taken where feasible and safe to do so. An optional biopsy will be offered to all patients at the time of progression. Biopsy procedures will otherwise be performed as per institution guidelines.

Archival tissue sample will be collected if available for participants < 18 years.

Tumor biopsy collection

Given the requirement for fresh and frozen tissue, FFPE archival samples cannot be substituted for the baseline or subsequent biopsies.

Tumor biopsies for research purposes will be done at baseline (within 28 days of the first dose of NKTR-214 and/or nivolumab) and at Week 3 (Cycle 2 Day 1) in all patients enrolled in this study where feasible. An optional biopsy will be offered to all patients at the time of progression.

The same metastatic tumor site will be biopsied at each time point, if feasible. Cores will be obtained with 18-gauge needles where appropriate and be of at least 1 cm in length. Cores will be representative of tumor, and targeted to the de-differentiated component in the case of de-differentiated liposarcoma (DD-LPS).

The quality, viability and tumor content of biopsies will be confirmed by the on-call clinical pathologist at the enrolling institution at the time of retrieval.

The goal will be to extract 6 cores, with a minimum of one formalin-fixed, for later paraffin embedding (FFPE), one fresh (stored in RPMI media on ice) and one flash frozen in liquid nitrogen and transported in dry ice. If it is not possible to obtain 6 cores then prioritize the sample type accordingly:

1 FFPE
2 Fresh

3 Flash frozen
4 Fresh
5 Flash frozen
6 Flash frozen

If any extra cores are obtained they will be flash frozen with liquid nitrogen. Biopsy procedures will otherwise be performed as per institutional guidelines.

Samples will be labeled using an adherent, liquid nitrogen proof label with the following information:

1. Procurement date
2. Study IRB number
3. Study patient number
4. Time point (baseline or on-treatment)
5. Biopsy site

Each FFPE sample is to be placed in a pathology sample container pre-filled with formalin and delivered to the central pathology lab.

Each fresh sample is to be placed in a 50ml Falcon tube with 20 ml of RPMI media, labeled and the cryovial placed in a sample bag and the sample bag placed within ice.

Each flash frozen sample is to be placed within an individual cryovial, labeled, and the vial immediately placed in liquid nitrogen for 2 minutes or longer to snap-freeze the tissue. These vials can be transported in either liquid nitrogen or in dry ice. Samples are to be delivered to the Immune Monitoring Facility as detailed below.

Research Blood Sample Collection

PHARMACOKINETIC ASSESSMENT FOR PEDIATRIC PATIENTS

Whole blood samples (2mL per timepoint) will be obtained for pharmacokinetic assessments throughout the course of the study. For Cycle 1 only, blood will be collected on Days 1, 3, and 8. Pharmacokinetic samples will occur at the following times after initiation of NKTR-214 dosing:

30 min ± 5 min (end of NKTR-214 infusion), 1.5 hr ± 10 min, 3 hr ± 10 min, 6 hr + 2 hrs, 48 hr ± 4 hrs (Day 3), and 168 hr ± 4 hrs (Day 8).

For Cycle 2 and thereafter, pharmacokinetic samples will occur on Day 1 predose and 30 mins ± 5mins (end of the NKTR-214 infusion).
2 ml of blood will be collected in green top sodium heparin vacutainer tube. The tubes will be gently inverted 8-10 times. The tube will be spun in a refrigerated centrifuge (2-8°C) at 1100 RCF(g) for 15 minutes.These sample should be maintained at 2-8°C while handling. Plasma will be distributed into (2) 1 ml Matrix™ screwtop tubes with NKTR-214-AC labels and (2) 1 ml Matrix™ screwtop tubes with NKTR-214-RC labels. Tubes will be frozen immediately at -70°C or lower and shipped same day to Nektar Therapeautics.

IMMUNOGENICITY TESTING FOR NKTR-214

Research bloods for NKTR-214 immunogenicity testing will be collected pre-dose on Cycle 1 Day 1, pre-dose on Cycle 2 Day 1, and pre-dose on Day 1 of all odd-numbered cycles thereafter, and end of treatment visit.

At each collection time point of immunogenicity testing for NKTR-214 the following sample will be collected:

6 ml of blood will be collected in red top serum tube. The tubes will be gently inverted approximately 5 times. The tube will be allowed to rest for 15-30 minutes in upright position at room temperature. The tube would then be spun in a refrigerated centrifuge (2-8°C) at 1000-2000 RCF(g) for 10 minutes. Serum (Clear upper layer) will be immediately removed with a transfer pipette. Serum will be distributed in aliquots of 500uL into the five labeled Cryovials. Vials will be stored immediately at -70 ºC or lower.

IMMUNOGENICITY TESTING FOR NIVOLUMAB:

Research bloods for nivolumab immunogenicity testing will be collected pre-dose on Cycle 1 Day 1, predose Cycle 2 Day 1, and predose Cycle 3 Day 1, Cycle 8 Day 1, Cycle 16 Day 1, Cycle 24 Day 1, Cycle 36 Day 1 and Cycle 48 Day 1.

At each collection time point of immunogenicity testing for nivolumab the following sample will be collected:

3.5 ml of blood will be collected in SSTred top tube. The tubes will be gently inverted approximately 5 times. The tube will be allowed to clot for 30-45 minutes in upright position at room temperature. Centrifuge at room temperature for 10 minutes (swing out) or 15 minutes (fixed) at 1100-1300 Xg until clot and serum well separated. Transfer serum from 3.5mL SST into appropriately labelled screw capped 4ml tube. Store serum samples immediately (approximately within 2 hours of collection) -70

ºC or lower to ensure stability of the samples until they are shipped on dry ice to central lab every 2 months.

MSK RESEARCH BLOODS:
Blood samples for research purposes will be obtained at pre-dose on Cycle 1Day 1, Cycle 1 Day 8, Cycle 2 Day 1, Cycle 2 Day 8, and Day 1 of Cycles 3, 5, 11, 15 and end of treatment visit (+/- 3 days).

At each routine research blood collection time point the following samples will be collected:

4x8ml of peripheral venous blood will be collected in Sodium Heparin with FICOLL CPT tubes. Specimens will be placed in a biohazard bag and kept at room temperature.

Samples will be labeled with the following information:

1. Procurement date
2. Study IRB number
3. Study patient number
4. Time point (baseline or week-X)

Research blood samples and tumor biopsy samples, along with completed requisition forms, are to be transported to the Immune Monitoring Facility in the Zuckerman Research Bldg, Room Z-1513. At least 24 hr advanced notification prior to biopsy or blood collection must be provided by email to the IMF contacts below or entered in the IMF shared calendar (zzCAL_LAB_Clinical_Trials/Shared Calendar), with clinical site location and contact information from which samples are arriving indicated. Samples must be delivered between the hours of 9 am-4 pm to a member of the lab.

Rosemarie Ramsawak <ramsawar@mskcc.org> 646-888-3106
Zhenyu Mu <muz@mskcc.org> 646-888-2114
Kevin Crawford <crawfork@mskcc.org> 646-888-3106
Luisa Caro <carol@mskcc.org> 646-888-3106

Tumor Based Biomarkers
Interrogation of immune responses within the tumor microenvironment of sarcomas before and after treatment with NKTR-214 and nivolumab.

For each resected specimen, up to 6 cores of tissue will be obtained for tumor immune correlative studies.

a) **Density of immune infiltration and immune checkpoint:** IHC will be used to assess the number and composition of immune infiltrates in order to define the immune cell subsets present within the tumor before and after exposure to NKTR-214 and nivolumab. PD-L1, PD-1 expression within the tumor before and after exposure to NKTR-214 and nivolumab will also be determined by IHC.

   The IHC assays will be performed using but are not limited to the following markers: CD3, CD4, CD8, CD25, CD28 CD45RA, CD68, CD69, CCR7, IDO-1, kynurenine, PD-L1, PD-1, CD137, FOXP3, LAG-3, TIM-3, and ICOS.

   PD-L1 expression by immunohistochemistry staining of archival tumor specimens will be performed with using a DAKO immunohistochemistry-based assay.

b) **Phenotype and function of TIL in human sarcomas:** Multiparameter flow cytometry (MFC) performed on freshly dissociated tumor tissue will be used to characterize the tissue T cell populations. We will study the functional significance of PD-1 expression on T cells isolated from sarcomas. Using intracellular cytokine staining (ICS) and MFC analysis on freshly isolated TIL, we will identify the T cell cytokine profiles in accordance to their immune checkpoint profile (PD-1, LAG-3, TIM-3.) A range of cytokines representing key lymphocyte subgroups (e.g., Th1, 2 or 17) such as IL-2, TNF-α, IFN-γ IL-17 among other inflammatory mediators will be assessed. Functional T cell markers including granzyme B and perforin as well as activation markers such as CD62L, CD44, CD69 will be also included in our analysis.

c) **Tumor Biopsy gene expression profiling**: Fresh tumor biopsy will be examined for RNA gene and protein expression by NanoString technology (nCounter® RNA: Protein, PanCancer Immune Profiling Panel) and/or qRT-PCR to detect expression of immune related genes. This panel includes 770 immune related genes including: 109 genes related to cell surface markers for 24 different immune cell types and populations, 30 genes for commonly studied cancer/testis (CT) antigens, over 500 genes for measuring immune response and 40 reference genes.

d) **Neoantigen analysis:** Massively parallel sequencing of the whole exome of the tumor tissue and normal blood will be performed for patients who opt in. Genomic DNA will be captured via solution-based hybrid selection and

sequenced on the Illumina HiSeq platform by the Genomics Core at MSKCC. The sequencing data will be aligned by the Bioinformatics Core Tumor, using Somatic Sniper, Somatic Indel Detector and Mutect software.

e) **Next generation sequencing for T-cell receptor clonality in tumor-infiltrating lymphocytes (TIL):** Samples will be analyzed using highthroughput sequencing of the variable β-chain of the T cell receptor (TCR) to characterize the expansion and clonality of the T-cell repertoire in TILs.

<u>Peripheral blood studies</u>

Blood samples will be taken prior to initiation of study therapy and on designated time points post-treatment. These samples will be analyzed for:

a.) **Immunophenotyping and Functional Analyses:**

1. Samples will be analyzed by flow cytometry to study the effects of NKTR-214 and nivolumab on various peripheral blood immune cell subsets including, but not limited to T cell subsets (activated, memory and regulatory T cells).

2. To explore whether inhibition of PD-1 will restore T cell activation and function, peripheral blood mononuclear cells (PBMCs) will be isolated and cryopreserved. Assays of the functional status of effector T cells will be performed, including, but not limited to assays for interferon-gamma (IFN-γ) and granzyme B. This assay will use a non-specific stimulus including but not limited to anti-CD3 and anti-CD28 and would allow for the comparison of the effect of PD-1 blockade alone on T cell function.

b) **Soluble Factors:** Baseline and on-treatment serum levels of chemokines, cytokines and other immune mediators will be assessed by techniques that may include but are not limited to ELISA or multiplex assays. Analytes may include, but are not limited to IFN-γ, IL-12, IL-10, soluble MICA, C-reactive protein, soluble PD-1 and soluble PD-L1.

b.) **Next generation sequencing for T-cell receptor clonality in peripheral blood:** Samples will be analyzed using highthroughput sequencing of the variable β-chain of the T cell receptor (TCR) to characterize the expansion and clonality of the T-cell repertoire in peripheral blood mononuclear cells.

c.) **Immunogenicity samples for NKTR-214 and nivolumab:** Validated assays will be used for the determination of anti-drug antibodies to NKTR-214 and nivolumab in human serum. Sample volume will be sufficient to store aliquots

for future testing. Only patients who receive at least 1 dose of NKTR-214 and nivolumab and who provide at least 1 post-treatment sample will be evaluated. Immunogenicity results will be analyzed descriptively by summarizing the number and percentage of patients who develop detectable anti-NKTR-214 antibodies. Samples confirmed positive may also be evaluated for neutralizing antibody activity.

Table 4. Exploratory Biomarkers

Specimen Collected	Collection Tube	Collection time points	Exploratory studies
			**Blood**
**Peripheral Blood Cells**	4 x 8mL CPT tubes	C1D1, C1D8, C2D1, C2D8 and Day 1 Cycle 3, 5, 11, 15 and EOT	1. Flow cytometric analyses to evaluate activated (HLA-DR+) and memory (CD45RA-) T cells<br>2. Flow cytometric analyses to evaluated peripheral blood leucocytes including CD4/CD25/FoxP3, CD4/CD8/CD45RA/CCR7, CD4/CD8/LAG3/PD-1/PD-L1, CD4/CD8/CD137/IDO-1Functional Status of effector T cells assays for interferon-gamma and granzyme B.<br>3. T-cell subsets and their activation status (CD8+ Teff/Treg ratio and ICOS expression
			**Blood Serum**

			1. Treatment modulation of serum levels of chemokines cytokines and other immune mediators by ELISA or other multiplex-based assay methods. Primary analysis includes interferon-gamma and IL-10.
	1 x 6 ml SST (red-top tube)	C1D1, C2D1, and Day 1 of Cycle 3, 8, 16, 24, 36, 48	Immunogenicity Testing Nivolumab
	1 x 6 ml SST (red-top tube)	C1D1, C2D1, and Day 1 of Each Odd Number Cycle and EOT	Immunogenicity Testing NKTR-214
**Tumor biopsy**		Baseline biopsy	**Neoantigen analysis**
			1. Fresh frozen tumor biopsy will be utilized for massively parallel whole exome sequencing. Available databases such as SNP effect (http://snpeff.sourceforge.net/will be used to determine which mutations are in coding regions and will affect amino acid sequence. NetMHC and the Immune Epitope Database will be utilized to predict MHC Class I binding and T cell interactions, respectively.
		Baseline, Week 3, Progression (optional)	**Characterization of Tumor Infiltrating Lymphocytes (TILs)**
			1. IHC to assess number and composition of immune infiltrates to define immune cell subsets present within tumor before and after exposure to treatment. CD3, CD4, CD8 and FOXP3 will be evaluated.<br>2. Immune cell phenotyping of freshly isolated TIL to evaluate Treg/Teff ratio (CD8+/FoxP3+ cells) and activations markers (ICOS, PD-1, CD69).
			**PD-L1 expression by IHC**
			1. DAKO immunohistochemistry-based assay for PD-L1
			**T-cell receptor sequencing**
			1. Highthroughput sequencing of the variable β chain of the T-cell receptor.

10.3 Future Unspecified Use of Biospecimens

The protocol includes an informed consent document and research authorization that meets statutory guidelines. Each participating site will have its own consent form meeting the requirements described in this section. The consent form will inform patients of the purpose of the bank, their rights in relation to it, and the safeguards in place to protect the confidentiality of their health information. The consent will state that

some of the biospecimens will be saved to use for future research. Pediatric patients will require re-consent at age 18 for future unspecified use of biospecimens

Type of future use

The consent specifically describes the types of future research that may be performed, including use of tissues to develop new drugs with cancer-associated molecular targets, development of cell lines, future use of cell lines to define cancer phenotype and (somatic) genotype, DNA sequence analysis of tumor compared to normal and identification of tumor-associated proteins as diagnostic or prognostic markers. It will be stated that researchers at MSK may either keep indefinitely or dispose of any leftover blood or tissues or other samples, including DNA that the samples contain. Blood and tissues will be stored with identifiers in secure tissue or fluid banks. It is stated that the samples could be lost or ruined because of mechanical failure, and that MSK cannot guarantee that samples will be stored indefinitely. The samples will be stored for as long as deemed useful for research purposes.

Consent for future use and re-contact

Patients are asked in a series of check boxes at the end of the consent if 1) they permit their biospecimen samples to be stored and used in future research to learn about or prevent cancer or side effects of treatment, or to develop new treatments; 2) if they permit their samples to be stored and used in future research to learn about, prevent, or treat diseases other than cancer; or 3) if they permit their samples, with personal identifiers protected, to be used for research about inherited genetic factors, 4) if they permit their samples to be used for genetic analysis of the tumor and normal tissue to learn about the causes of cancer, 5) participants are asked if they agree to be contacted in the future as part of research studies for additional health information or to be asked to participate in future biospecimen research studies and 6) if they consent to be contacted to discuss research findings which may come from their sample. Finally, if not available (e.g. deceased), if they wish to have their designee designated on the consent to be contacted.

Participants will not be provided with specific results of research tests performed on their collected human biologic specimens.

Pediatric patients will require reconsent at age 18 about the future use of biospecimens.

Use of identifiable information for genetic studies

In the course of this research it is possible that some patients whose tumors are analyzed through investigational “next-generation” profiling in a research (non-CLIA) environment will be found to have somatic or germline mutations in genes that are known to be associated with an increased risk of cancer or other diseases. It will be stated in the consent that the participants will not receive any specific results from research tests. The consent will tell participants that if they wish to have genetic testing

done for personal reasons than they should make an appointment with the MSK Clinical Genetics Service or Clinical Genetics Service at their site.

If in the course of this research a research finding is obtained that, in the opinion of the investigator, may be critical to the preventive care of the participant or their family, the investigator can communicate that finding to the MSK IRB Genomic Advisory Panel (GAP). The finding will be reviewed by the GAP to determine whether the incidental finding should be discussed with the participant. For MSK patients, in the event that the GAP determines that the finding should be discussed with the participant, and the participant has consented to be re-contacted, then the treating/consenting physician shall be contacted by the panel and asked to refer the participant to the Clinical Genetics Service for further discussion of the research finding.

The following information must be provided to GAP for review:

- Participant Name/MRN #
- Type of Biospecimen (tissue, blood, saliva)
- Incidental Finding
- Collection Protocol #
- Contact: ocrgapirb@mskcc.org

For non-MSK patients being treated at one of the participating institutions, if the GAP determines the finding to be reportable to the participant and the participant has consented to be re-contacted, results will be returned to the Site Principal Investigator via the study team. Site policies on returning these research findings to the patient should be followed.

We anticipate that other research assays may be incorporated into this protocol as technology evolves.

Voluntariness of research participation
It is stated that taking part in this tissue and blood bank is voluntary and patients have the right to withdraw at any time. Participation in the study will not impact on the clinical care patients receive.

Withdrawal
Participants may decide at a later date that they do not want identified blood and tissue samples to be stored in the tissue bank and /or used for future research. If participants decide to withdraw from the study, specimens that have not yet left the specimen archive will not be used in new studies and any remaining portions of samples that have not been used for research will be used only for clinical purposes or, if requested by the patient, destroyed. For specimens already shipped out from the archive, it may not be possible to locate the samples or stop already ongoing research. When a participant withdraws from the protocol, MSK's Protocol Participant Registration (PPR)

Office should be notified immediately. If a non-MSK participant withdraws from the study, the MSK study staff member will notify PPR. The withdrawal request will be documented in CRDB and the system updated accordingly. In addition, a note-to-file documenting the patient withdrew must be filed in his/her medical records.

Rights after death
The consent states that if the research participant dies or is unable to make his/her wishes known, all of their rights to decide about future uses of the blood or tissues will pass to the authorized representative of the estate. If there is no representative of the estate, the rights pass to the next of kin.

Risks of research participation
The greatest risk is release of information from health or research records in a way that violates privacy rights. MSK and any participating sites will protect records so that name, address, phone number, and any other information that identifies the participant will be kept private. It will be stated to the participant that the chance that this information will be given to an unauthorized individual without the participant's permission is very small.

Costs/compensation
There is no cost to the participant to enroll in this research. Tissue or blood obtained in this research may be used to make a cell line, and these may be patented or licensed and thus may have significant commercial value. The participant is informed that there are no plans to provide financial compensation for use of their human biologic specimens, nor are there plans for the participant to receive money for any new products, tests, and discoveries that might come from this research.

Biospecimen Privacy
Medical information is confidential. The participant's personal identity will not be used in reports that are written about the research. The MSK IRB/PB will review all requests for research performed involving biospecimens ascertained through this protocol. Blood and tissue samples may be stored with a code linked to the patient's medical record. The results of any research using blood or tissues will not be placed in the medical record.

The consent indicates that samples and genetic information collected may be shared with other qualified researchers and placed in online databases. An example of an online database is the NIH dbGAP database, which is monitored by the National Institutes of Health, and may be made accessible to investigators approved by the U.S. government. Such information will not include identifying information such as name. It is also stated in the Research Authorization (HIPAA Authorization) that research data (e.g. genomic sequence) may be shared with regulators. The requirements for

submission of genotype/phenotype data into the NIH dbGAP or any other public database will be followed as per the IRB SOP for Genomic Data Sharing.

Use of banked samples (at MSK)
When samples are to be analyzed, the individual investigator needs to write an IRB biospecimen protocol. This protocol is fast-tracked through MSK Research Council review and is reviewed at the MSK IRB by the expedited review process. This protocol is only for research that will be done on biospecimens obtained under identified protocols and their informed consent and research authorization that include the institutional future use questions. The consent and research authorization for the use of the biospecimens will be waived as per 45 CFR 46.116(d) and 45 CFR 164.512(i)(2)(ii).

11.0 TOXICITIES/SIDE EFFECTS

Patients who have received at least one dose of the NKTR-214 and nivolumab are evaluable for the safety assessment. Patients who discontinue the study before receiving their first dose of the study treatments will not be available for safety assessment and should be replaced.

11.1 Adverse Events

Definition of an AE
An adverse event (AE) is any untoward medical occurrence in a patient or clinical investigation patient, temporally associated with the use of a medicinal product, whether or not considered related to the medicinal product.

Note: An AE can therefore be any unfavorable and unintended sign (including an abnormal laboratory finding), symptom, or disease (new or exacerbated) temporally associated with the use of a medicinal product. For marketed medicinal products, this also includes failure to produce expected benefits, abuse, or misuse.

All AEs, either reported by the patient or observed by study staff, will be recorded for up to 30 days after end of treatment.

Toxicity will be assessed using the NCI CTCAE (version 4.03) and summarized by grade of severity, and relationship to each study drug (NKTR-214 and/or nivolumab)(http://ctep.cancer.gov/protocolDevelopment/electronic_applications/ctc.htm).

Examples of events meeting the definition of an AE include:
- Exacerbation of a chronic or intermittent pre-existing condition including either an increase in frequency and/or grade of the condition

- New conditions detected or diagnosed after study treatment administration even though it may have been present prior to the start of the study
- Signs, symptoms, or the clinical sequelae of a suspected interaction
- Signs, symptoms, or the clinical sequelae of a suspected overdose of either study treatment or a concomitant medication (overdose per se will not be reported as an AE/SAE).

Any abnormal laboratory test results (hematology, clinical chemistry, or urinalysis), or other safety assessments (e.g., ECGs, radiological scans, vital signs measurements) including those that worsen from baseline, and events felt to be clinically significant in the medical and scientific judgment of the investigator are to be recorded as an AE or SAE (see section 17 for SAE Reporting), in accordance with the protocol.

Events that do not meet the definition of an AE include:

- Medical or surgical procedure (e.g., endoscopy, appendectomy); the condition that leads to the procedure is an AE.
- Situations where an untoward medical occurrence did not occur (social and/or convenience admission to a hospital).
- Anticipated day-to-day fluctuations of pre-existing disease[1] or condition[1] present or detected at the start of the study that do not worsen.
- The disease/disorder being studied or expected progression, signs, or symptoms of the disease/disorder being studied, unless more severe than expected for the patient’s condition.

Any clinically significant safety assessments that are associated with the underlying disease, unless judged by the investigator to be more severe than expected for the patient's condition, are not to be reported as AEs or SAEs.

11.2 Dose Delay and Reduction Criteria

Patients whose treatment is interrupted or permanently discontinued due to an adverse event or clinically significant laboratory value must be followed until resolution or stabilization of the event.

A dose delay >8 weeks from the date of the planned dose (i.e., approximately 11 weeks since the previous dose) may require the patient to be discontinued from the study, with exception of patients who undergo surgery. A dose delay >8 weeks from the date of the planned dose (i.e., approximately 11 weeks since the previous dose) due to the occurrence of an adverse event that is considered related to NKTR-214 and/or nivolumab, the patient should be taken off the study treatment[1] that the adverse event is deemed to have been related to.

Tumor scan assessments for all patients should continue per protocol irrespective of dose delays.

Nivolumab Dose Delay Criteria

Dose reductions for nivolumab are not permitted in this study. Nivolumab may be delayed based on observed drug-related toxicities. If nivolumab is delayed, NKTR-214 administration can continue in the absence of NKTR-214-related toxicity that would warrant a dose delay.

Nivolumab administration should be delayed for the following:

- Any Grade ≥ 1 pneumonitis (in the case of Grade 1 pneumonitis, delay should be considered but is not required).
- Any Grade ≥ 2 non-skin, drug-related AE, except for fatigue and asymptomatic laboratory abnormalities that are corrected with supplementation/appropriate management within 72 hours of onset
- Grade 3/4 endocrinopathies that cannot be successfully treated with administration of replacement therapy.
- Any Grade ≥ 3 skin drug-related AE, except when occurring during the first 48 hours of NKTR-214 administration.
- Any Grade 3 drug-related laboratory abnormality with the following exceptions for lymphopenia, AST, ALT, or total bilirubin or asymptomatic amylase or lipase:
    - Grade 3 lymphopenia does not require a dose delay.
    - If a patient has a baseline AST, ALT, or total bilirubin that is within normal limits, delay dosing for drug-related Grade ≥ 2 toxicity.
    - If a patient has baseline AST, ALT, or total bilirubin within the Grade 1 toxicity range, delay dosing for drug-related Grade ≥ 3 toxicity.
    - Any Grade 3 drug-related amylase and/or lipase abnormality that is not associated with symptoms or clinical manifestations of pancreatitis does not require dose delay. The MSK PI should be consulted for such Grade ≥ 3 amylase or lipase abnormalities.
- Any Grade 4 amylase and/or lipase abnormalities regardless of symptoms or clinical manifestations. Patients can be restarted on therapy once the abnormalities have recovered to Grade 3 or less and after consultation with the MSK PI.
- Any AE, laboratory abnormality, or inter-current illness which, in the judgment of the Investigator, warrants delaying the dose of study medication.

NKTR-214 Dose Delay and Reduction Criteria

Dose delays and reductions are permitted for NKTR-214. Per recommendation of the Investigator and approval of the MSK PI, NKTR-214 may be delayed or reduced based on observed toxicities. If NKTR-214 is delayed, nivolumab administration can continue in the absence of nivolumab-related toxicity that would warrant a dose delay.

NKTR-214 may be delayed or reduced (only to NKTR-214 0.003mg/kg) for the following reasons:

- If dose reduction is required for NKTR-214, the dose of NKTR-214 will be 0.003mg/kg. There will be no further dose-reduction beyond this dose.
- Grade 1 or 2 toxicity: No requirement for dose delay or dose reduction. If the toxicity persists at Grade 2 following completion of Cycle 1, a dose delay (of either NKTR-214 or nivolumab) or dose reduction (of NKTR-214 to 0.003mg/kg) may be implemented at the discretion of the Investigator with the approval of the MSK PI.
- Grade 3 toxicity: NKTR-214 combination may be withheld if toxicity cannot be managed by adequate medical intervention. NKTR-214 combination dosing may resume at the same NKTR-214 dose or at a lower dose of NKTR-214 at 0.003mg/kg when toxicity resolves to Grade 1 or returns to baseline, except for instances where the potential recurrence of the event poses an undue risk for the patient.
- Grade 4 toxicity (excluding Grade 4 transient lymphopenia < 14 days in duration, and endocrine toxicities that cannot be successfully treated with administration of replacement therapy): dosing should be permanently discontinued.

11.3 Criteria to Resume NKTR-214 or Nivolumab

Patients will be permitted to resume therapy at the same dose level(s) following resolution of the AE to Grade ≤ 1 or to baseline within 11 weeks after the last dose, with the exception of patients who meet criteria for permanent discontinuation. Patients who meet criteria for permanent discontinuation must not receive further study therapy.

For both nivolumab and NKTR-214, if the decision is to resume study drug dosing, the patient may start upon resolution of adverse event as defined above. Patient's next cycle will be updated so that their consectutive visits will occur 21 days (+/- 3 days) after the date study drug was resumed.

Patients may resume treatment when the drug-related AE(s) resolve(s) to Grade ≤ 1 or baseline, with the following exceptions:

- Patients may resume treatment in the presence of Grade 2 fatigue.
- Patients with baseline Grade 1 AST/ALT or total bilirubin who require dose delays for reasons other than a 2-grade shift in AST/ALT or total bilirubin may resume treatment in the presence of Grade 2 AST/ALT **or** total bilirubin.
- Patients with combined Grade 2 AST/ALT **and** total bilirubin values meeting discontinuation parameters (section below) should have treatment permanently discontinued.
- Drug-related pulmonary toxicity, diarrhea, or colitis must have resolved to baseline before treatment is resumed. Patients with persistent Grade 1 pneumonitis after completion of a steroid taper over at least 1 month may be eligible for re-treatment if discussed with and approved by the MSK PI.
- Drug-related endocrinopathies adequately controlled with only physiologic hormone replacement may resume treatment after consultation with the MSK PI.
- Patients who delay study treatment due to any Grade ≥ 3 amylase or lipase abnormality that is not associated with symptoms or clinical manifestations of pancreatitis may resume nivolumab when the amylase or lipase abnormality has resolved to Grade < 3.
- Dose delay that results in treatment delay of > 11 weeks from the last dose requires treatment discontinuation, with exceptions as noted in section below. However, if the toxicity resolves to ≤ Grade 1 or baseline > 11 weeks after the last dose, but the patient does not otherwise meet the criteria for permanent discontinuation (see Section 11.4), and the Investigator believes that the patient is deriving clinical benefit, then the patient may be eligible to resume the study drug(s) following the approval of the MSK PI.

11.4 Permanent Treatment Discontinuation Criteria

Patients meeting any of the following criteria will be required to permanently discontinue all assigned study drug(s). However, with MSK PI approval, NKTR-214 treatment may continue if the toxicities listed below toxicities are considered related to nivolumab only.

- Progressive disease (see details regarding continuing treatment beyond initial assessment of progression per RECIST 1.1).
- Clinical deterioration, as assessed by the Investigator.
- Any Grade 2 drug-related uveitis, eye pain, or blurred vision that does not respond to topical therapy and does not improve to Grade 1 severity within 6 weeks

- Any Grade ≥ 2 drug-related pneumonitis or interstitial lung disease that does not resolve following dose delay and systemic steroids (also see Pulmonary Adverse Event Management Algorithm in **Appendix 3**).
- Any Grade 3 non-skin, drug-related AE lasting > 7 days, with the following exceptions for uveitis, pneumonitis, bronchospasm, diarrhea, colitis, neurologic toxicity, hypersensitivity reactions, infusion reactions, endocrinopathies, and laboratory abnormalities:
    - Grade 3 drug-related uveitis, pneumonitis, bronchospasm, diarrhea, colitis, neurologic toxicity, hypersensitivity reaction, or infusion reaction **of any duration** requires discontinuation.
    - Grade 3 drug-related endocrinopathies adequately controlled with only physiologic hormone replacement do not require discontinuation.
    - Grade 3 drug-related laboratory abnormalities do not require treatment discontinuation except:
    - Grade 3 drug-related thrombocytopenia > 7 days associated with clinically significant bleeding requires discontinuation.
    - Any drug-related liver function test abnormality that meets the following criteria require discontinuation (also see Hepatic Adverse Event Management Algorithm in **Appendix 3**):
        - AST or ALT > 5× to 10× ULN for > 2 weeks
        - AST or ALT > 10× ULN
        - Total bilirubin > 5× ULN
        - Concurrent AST or ALT > 3× ULN and total bilirubin > 2× ULN
- Any Grade 4 drug-related AE or laboratory abnormality, except for the following events, which do not require discontinuation:
    - Grade 4 neutropenia ≤ 7 days
    - Grade 4 lymphopenia or leukopenia
    - Isolated Grade 4 amylase or lipase abnormalities that are not associated with symptoms or clinical manifestations of pancreatitis and decrease to Grade < 4 after consultation with the MSK PI.
    - Isolated Grade 4 electrolyte imbalances/abnormalities that are not associated with clinical sequelae and are corrected with supplementation/appropriate management within 72 hours of their onset
    - Grade 4 drug-related endocrinopathy AEs such as adrenal insufficiency, adrenocorticotropic hormone deficiency, hyper- or hypothyroidism, or glucose intolerance, which resolve or are adequately controlled with physiologic hormone replacement (corticosteroids, thyroid hormones) or glucose-controlling agents, respectively, may not require discontinuation after discussion with and approval from the MSK PI.

- Any AE, laboratory abnormality, or intercurrent illness, which, in the judgment of the Investigator, presents a substantial clinical risk to the patient with continued treatment.
- Any dosing delay lasting > 11 weeks after the last dose, with the following exceptions:
  - Dosing delays to allow for prolonged steroid tapers to manage drug-related AEs are allowed. Prior to re-initiating treatment in a patient with a dosing delay lasting > 11 weeks after the last dose and with no more than 3 missed doses, the MSK PI must be consulted. Tumor assessments should continue as per protocol even if dosing is delayed.
  - Dosing delays > 6 weeks after the last dose, which occur for nondrug-related reasons, may be allowed if approved by the MSK PI. Prior to re-initiating treatment in a patient with a dosing delay lasting > 6 weeks after the last dose and with no more than 3 missed doses, the MSK PI must be consulted. Tumor assessments should continue as per protocol even if dosing is delayed, and patients must otherwise meet the criteria for continued treatment at the time re-initiation of study therapy is considered.

11.5 Management Algorithms for Immuno-Oncology Agents

Immuno-oncology agents are associated with AEs that can differ in severity and duration than AEs caused by other therapeutic classes. Nivolumab is considered an immuno-oncology agent in this protocol. Early recognition and management of AEs associated with immuno-oncology agents may mitigate severe toxicity. Management Algorithms have been developed to assist investigators in assessing and managing the following groups of AEs:

- Gastrointestinal
- Renal
- Pulmonary
- Hepatic
- Endocrinopathy
- Skin
- Neurological

11. 6 Treatment of NKTR-214 or Nivolumab-Related Infusion Reactions

Infusion reactions have been infrequently reported during the NKTR-214 infusions. Additionally, since nivolumab contains only human immunoglobulin protein sequences, it is unlikely to be immunogenic and induce infusion or hypersensitivity reactions. However, if such a reaction were to occur, with either NKTR-214 or nivolumab, it might manifest with fever, chills, rigors, headache, rash, pruritus,

arthralgias, hypotension, hypertension, bronchospasm, or other allergic-like reactions. All Grade 3 or 4 infusion reactions should be reported within 24 hours to the MSK PI and reported as an SAE if it meets the criteria. Infusion reactions should be graded according to National Cancer Institute Common Terminology Criteria for Adverse Events (NCI CTCAE) (Version 4.03) guidelines.

Treatment recommendations are provided below and may be modified based on local treatment standards and guidelines, as appropriate:

For **Grade 1** symptoms (mild reaction; infusion interruption not indicated; intervention not indicated):

- Remain at bedside and monitor patient until recovery from symptoms. The following prophylactic premedications are recommended for future infusions:
  - For patients ≥ 18 years:
    - diphenhydramine 50 mg (or equivalent)
    - acetaminophen/paracetamol 325 to 1000 mg at least 30 minutes before additional study drug administrations.
  - For patients < 18 years:
    - diphenhydramine 1mg/kg (50 mg max) acetaminophen/paracetamol 10 - 15 mg/kg (1000mg max) at least 30 minutes before additional study drug administrations.

For **Grade 2** symptoms (moderate reaction required therapy or infusion interruption but responds promptly to symptomatic treatment (e.g., antihistamines, non-steroidal anti-inflammatory drugs, narcotics, corticosteroids, bronchodilators, IV fluids); prophylactic medications indicated for 24 hours:

- For patients ≥ 18 years:
  - Stop the NKTR-214 or nivolumab infusion, begin an IV infusion of normal saline, and treat the patient with diphenhydramine 50 mg IV (or equivalent) and/or acetaminophen/paracetamol 325 to 1000 mg; remain at bedside and monitor patient until resolution of symptoms. Corticosteroid and/or bronchodilator therapy may also be administered as appropriate. If the infusion is interrupted, then restart the infusion at 50% of the original infusion rate when symptoms resolve; if no further complications ensue after 30 minutes, the rate may be increased to 100% of the original infusion rate.
  - For future infusions, the following prophylactic premedications are recommended: diphenhydramine 50 mg (or equivalent) and/or acetaminophen/paracetamol 325 to 1000 mg should be administered at least 30 minutes before nivolumab infusions. If necessary, corticosteroids (up to 25 mg of Solu-Cortef or equivalent) may be used.
- For patients < 18 years:

- Stop the NKTR-214 or nivolumab infusion, begin an IV infusion of normal saline, and treat the patient with diphenhydramine 1 mg/kg (50 mg max) IV or equivalent and/or acetaminophen/paracetamol 10 – 15 mg/kg mg (1000 mg max); remain at bedside and monitor patient until resolution of symptoms. Corticosteroid and/or bronchodilator therapy may also be administered as appropriate. If the infusion is interrupted, then restart the infusion at 50% of the original infusion rate when symptoms resolve; if no further complications ensue after 30 minutes, the rate may be increased to 100% of the original infusion rate.
- For future infusions, the following prophylactic premedications are recommended: diphenhydramine 1 mg/kg (50 mg max) or equivalent and/or acetaminophen/paracetamol 10 – 15 mg/kg (1000 mg max) should be administered at least 30 minutes before nivolumab infusions. If necessary, corticosteroids (1 mg/kg up to 25 mg of hydrocortisone or equivalent) may be used.

For **Grade 3 or Grade 4** symptoms (severe reaction, Grade 3: prolonged [i.e., not rapidly responsive to symptomatic medication and/or brief interruption of infusion]; recurrence of symptoms following initial improvement; hospitalization indicated for other clinical sequelae [e.g., renal impairment, pulmonary infiltrates]. Grade 4: Life-threatening; pressor or ventilatory support indicated):

- Immediately discontinue infusion of NKTR-214 or nivolumab.
- For patients ≥ 18 years:
    - Begin an IV infusion of normal saline and treat the patient as follows: Recommend bronchodilators, epinephrine 0.2 to 1 mg of a 1:1000 solution for subcutaneous administration or 0.1 to 0.25 mg of a 1:10,000 solution injected slowly for IV administration, and/or diphenhydramine 50 mg IV with methylprednisolone 100 mg IV (or equivalent), as needed. Patient should be monitored until the Investigator is comfortable that the symptoms will not recur. Nivolumab will be permanently discontinued. Investigators should follow their institutional guidelines for the treatment of anaphylaxis. Remain at bedside and monitor patient until recovery of the symptoms.

- For patients < 18 years:

- Begin an IV infusion of normal saline and treat the patient as follows: Recommend bronchodilators, epinephrine IM (Epi-Pen 0.3 mg for patients ≥ 30 kg and Epi-Pen Jr for patients < 30kg), and/or diphenhydramine 1 mg/kg up to 50 mg max IV with methylprednisolone 100 mg IV (or equivalent), as needed. Patient should be monitored until the Investigator is comfortable that the symptoms will not recur. Nivolumab will be permanently discontinued. Investigators should follow their institutional guidelines for the treatment of anaphylaxis. Remain at bedside and monitor patient until recovery of the symptoms.

In case of late-occurring hypersensitivity symptoms (e.g., appearance of a localized or generalized pruritus within 1 week after treatment), symptomatic treatment may be given (e.g., oral antihistamine or corticosteroids).

11.7 Prior and Concomitant Medications

Pre-medications should not be administered prior to the initial administration of NKTR-214 and nivolumab, but if a patient reports symptoms (such as nausea and/or vomiting), prophylactic use of anti-emetics may be used. As prophylaxis for flu-like symptoms in patients ≥ 18 years, 500 mg acetaminophen or 200 mg ibuprofen may be administered Days 2 through 5 after study drug administration. For patients < 18 years, 10 – 15mg/kg acetaminophen IV/PO (500 mg max) or 10mg/kg/dose ibuprofen (200 mg max) may be administered Days 2 through 5 after study drug administration.

All medications (prescription and over-the-counter [OTC]), vitamin and mineral supplements, and/or herbs taken by the patient from Screening through the End-of-Treatment visit will be documented and recorded, including start and stop date, dose and route of administration, frequency, and indication. Medications taken for a procedure (e.g., biopsy) should also be included.

11.8 Permitted Medications

Patients are permitted the use of topical, ocular, intra-articular, intranasal, and inhalational corticosteroids (with minimal systemic absorption). Adrenal replacement steroid doses > 10 mg daily prednisone are permitted for the first 4 days after administration of study drugs based on assessment of the degree of adrenal impairment and the extent of existing corticosteroid supplementation. A brief (less than 3 weeks) course of corticosteroids for prophylaxis (e.g., contrast dye

allergy) or for treatment of non-autoimmune conditions (e.g., delayed-type hypersensitivity reaction caused by a contact allergen) is permitted.

Concomitant palliative and supportive care for disease related symptoms (including bisphosphonates and RANK-L inhibitors) is allowed if initiated prior to first dose of study therapy. Prior palliative radiotherapy must have been completed at least 2 weeks prior to 2 weeks prior to enrollment.

Prohibited Medications

- Immunosuppressive agents
- Immunosuppressive doses of systemic corticosteroids (exceptions in section 11.7)
- Any antineoplastic therapy (i.e., chemotherapy, hormonal therapy, immunotherapy, extensive non-palliative radiation therapy, investigational agent, or radiation therapy) is prohibited during the study. Palliative radiation is permitted to ≤ 2 non-target lesions at a time, provided it is completed 14 days before dosing of NKTR-214 and nivolumab.

Consideration should be given to discontinuing antihypertensive medications including diuretics, as well as other drugs with hypotensive properties (e.g., alpha blockers for BPH), prior to each dose of NKTR-214, particularly when therapy involves multiple anti-hypertensive drugs and classes other than thiazide diuretics.

Antihypertensive medications should be discontinued no less than 12 hours and no more than 48 hours prior to each dose of NKTR-214. Antihypertensive medications may be reinstituted in between doses of NKTR-214 if the diastolic pressure exceeds 90 mmHg and/or the systolic pressure exceeds 160 mmHg.
In addition, prohibited medications listed in the current nivolumab prescribing information are not allowed.

11.9 Pregnancy Risk

Women of childbearing potential and male patients with women of childbearing potential partners must be willing to use an adequate method of contraception.

Women of childbearing potential must agree to use highly effective methods of birth control (defined as those, alone or in combination, that result in a low failure rate [i.e., < 1% per year] when used consistently and correctly, such as oral contraceptives, surgical sterilization, an intrauterine device, and/or 2 barrier methods [e.g., condom and cervical barrier such as a diaphragm]). Protections against pregnancy must be continued for at least 7 months (male participants with WOCBP partners) or 5 months (female participants of child bearing potential) after the last dose of study drugs.

12.0 CRITERIA FOR THERAPEUTIC RESPONSE/OUTCOME ASSESSMENT

12.1 Antitumor effect

All patients will undergo a baseline staging CT scan of the chest (with or without contrast), abdomen and pelvis (with or without contrast), and MRI of the affected area if deemed necessary by the treating physician. Response evaluations will occur at week 8 and every 8 weeks subsequently (± 1 week window) until week 56 and then every 12 weeks thereafter or at discretion of the treating investigator.

Response and progression will be evaluated in this study using the international criteria proposed by the revised Response Evaluation Criteria in Solid Tumors (RECIST) guideline, version 1.1 (Primary response criteria).[36] Changes in the largest diameter (unidimensional measurement) of the tumor lesions and the shortest diameter in the case of malignant lymph nodes are used in the RECIST criteria.

In addition, patients will also be evaluated using the irRECIST which *will not be used* for treatment decisions (Secondary Response Criteria) and are discussed separately in section 12.12

In patients who have initial evidence of progressive disease (PD) as per RECIST criteria or irRECIST criteria, it is at the discretion of the investigator to continue a patient on study treatment until confirmation of PD ≥ 4 weeks. This clinical judgment decision should be based on the patient's overall clinical condition, including performance status, clinical symptoms, and laboratory data.

Patients will receive treatment if they are clinically stable as defined by the following criteria:

- Absence of signs and symptoms indicating PD.
- No decline in the Eastern Cooperative Oncology Group (ECOG) performance status
- Absence of rapid progression of disease
- Absence of progressive tumor at critical anatomical sites (e.g., cord compression) requiring urgent alternative medical intervention

12.2 Definitions

Evaluable for toxicity – All patients will be evaluable for toxicity from the time of their first treatment with NKTR-214 and/or nivolumab

Evaluable for efficacy – Only those patients who have a measurable disease based on RECIST 1.1 criteria will be eligible for this study, as highlighted in Section 6.0.

These patients will have their response classified according to the definitions stated below.

Only those patients who have measurable disease present at baseline, have received at least one cycle of therapy, and have had their disease re-evaluated will be considered evaluable for response. (Note: Patients who exhibit objective disease progression prior to the end of cycle 1 will also be considered evaluable and determined to be non-responders. Note: one cycle of therapy consists of 21 days where the study therapy (NKTR-214 and nivolumab) is administered at the start of the cycle on day 1)

12.2.1 Disease Parameters

Measurable disease – Measurable lesions are defined as those that can be accurately measured in at least one dimension (longest diameter to be recorded for non-nodal lesions and short axis for nodal lesions) as >20mm by chest x-ray, as >10mm with CT scan or MRI, or >10mm with calipers by clinical exam. All tumor measurements must be recorded in millimeters (or decimal fractions of centimeters).

To be considered pathologically enlarged and measurable, a lymph node must be > 15 mm in short axis when assessed by CT scan (CT scan slice thickness recommended to be no greater than 5 mm). At baseline and in follow-up, only the short axis will be measured and followed.

Note: Tumor lesions that are situated in a previously irradiated area are not considered measurable unless there is evidence of progression after radiation therapy.

NOTE for irRECIST: During target lesion selection the radiologist will consider information on the anatomical sites of previous intervention (e.g. previous irradiation, RF-ablation, TACE, surgery, etc.). Lesions undergoing prior intervention will not be selected as target lesions unless there has been a demonstration of progress in the lesion.

Non-measurable disease – All other lesions (or sites of disease), including small lesions (longest diameter <10mm or pathological lymph nodes with ≥10 to <15mm short axis) are considered non-measurable disease. Bone lesions, leptomeningeal disease, ascites, pleural/pericardial effusions, lymphangitis cutis/pulmonitis, inflammatory breast disease and abdominal masses (not followed by CT or MRI), are considered as non-measurable.

NOTE for irRECIST: Lesions that are partially cystic or necrotic can be selected as target lesions. The longest diameter of such a lesion will be added to the Total Measured Tumor Burden (TMTB) of all target lesions at baseline. If other lesions

with a non-liquid/non-necrotic component are present, those should be preferred. Brain lesions detected on brain scans can be considered as both target or non-target lesions depending on the protocol definition.

Target lesions – All measurable lesions up to a maximum of 2 lesions per organ and 5 lesions in total, representative of all involved organs, should be identified as target lesions and recorded and measured at baseline. Target lesions should be selected on the basis of their size (lesions with the longest diameter), be representative of all involved organs, but in addition should be those that lend themselves to reproducible repeated measurements. A sum of the diameters (longest for non-nodal lesions, short axis for nodal lesions) for all target lesions will be calculated and reported as the baseline sum diameters. The baseline sum diameters will be used as a reference to further characterize any objective regression in the measurable dimension of the disease.
Non-target lesions – All other lesions (or sites of disease) including any measurable lesions over and above the 5 target lesions should be identified as non-target lesions and should also be recorded at baseline. Measurements of these lesions are not required, but the presence, absence or in rare cases unequivocal progression of each should be noted throughout follow-up.

12.3 Methods for Evaluation of Measurable Disease

All measurements should be taken and recorded in metric notation using a ruler or calipers. All baseline evaluations should be performed as closely as possible to the beginning of treatment and never more than 4 weeks before the beginning of the treatment.

The same method of assessment and the same technique should be used to characterize each identified and reported lesion at baseline and during follow-up portion of study. Imaging-based evaluation is preferred to evaluation by clinical examination unless the lesion[1] being followed cannot be imaged but are assessable by clinical exam.

Clinical lesions - Clinical lesions will only be considered measurable when they are superficial (e.g., skin nodules and palpable lymph nodes) and ≥10 mm diameter as assessed using calipers (e.g., skin nodules). In the case of skin lesions, documentation by color photography, including a ruler to estimate the size of the lesion, is recommended.

Chest x-ray - Lesions on chest x-ray are acceptable as measurable lesions when they are clearly defined and surrounded by aerated lung. However, CT is preferable.

Conventional CT and MRI - This guideline has defined measurability of lesions on CT scan based on the assumption that CT slice thickness is 5 mm or less. If CT

scans have slice thickness greater than 5 mm, the minimum size for a measurable lesion should be twice the slice thickness. MRI is also acceptable in certain situations (e.g. for body scans).

Use of MRI remains a complex issue. MRI has excellent contrast, spatial, and temporal resolution; however, there are many image acquisition variables involved in MRI, which greatly impact image quality, lesion conspicuity, and measurement. Furthermore, the availability of MRI is variable globally. As with CT, if an MRI is performed, the technical specifications of the scanning sequences used should be optimized for the evaluation of the type and site of disease. Furthermore, as with CT, the modality used at follow-up should be the same as was used at baseline and the lesions should be measured/assessed on the same pulse sequence. It is beyond the scope of the RECIST guidelines to prescribe specific MRI pulse sequence parameters for all scanners, body parts, and diseases. Ideally, the same type of scanner should be used and the image acquisition protocol should be followed as closely as possible to prior scans. Body scans should be performed with breath-hold scanning techniques, if possible.

12.3.1 Primary Response Criteria (RECIST 1.1)

12.3.1.1 Evaluation of Target Lesions

Complete Response (CR): Disappearance of all target lesions. Any pathological lymph nodes (whether target or non-target) must have reduction in short axis to <10 mm.

Partial Response (PR): At least a 30% decrease in the sum of the diameters of target lesions, taking as reference the baseline sum diameters.

Progressive Disease (PD): At least a 20% increase in the sum of the diameters of target lesions, taking as reference the smallest sum on study (this includes the baseline sum if that is the smallest on study). In addition to the relative increase of 20%, the sum must also demonstrate an absolute increase of at least 5 mm. (Note: the appearance of one or more new lesions is also considered progressions).

Stable Disease [1]: Neither sufficient shrinkage to qualify for PR nor sufficient increase to qualify for PD, taking as reference the smallest sum diameters while on study.

12.3.1.2 Evaluation of Non-Target Lesions

Complete Response (CR): Disappearance of all non-target lesions and normalization of tumor marker level. All lymph nodes must be non-pathological in size (<10 mm short axis).

Non-CR/Non-PD: Persistence of one or more non-target lesion[1].

Progressive Disease (PD): Appearance of one or more new lesions and/or unequivocal progression of existing non-target lesions. Unequivocal progression should not normally trump target lesion status. It must be representative of overall disease status change, not a single lesion increase.

Although a clear progression of "non-target" lesions only is exceptional, the opinion of the treating physician should prevail in such circumstances, and the progression status should be confirmed at a later time by the review panel (or MSKCC Principal Investigator).

12.3.1.3 Evaluation of Best Response

The best response is the best response recorded from the start of the treatment until disease progression/recurrence (taking as reference for progressive disease the smallest measurements recorded since the treatment started). The patient's best response assignment will depend on the achievement of both measurement and confirmation criteria.

Table 13. Evaluation of Best Response – RECIST criteria

<table>
<tr><th>Target Lesions</th><th>Non-Target Lesions</th><th>New Lesions</th><th>Best Response</th><th>Best Response for This Category also Requires:</th></tr>
<tr><td>CR</td><td>CR</td><td>No</td><td>CR</td><td>≥ 4 wks. Confirmation</td></tr>
<tr><td>CR</td><td>Non-CR/ Non-PD</td><td>No</td><td>PR</td><td rowspan="2">≥ 4 wks. Confirmation</td></tr>
<tr><td>PR</td><td>Non-CR/ Non-PD</td><td>No</td><td>PR</td></tr>
<tr><td>SD</td><td>Non-CR/ Non-PD</td><td>No</td><td>SD</td><td>documented at least once ≥ 4 wks. from baseline</td></tr>
<tr><td>PD</td><td>Any</td><td>Yes or No</td><td>PD</td><td rowspan="3">no prior SD, PR or CR</td></tr>
<tr><td>Any</td><td>PD*</td><td>Yes or No</td><td>PD</td></tr>
<tr><td>Any</td><td>Any</td><td>Yes</td><td>PD</td></tr>
<tr><td colspan="5">* In exceptional circumstances, unequivocal progression in non-target lesions may be accepted as disease progression.

<u>Note</u>: Patients with a global deterioration of health status requiring discontinuation of treatment without objective evidence of disease progression at that time should be reported as “symptomatic deterioration.” Every effort should be made to document the objective progression even after discontinuation of treatment.</td></tr>
</table>

12.4 Confirmation of response

Complete or partial response may only be claimed if the criteria for each are met at a subsequent time point (≥4 weeks later) in studies with a primary endpoint that include response rate.

12.4.1 Special note on target lesions that become “too small to measure”

While on study, all lesions (nodal and non-nodal) recorded at baseline should have their actual measurements recorded at each subsequent evaluation, even when very small (e.g. 2mm). However, sometimes, lesions or lymph nodes which are recorded as target lesions at baseline become so faint on CT scan that the radiologist may not feel comfortable assigning an exact measure and may report them as being “too small to measure”. When this occurs, it is important that a value be recorded on the D2M form. If it is the opinion of the radiologist that the lesion has likely disappeared, the measurement should be recorded as 0 mm. If the lesion is believed to be present and is faintly seen but too small to measure, a default value of 5 mm should be assigned.

12.5 Not evaluable (NE)

When no imaging/measurement is done at all at a particular time point, the patient is considered not evaluable (NE) at that time point.

12.6 Early death

If the patient has no repeat tumor assessments following initiation of study therapy resulting from the death of the patient due to disease or treatment, it is considered early death.

12.7 Symptomatic Deterioration

Patients with global deterioration of health status requiring discontinuation of treatment without objective evidence of disease progression at that time be recorded as "symptomatic deterioration". Every effort should be made to document objective progression even after discontinuation of treatment.

12.8 Duration of response

Defined as the time measurement criteria are first met for CR/PR until the first date that recurrent or progressive disease is objectively documented (taking as reference for progressive disease the smallest measurement recorded on study).

12.9 Duration of stable disease

Measured from the start of the treatment until the criteria for progression are met, taking as reference the smallest sum on study (if the baseline sum is the smallest, this is the reference for calculation of PD).

12.10 Progression-Free Survival (PFS)

PFS is defined as the period from start of study treatment until recurrent or progressive of disease (POD) is objectively documented (taking as reference for progressive disease the smallest measurement recorded on study), death, or date of last contact.

12.11 Overall Survival (OS)

OS is defined as the observed length of life from start of study treatment to death or the date of last contact.

12.12 Secondary Response Criteria – Immune related RECIST (irRECIST)

In 2013, Nishino et al. demonstrated that immune-related response criteria using unidimensional measurements were highly concordant with the bidimensional results of irRC, but with less measurement variability. Based on these findings and in order to utilize both the established criteria of irRC and RECIST 1.1, the two systems have been adapted, modified, and combined into the Immune-related Response Evaluation Criteria in Solid Tumors (irRECIST). The adapted irRECIST

criteria are modifications to the irRC, incorporating the findings of Nishino et al. and the advantages of RECIST 1.1 while overcoming the shortcomings of each of the other guidelines.

irRECIST
Immune-related RECIST (irRECIST) guidelines according to Bohnsack et al. are presented below.

<u>I. Baseline Assessments in irRECIST</u>

In irRECIST, baseline assessment and measurement of measurable/non-measurable and target/non-target lesions and lymph nodes are in line with RECIST 1.1.

One new definition is added: If a patient has no measurable and no non-measurable disease at baseline the radiologist will assign 'No Disease' (irND) as the overall tumor assessment for any available follow-up time points unless new measurable lesions are identified and contribute to the total measured tumor burden (TMTB). irND is a valid assessment in studies with adjuvant setting where the protocol and study design allow the inclusion of patients with no visible disease

Follow-up Assessments in irRECIST

A. Follow-up recording of target and new measurable lesions

The key difference in irRECIST is that the appearance of new lesions does not automatically indicate progression. Instead, all measured lesions (baseline-selected target lesions and new measurable lesions) are combined into the total measured tumor burden (TMTB) at follow up. Baseline-selected target lesions and new measurable lesions are NOT assessed separately. Measurements of those lesions are combined into the TMTB, and one combined assessment provided.

In order to be selected as new measurable lesions (≤ 2 lesions per organ, ≤ 5 lesions total, per time point), new lesions must meet criteria as defined for baseline target lesion selection and meet the same minimum size requirements of 10 mm in long diameter and minimum 15 mm in short axis for new measurable lymph nodes. New measurable lesions should be prioritized according to size, and the largest lesions elected as new measured lesions.

B. Follow-up non-target assessment

RECIST 1.1 definitions for assessment of non-target lesions apply. The response of non-target lesions primarily contributes to the overall response assessments of irCR

and irNon-CR/Non-PD (irNN). Non-target lesions do not affect irPR and irSD assessments. Only a massive and unequivocal worsening of non-target lesions alone, even without progress in the TMTB is indicative of irPD. In alignment with RECIST 1.1, baseline selected non-target lesions can never convert to measurable lesions, not even if they increase in size at subsequent time points and become measurable. Only true new lesions can be measured and contribute to the TMTB.

C. Follow-up for New Non-Measurable Lesions

All new lesions not selected as new measurable lesions are considered new non-measurable lesions and are followed qualitatively. Only a massive and unequivocal progression of new non-measurable lesions leads to an overall assessment of irPD for the time point. Persisting new non-measurable lesions prevent irCR.

<u>Overall Assessments for irRECIST</u>

The irRECIST overall tumor assessment is based on TMTB of measured target and new lesions, non-target lesion assessment and new non-measurable lesions.

At baseline, the sum of the longest diameters (SumD) of all target lesions (up to 2 lesions per organ, up to total 5 lesions) is measured. At each subsequent tumor assessment (TA), the SumD At each subsequent tumor assessment (TA), the SumD of the target lesions and of new, measurable lesions (up to 2 new lesions per organ, total 5 new lesions) are added together to provide the total measurable tumor burden (TMTB).

**Overall Assessments by irRECIST**	
**Complete Response (irCR)**	Complete disappearance of all measurable and non-measurable lesions. Lymph nodes must decrease to < 10 mm in short axis.
**Partial Response (irPR)**	Decrease of ≥ 30% in TMTB relative to baseline, non-target lesions are irNN, and no unequivocal progression of new non-measurable lesions<br>• If new measurable lesions appear in patients with **no target lesions at baseline**, irPD will be assessed. That irPD time point will be considered a new baseline, and all subsequent time points will be compared to it for response assessment. irPR is possible if the TMTB of new measurable lesions decreases by ≥ 30% compared to the first irPD documentation

	• irRECIST can be used in the **adjuvant setting**, in patients with no visible disease on CT/MRI scans. The appearance of new measurable lesion(s) automatically leads to an increase in TMTB by 100% and leads to irPD. These patients can achieve a response if the TMTB decreases at follow-up, as a sign of delayed response.<br>• Based on the above, MSK may consider enrolling patients with no measurable disease and/or no visible disease in studies with response related endpoints.
**Stable Disease (irSD)**	Failure to meet criteria for irCR or irPR in the absence of irPD
**Progressive Disease (irPD)**	Minimum 20% increase and minimum 5 mm absolute increase in TMTB compared to nadir, or irPD for non-target or new non-measurable lesions. Confirmation of progression is recommended minimum 4 weeks after the first irPD assessment. An irPD confirmation scan may be recommended for patients with a minimal TMTB %-increase over 20% and especially during the flare time-window of the first 12 weeks of treatment, depending on the compound efficacy expectations, to account for expected delayed response.<br>• In irRECIST a substantial and unequivocal increase of **non-target lesions** is indicative of progression.<br>• IrPD may be assigned for a patient with multiple **new non-measurable lesions** if they are considered to be a sign of unequivocal massive worsening
**Other**	**irNE:** used in exceptional cases where insufficient data exist.<br>**irND:** in adjuvant setting when no disease is detected<br>**irNN:** no target disease was identified at baseline, and at follow-up the patient fails to meet criteria for irCR or irPD

13.0 CRITERIA FOR REMOVAL FROM STUDY

Patients will be removed from the study when any of the criteria listed below applies. The reason for removal from the study and the date the patient was removed must be documented in the Clinical Research Database system. Patients who come off study before their first radiological assessment from baseline for reasons other than disease progression or treatment related toxicities may be replaced.
All patients who have received at least one dose of both study treatments will be assessable for safety of the combination therapy. Patients evaluable for efficacy analysis will include all patients treated with NKTR-214 and nivolumab and assessable for response. Patients who are missing an assessment of response post baseline will not be considered evaluable for response unless they missed the assessment due to progression of disease or treatment related toxicities in which case they will be considered non-responders for that time point.

In the absence of treatment delays due to adverse events, treatment with NKTR-214 and nivolumab may continue until one of the following criteria applies:

- Intercurrent illness that prevents further administration of treatment
- Intolerance of study treatment
- Patient decides to withdraw from the study
- Physician decides to withdraw a patient from the study for a reason not listed here
- Pregnancy in patient
- The patient is lost to follow-up
- Inability of the patient to comply with the requirement of the protocol for treatment or evaluation.
- End of study, whichever occurs first.

Due to the mechanism of action, patients may experience growth in existing tumors or the appearance of new tumors prior to maximal clinical benefit of NKTR-214 and nivolumab. The patient may be allowed to continue study treatment after initial RECIST 1.1 defined progression if they are assessed by the treating physician to be deriving clinical benefit and tolerating study treatment. The treating physician may consult with the MSK PI for help with assessing the patient. Such patients should discontinue study therapy upon further evidence of progression at the discretion of the treating investigator.

Safety follows up and End of Study visit
Upon permanent discontinuation from the study treatment for any reason, patients will be seen for their end of treatment visit as outlined within table 3.

Patients whose treatment is interrupted or permanently discontinued due to an adverse event or clinically significant laboratory value must be followed until resolution or stabilization of the event.

Reporting of adverse events will occur for up to 30 days for serious adverse events (within 30 days after the last administration of NKTR-214 and nivolumab, whichever is later), Events of Clinical Interest (within 30 days after the last administration of NKTR-214 and nivolumab, whichever is later), and documentation of concomitant medications.

Long-Term Follow-up Procedures:
All patients who permanently discontinue study drug for any reason other than withdrawal of full consent will be contacted by clinic visit or telephone to assess survival and initiation of additional sarcoma therapy. Contact for all patients will be attempted every 12 weeks (±28 days) following the safety follow-up visit until death, patient withdraws full consent, or up to 12 months after the end of treatment.

14.0 BIOSTATISTICS

A sample size of 10 patients is planned for seven of the histological cohorts. If 2 or more confirmed responses are observed among the 10 patients in an arm, the drug combination will be claimed to be positive and worthy further investigation for that arm. A sample size of 15 is planned for each of the other two cohorts (in order to account for the greater sample heterogeneity in these cohorts). If 3 or more confirmed responses are observed among the 15 patients in each of these two arms, the drug combination will be claimed to be positive and worthy further investigation for that arm. The probability of observing a positive result for an individual arm and the probability of observing at least one positive arm among the six arms are provided in the below for a range of true response rate in an arm.

	**Response.rate**	**p0.one.arm (n=10)**	**p0.one.arm (n=15)**	**p0.overall**
1	0.05	0.09	0.04	0.52
2	0.10	0.26	0.18	0.92
3	0.15	0.46	0.40	1.00
4	0.20	0.62	0.60	1.00
5	0.25	0.76	0.76	1.00
6	0.30	0.85	0.87	1.00
7	0.35	0.91	0.94	1.00

With the small sample size planned for the pilot study, we acknowledge that there will be a 52% probability that at least one cohort will look promising with respect to ORR even if all cohorts have a true overall response rate of 5%. We will interpret the study results cautiously when planning future studies and may follow up promising findings with a larger phase 2 study in the future.

An early stopping rule for excessive drug-related toxicity that are grade 3 or higher will be implemented across the six study arms with continuous monitoring. Assume an acceptable toxicity rate of 30%, and an unacceptable toxicity rate of 55%. The study will be stopped early if observing grade 3 or higher drug-related toxicity in at least 7/10, 11/20, 14/30, 18/40, 22/50, 25/60, 29/70, 32/80, 36/90 and 39/100 patients. The probability of stopping under this rule is 7% if the true drug-related toxicity event rate is 30%, and is 97% if the true drug-related toxicity event rate is 55%. Of note, combinatorial immunotherapy with agents such as nivolumab and ipilimumab have demonstrated grade 3/4 drug related toxicity of 54% [38]. Therefore, 55% appears to be a reasonable unacceptable rate for designing the early stopping rule.

The patient population in this study represents patients with locally advanced/metastatic sarcoma that have received at least one line of previous therapy. The references supporting the 5% null rate include the PALETTE, double-blind, randomized placebo-controlled trial[39] and a study looking at the efficacy of eribulin in patients with advanced sarcoma. The PALETTE study compared pazopanib with placebo in patients with least one regimen containing an anthracycline and a maximum of four previous lines of systemic therapy for metastatic disease (no more than two lines of combination regimens). The response rate for pazopanib was 6% in this study. Pazopanib was granted FDA approval for advanced sarcoma patients that have previously received chemotherapy on the basis of this study which showed an improvement in PFS favoring pazopanib. The second reference refers to a randomized phase 3 study that compared eribulin with dacarbazine in patients with advanced leiomyosarcoma and liposarcoma that have progressed on two previous lines of therapy including an anthracycline. In this study, the proportion of patients who had an objective response did not differ between treatment groups (nine [4%] in the eribulin group *vs* 11 [5%] in the dacarbazine group; p=0.62). Dacarbazine is FDA approved for advanced sarcoma and eribulin obtained FDA approval for use in advanced liposarcoma on the basis of this study. Hence, a 5% response rate has been seen with standard chemotherapy agents approved in this patient population. Therefore, in assessing the efficacy of a new therapy in this study population a response rate of > 5% would be preferred to determine the therapy as having a signal of efficacy.

Secondary endpoints (to be conducted separately for each cohort) include:

1. <u>Safety</u>
   Adverse events will be graded according to the NCI CTCAE v4.03, presented on an individual basis and summarized using descriptive statistics.

2. <u>Best Objective Response Rate by irRECIST</u>

Best objective response rate (complete response + partial response) by 24 weeks by irRECISTof NKTR-214 in combination with nivolumab in patients with metastatic and/or locally advanced, high grade sarcoma.

3. Survival
   Progression free survival (PFS) rate at 24 weeks, median PFS, per RECIST 1.1, overall survival (OS) at 12 months and median OS. OS and progression-free survival intervals will be estimated using Kaplan-Meier methodology

Descriptive statistics will be provided for demographics, safety, efficacy, and biomarkers as appropriate. Due to the small sample size, the correlative studies using tumor biopsies will be exploratory and hypothesis generating in nature.

Patients who have not experienced the event of interest by the end of the study will be censored at the time of the last follow-up.

Patients evaluable for efficacy analysis will include all patients treated with NKTR-214 and/or nivolumab and assessable for response. Patients who are missing an assessment of response post baseline will not be considered evaluable for response unless they missed the assessment due to progression of disease or treatment related toxicities in which case they will be considered non-responders for that time point. Patients who miss an assessment of response post baseline for any reason other than progression of disease and treatment related toxicity will be replaced.

Patients evaluable for safety analysis will include all patients who have received at least one dose of NKTR-214 and nivolumab.

We expect to accrue approximately 1-2 patients each month at each participating institution (MSK and MD Anderson) and hence expect to finish enrollment in 15-30 months.

14.2 Correlative Endpoints Statistical Considerations

The study is not powered to detect specific hypotheses; rather this data and analysis will help better identify patients having the potential to benefit from this therapy and aid in designing larger Phase II and III studies.

I. To assess the potential effect of NKTR-214 and nivolumab on selected biomarker expression measured in pre- and post-treatment tumor tissue and the association between these biomarkers and with clinical outcome, including level of PD-1/PD-L1 expression, presence of tumor infiltrating lymphocytes (TILs) and tumor antigens, gene expression profile, and the T-cell receptor clonality in tumor-infiltrating lymphocytes (TIL).

a. The pre- and post-treatment measurements will be compared using the paired t-test for each of these biomarkers. The associations with the selected biomarkers, in terms of both the pre-treatment measurement and the difference between pre- and post-treatment measurements, will be evaluated using the two-sample t-test for clinical outcome such as response and clinical benefit, if there are a reasonable number of events for each outcome.

II. To evaluate associations between selected biomarkers measured in serial peripheral blood over time with clinical efficacy, including immunophenotyping and functional analyses, evaluation of serum levels of chemokines, cytokines and other immune mediators, and characterization of T-cell receptor clonality in peripheral blood.

a. Summary statistics will be used to for describing changes across time. In addition the time course of biomarker measurements will be investigated graphically, by summary plots or individual patient plots; their trends over time will be categorized either by visual inspection (if there are clear trend groups such as monotonically increasing or monotonically decreasing) or by pattern recognition methods such as K-means clustering.. The associations with the observed trend in selected biomarkers analyzed will be evaluated using categorical data analysis (including Fisher's exact test) for clinical outcome such as response and clinical benefit, if there are a reasonable number of events for each outcome.

III. To evaluate the association between baseline tumor mutational burden and neoantigen production with clinical efficacy of the study therapy.

a. The associations with baseline tumor mutational burden and neoantigen production will be evaluated using categorical data analysis (including Fisher's exact test) for clinical outcome such as response and clinical benefit, if there are a reasonable number of events for each outcome.

15.0 RESEARCH PARTICIPANT REGISTRATION AND RANDOMIZATION PROCEDURES

15.1 Research Participant Registration

Confirm eligibility as defined in the section entitled Inclusion/Exclusion Criteria. Obtain informed consent, by following procedures defined in section entitled Informed Consent Procedures. During the registration process registering individuals will be required to complete a protocol specific Eligibility Checklist. The individual signing the Eligibility Checklist is confirming whether or not the participant is eligible to enroll in the study.

Study staff are responsible for ensuring that all institutional requirements necessary to enroll a participant to the study have been completed. See related MSK Clinical Research Policy and Procedure #401 (Protocol Participant Registration).

15.2 Randomization

N/A

16.0 DATA MANAGEMENT ISSUES

A MSK Clinical Research Coordinator (CRC) will be assigned to the study. The responsibilities of the CRC include project compliance, data collection, abstraction and entry, data reporting, regulatory monitoring, problem resolution and prioritization, and coordinate the activities of the protocol study team.

The data collected for this study will be entered into a secure internet based system, Medidata Rave. Source documentation will be available to support the computerized patient record. MSK will be the data coordinating center under the guidance of the Multicenter Protocol Executive Committee (MPEC). MSK will be responsible for reporting to the funding source (as applicable) and governing agencies.

16.1 Quality Assurance

Regular registration reports will be generated to monitor patient accruals and completeness of registration data. Routine data quality reports will be generated to assess missing data and inconsistencies. Accrual rates and extent and accuracy of evaluations and follow-up will be monitored periodically throughout the study period and potential problems will be brought to the attention of the study team for discussion and action.

Random-sample data quality and protocol compliance audits will be conducted by the study team, as an ongoing review process.

16.2 Data and Safety Monitoring

The Data and Safety Monitoring (DSM) Plans at Memorial Sloan-Kettering Cancer Center were approved by the National Cancer Institute in September 2001. The plans address the new policies set forth by the NCI in the document entitled “Policy of the National Cancer Institute for Data and Safety Monitoring of Clinical Trials” which can be found at: http://cancertrials.nci.nih.gov/researchers/dsm/index.html. The DSM Plans at MSK were established and are monitored by the Clinical Research Administration. The MSK Data and Safety Monitoring Plans can be found on the MSK Intranet at: http://mskweb5.mskcc.org/intranet/html/99074.cfm

There are several different mechanisms by which clinical trials are monitored for data, safety and quality. There are institutional processes in place for quality assurance (e.g., protocol monitoring, compliance and data verification audits, therapeutic response, and staff education on clinical research QA) and departmental procedures for quality control, plus there are two institutional committees that are responsible for monitoring the activities of our clinical trials programs. The committees: *Data and Safety Monitoring Committee (DSMC)* for Phase I and II clinical trials, and the *Data and Safety Monitoring Board (DSMB)* for Phase III clinical trials, report to the Center's Research Council and Institutional Review Board.

During the protocol development and review process, each protocol will be assessed for its level of risk and degree of monitoring required. Every type of protocol (e.g., NIH sponsored, in-house sponsored, industrial sponsored, NCI cooperative group, etc.) will be addressed and the monitoring procedures will be established at the time of protocol activation.

The MSK DSMC will monitor safety and data quality across all participating institutions (MSK and external sites). Regular study teleconferences with all participating institutions will be held to discuss SAEs or other toxicities and study progress.

17.0 PROTECTION OF HUMAN SUBJECTS

Every effort will be made to maintain patient confidentiality. All the data will be confidential, maintained in a password protected electronic database and will comply with all HIPAA guidelines. Research and hospital records are confidential. Patient's name or any other personally identifying information will not be used in reports or publications resulting from this study. The Food and Drug Administration or other authorized agencies (e.g., qualified monitors) may review patients' records and pathology slides, as required.

17.1 Privacy

MSK's Privacy Office may allow the use and disclosure of protected health information pursuant to a completed and signed Research Authorization form. The use and disclosure of protected health information will be limited to the individuals described in the Research Authorization form. A Research Authorization form must be completed by the Principal Investigator and approved by the IRB and Privacy Board (IRB/PB).

17.2 Serious Adverse Event (SAE) Reporting

An adverse event is considered serious if it results in ANY of the following outcomes:

- Death
- A life-threatening adverse event
- An adverse event that results in inpatient hospitalization or prolongation of existing hospitalization
- A persistent or significant incapacity or substantial disruption of the ability to conduct normal life functions
- A congenital anomaly/birth defect
- Important Medical Events (IME) that may not result in death, be life threatening, or require hospitalization may be considered serious when, based upon medical judgment, they may jeopardize the patient or subject and may require medical or surgical intervention to prevent one of the outcomes listed in this definition
- All grade 4 laboratory abnormalities

Note: Hospital admission for a planned procedure/disease treatment is not considered an SAE.

SAE reporting is required as soon as the participant signs consent. Prior to starting investigational treatment, SAE reporting is only required if the event is related to any protocol test or procedure. SAE reporting is required for 30-days after the participant's

last investigational treatment or intervention. Any events that occur after the 30-day period and that are at least possibly related to protocol treatment must be reported.

Note: Reported SAEs attributed to nivolumab are required to be followed for 100 days after the participants last investigational treatment.

For Participating Institutions, refer to Appendix 1 for SAE reporting instructions.

For MSK patients:

If an SAE requires submission to the IRB office per IRB SOP RR-408 'Reporting of Serious Adverse Events', the SAE report must be sent to the IRB within 5 calendar days of the event. The IRB requires a C PIMS SAE report be submitted electronically to the SAE Office as follows:

The report should contain the following information:

Fields populated from PIMS:

- Subject's initials
- Medical record number
- Disease/histology (if applicable)
- Protocol number and title

Data needing to be entered:

- The date the adverse event occurred
- The adverse event
- The grade of the event
- Relationship of the adverse event to the treatment (drug, device, or intervention)
- If the AE was expected
- The severity of the AE
- The intervention
- Detailed text that includes the following
    - An explanation of how the AE was handled
    - A description of the subject's condition
    - Indication if the subject remains on the study
- If an amendment will need to be made to the protocol and/or consent form

- If the SAE is an Unanticipated Problem

The PI's signature and the date it was signed are required on the completed report.

The CRDB SAE report should be completed as per above instructions. If appropriate, the report will be forwarded to the FDA by the SAE staff through the IND Office.

17.2.1 Special SAE Reporting

Specific Liver Function Abnormalities

Wherever possible, timely confirmation of initial liver-related laboratory abnormalities should occur prior to the reporting of a potential Drug Induced Liver Injury (DILI) event. All occurrences of potential DILIs, meeting the defined criteria, must be reported as SAEs.

Potential DILI is defined as:

1. ALT or AST elevation > 3 times upper limit of normal (ULN) AND
2. Total bilirubin > 2 times ULN, without initial findings of cholestasis (elevated serum alkaline phosphatase) AND
3. No other immediately apparent possible causes of this elevation and hyperbilirubinemia, including, but not limited to, viral hepatitis, pre-existing chronic or acute liver disease, or the administration of other drug(s) known to be hepatotoxic.

17.2.2 SAE Reporting Procedures for BMS (MSK ONLY)

Serious Adverse Event Collection and Reporting

SAEs, whether related or not related to study drug, pregnancies, and overdosing of study drug(s) must be reported to BMS by MSK **within 24 hours of MSK's determination of the event**. SAEs must be recorded on the approved MSK SAE Report form; pregnancies on a Pregnancy Surveillance Form.

BMS SAE Email Address: Worldwide.Safety@BMS.com
BMS SAE Facsimile Number: 609-818-3804

If only limited information is initially available, follow-up reports are required. (Note: Follow-up SAE reports should include the same investigator term(s) initially reported.)

If an ongoing SAE changes in its intensity or relationship to study drug or if new information becomes available, a follow-up SAE report should be sent by MSK within

24 hours to the BMS (or designee) using the same procedure used for transmitting the initial SAE report. All SAEs should be followed to resolution or stabilization.

For studies conducted under an Investigator IND in the US, any event that is both serious and unexpected must be reported to the Food and Drug Administration (FDA) as soon as possible and no later than **7 days** (for a death or life-threatening event) **or 15 days** (for all other SAEs) after the investigator's or institution's initial receipt of the information. BMS will be provided with a simultaneous copy of all adverse events filed with the FDA at the contact email and fax number provided above.

SAEs will be reported using the MSK Clinical Research Database (CRDB) SAE form.

17.2.2 SAE Reporting Procedures for Nektar (MSK ONLY)

Serious Adverse Event Collection and Reporting

SAEs, whether related or not related to study drug, pregnancies, and overdosing of study drug(s) must be reported to Nektar by MSK **within 24 hours of MSK's determination of the event**. SAEs must be recorded on the approved MSK SAE Report form (pregnancies on a Pregnancy Surveillance Form), and transmitted to Nektar via e-mail or fax to the following address:

Nektar SAE Email Address: pharmacovigilance@Nektar.com
Nektar SAE Facsimile Number: 1-855-482-7233 or 1-415-482-5410

If only limited information is initially available, follow-up reports are required. (Note: Follow-up SAE reports should include the same investigator term(s) initially reported.)

If an change in its intensity or relationship to study drug or if new information becomes available, a follow-up SAE report should be sent by MSK within 24 hours to Nektar (or designee) using the same procedure used for transmitting the initial SAE report. All SAEs should be followed to resolution or stabilization.

Nektar will acknowledge all reports received from MSKCC within one business day.

17.2.3 SUSAR Reports

SUSAR Reports from BMS to MSK

In accordance with local regulations, BMS will notify investigators of all reported SAEs that are suspected (related to the investigational product) and unexpected (i.e., not previously described in the IB). An event meeting these criteria is termed a Suspected, Unexpected Serious Adverse Reaction (SUSAR).

Other important findings which may be reported by BMS as a SUSAR include: increased frequency of a clinically significant expected SAE, an SAE considered associated with study procedures that could modify the conduct of the study, lack of efficacy that poses significant hazard to study subjects, clinically significant safety finding from a nonclinical (e.g., animal) study, important safety recommendations from a study data monitoring committee, or sponsor decision to end or temporarily halt a clinical study for safety reasons.

Upon receiving a SUSAR from BMS, the investigator must review and retain the susar with the IB. Where required by local regulations or when there is a central IRB/IEC for the study, the investigator will submit the SUSAR to the appropriate IRB/IEC. The investigator and IRB/IEC will determine if the informed consent requires revision. The investigator should also comply with the IRB/IEC procedures for reporting any other safety information.

In addition, suspected serious adverse reactions (whether expected or unexpected) shall be reported by BMS to the relevant competent health authorities in all concerned countries according to local regulations (either as expedited and/or in aggregate reports).

SUSAR Reports from Nektar to MSK

In accordance with safety regulations, Nektar will notify all relevant health authorities and investigators involving in NKTR-214 clinical studies of all SUSARs reported from all clinical studies using NKTR-214 as an IMP (investigational medicinal product). Nektar will forward these SUSAR reports from other NKTR-214 clinical studies to MSKCC at the contact address below. Upon receiving a SUSAR report from Nektar, the investigators of this study must review and retain the SUSAR with the IB. Where required by local regulations or when there is a local or central IRB/IEC for the study, the investigators will submit the SUSAR to the appropriate IRB/IEC. The investigators and IRB/IEC will determine if the informed consent requires revision. The investigator should also comply with the IRB/IEC procedures for reporting any other safety information.

MSKCC is responsible for submitting any SUSAR or IND safety reports from this study to the FDA (under the IND 136287). Nektar shall be provided with a simultaneous copy of all SUSARs/IND Safety reports filed with the FDA at the contact email or fax number provided in Section 17.2.2

18.0 INFORMED CONSENT PROCEDURES

Before protocol-specified procedures are carried out, consenting professionals will explain full details of the protocol and study procedures as well as the risks involved to participants prior to their inclusion in the study. Participants will also be informed that they are free to withdraw from the study at any time. All participants must sign an IRB/PB-approved consent form indicating their consent to participate. This consent form meets the requirements of the Code of Federal Regulations and the Institutional Review Board/Privacy Board of this Center. The consent form will include the following:

1. The nature and objectives, potential risks and benefits of the intended study.
2. The length of study and the likely follow-up required.
3. Alternatives to the proposed study. (This will include available standard and investigational therapies. In addition, patients will be offered an option of supportive care for therapeutic studies.)
4. The name of the investigator(s) responsible for the protocol.
5. The right of the participant to accept or refuse study interventions/interactions and to withdraw from participation at any time.

Before any protocol-specific procedures can be carried out, the consenting professional will fully explain the aspects of patient privacy concerning research specific information. In addition to signing the IRB Informed Consent, all patients must agree to the Research Authorization component of the informed consent form.

Each participant and consenting professional will sign the consent form. The participant must receive a copy of the signed informed consent form.

19.0 REFERENCES

1. Lindberg, R.D., et al., *Conservative surgery and postoperative radiotherapy in 300 adults with soft-tissue sarcomas.* Cancer, 1981. **47**(10): p. 2391-7.
2. Weitz, J., C.R. Antonescu, and M.F. Brennan, *Localized extremity soft tissue sarcoma: improved knowledge with unchanged survival over time.* J Clin Oncol, 2003. **21**(14): p. 2719-25.
3. Billingsley, K.G., et al., *Pulmonary metastases from soft tissue sarcoma: analysis of patterns of diseases and postmetastasis survival.* Ann Surg, 1999. **229**(5): p. 602-10; discussion 610-2.
4. Van Glabbeke, M., et al., *Prognostic factors for the outcome of chemotherapy in advanced soft tissue sarcoma: an analysis of 2,185 patients treated with anthracycline-containing first-line regimens--a European Organization for Research and Treatment of Cancer Soft Tissue and Bone Sarcoma Group Study.* J Clin Oncol, 1999. **17**(1): p. 150-7.
5. Brennan, M.F., *Management of Soft Tissue Sarcoma*. 2003, New York: Springer.
6. Coley, W.B., *II. Contribution to the Knowledge of Sarcoma.* Ann Surg, 1891. **14**(3): p. 199-220.

7. Gatti, R.A. and R.A. Good, *Occurrence of malignancy in immunodeficiency diseases. A literature review.* Cancer, 1971. **28**(1): p. 89-98.
8. Sorbye, S.W., et al., *Prognostic impact of lymphocytes in soft tissue sarcomas.* PLoS One, 2011. **6**(1): p. e14611.
9. Berghuis, D., et al., *Pro-inflammatory chemokine-chemokine receptor interactions within the Ewing sarcoma microenvironment determine CD8(+) T-lymphocyte infiltration and affect tumour progression.* J Pathol, 2011. **223**(3): p. 347-57.
10. Zhang, L., et al., *Intratumoral T cells, recurrence, and survival in epithelial ovarian cancer.* N Engl J Med, 2003. **348**(3): p. 203-13.
11. Balachandran, V.P., et al., *Imatinib potentiates antitumor T cell responses in gastrointestinal stromal tumor through the inhibition of Ido.* Nat Med, 2011. **17**(9): p. 1094-100.
12. Brinkrolf, P., et al., *A high proportion of bone marrow T cells with regulatory phenotype (CD4+CD25hiFoxP3+) in Ewing sarcoma patients is associated with metastatic disease.* Int J Cancer, 2009. **125**(4): p. 879-86.
13. Nakayama, T., et al., *Natural course of desmoid-type fibromatosis.* J Orthop Sci, 2008. **13**(1): p. 51-5.
14. Chou, A.J., et al., *Addition of muramyl tripeptide to chemotherapy for patients with newly diagnosed metastatic osteosarcoma: a report from the Children's Oncology Group.* Cancer, 2009. **115**(22): p. 5339-48.
15. Edmonson, J.H., et al., *Phase II study of recombinant gamma-interferon in patients with advanced nonosseous sarcomas.* Cancer Treat Rep, 1987. **71**(2): p. 211-3.
16. Edmonson, J.H., et al., *Phase II study of recombinant alfa-2a interferon in patients with advanced bone sarcomas.* Cancer Treat Rep, 1987. **71**(7-8): p. 747-8.
17. Ito, H., et al., *Effect of human leukocyte interferon on the metastatic lung tumor of osteosarcoma: case reports.* Cancer, 1980. **46**(7): p. 1562-5.
18. Schwinger, W., et al., *Feasibility of high-dose interleukin-2 in heavily pretreated pediatric cancer patients.* Ann Oncol, 2005. **16**(7): p. 1199-206.
19. Robbins, P.F., et al., *Tumor regression in patients with metastatic synovial cell sarcoma and melanoma using genetically engineered lymphocytes reactive with NY-ESO-1.* J Clin Oncol, 2011. **29**(7): p. 917-24.
20. Tawbi, H.A.-H., et al., *Safety and efficacy of PD-1 blockade using pembrolizumab in patients with advanced soft tissue (STS) and bone sarcomas (BS): Results of SARC028--A multicenter phase II study.* ASCO Meeting Abstracts, 2016. **34**(15_suppl): p. 11006.
21. George, S., et al., *Phase 2 study of nivolumab in metastatic leiomyosarcoma of the uterus.* ASCO Meeting Abstracts, 2016. **34**(15_suppl): p. 11007.
22. Hanzly, M., et al., *High-dose interleukin-2 therapy for metastatic renal cell carcinoma: a contemporary experience.* Urology, 2014. **83**(5): p. 1129-34.
23. Topalian, S.L. and A.H. Sharpe, *Balance and imbalance in the immune system: life on the edge.* Immunity, 2014. **41**(5): p. 682-4.
24. Payne, R., et al., *Durable responses and reversible toxicity of high-dose interleukin-2 treatment of melanoma and renal cancer in a Community Hospital Biotherapy Program.* J Immunother Cancer, 2014. **2**: p. 13.
25. Boyman, O., et al., *Homeostatic maintenance of T cells and natural killer cells.* Cell Mol Life Sci, 2012. **69**(10): p. 1597-608.
26. Boyman, O. and J. Sprent, *The role of interleukin-2 during homeostasis and activation of the immune system.* Nat Rev Immunol, 2012. **12**(3): p. 180-90.
27. Daud, A.I., et al., *Programmed Death-Ligand 1 Expression and Response to the Anti-Programmed Death 1 Antibody Pembrolizumab in Melanoma.* J Clin Oncol, 2016. **34**(34): p. 4102-4109.
28. Daud, A.I., et al., *Tumor immune profiling predicts response to anti-PD-1 therapy in human melanoma.* J Clin Invest, 2016. **126**(9): p. 3447-52.
29. Brayer, J. and M. Fishman, *Regression of metastatic clear cell kidney cancer with interleukin-2 treatment following nivolumab (anti-PD-1) treatment.* J Immunother, 2014. **37**(3): p. 187-91.

30. Wong MKK, M.M., McDermott DF, et al. , *Overall survival of metastatic melanoma patients treated with HD IL-2 followed by immune checkpoint blockade of the CTLA-4 or the PD-1 pathways: analysis of data on the current use of HD IL-2.* J Immunother Cancer, 2015. **3**((Suppl 2)): p. 359.
31. Algazi A, T.K., Takamura KT, et al. , *Intratumoral electroporation of plasmid IL-12 can prime response to anti-PD1/PD-L1 blockade in patients with Stage III/IV-M1a melanoma.* AACR Meeting Abstracts, 2016.
32. Spranger, S., et al., *Mechanism of tumor rejection with doublets of CTLA-4, PD-1/PD-L1, or IDO blockade involves restored IL-2 production and proliferation of CD8(+) T cells directly within the tumor microenvironment.* J Immunother Cancer, 2014. **2**: p. 3.
33. West, E.E., et al., *PD-L1 blockade synergizes with IL-2 therapy in reinvigorating exhausted T cells.* J Clin Invest, 2013. **123**(6): p. 2604-15.
34. Heinrich, M.C., et al., *Kinase mutations and imatinib response in patients with metastatic gastrointestinal stromal tumor.* J Clin Oncol, 2003. **21**(23): p. 4342-9.
35. Topalian, S.L., et al., *Safety, activity, and immune correlates of anti-PD-1 antibody in cancer.* N Engl J Med, 2012. **366**(26): p. 2443-54.
36. Eisenhauer, E.A., et al., *New response evaluation criteria in solid tumours: revised RECIST guideline (version 1.1).* Eur J Cancer, 2009. **45**(2): p. 228-47.
37. Wolchok, J.D., et al., *Guidelines for the evaluation of immune therapy activity in solid tumors: immune-related response criteria.* Clin Cancer Res, 2009. **15**(23): p. 7412-20.
38. Postow, M.A., et al., *Nivolumab and ipilimumab versus ipilimumab in untreated melanoma.* N Engl J Med, 2015. **372**(21): p. 2006-17.
39. van der Graaf, W.T., et al., *Pazopanib for metastatic soft-tissue sarcoma (PALETTE): a randomised, double-blind, placebo-controlled phase 3 trial.* Lancet, 2012. **379**(9829): p. 1879-86.

20.0 APPENDICES

Appendix 1 Multicenter Addendum

Appendix 2 Patient Wallet Card

Appendix 3 Management Algorithms

Appendix 4 CT Biopsy Dose Analysis

Appendix 5 MUGA Normal-Organ Radiation Dosimetry

Appendix 6 Pharmacy Manual

Appendix 7 Lab Manual
