# Supplementary material for: Pilot study of bempegaldesleukin in combination with nivolumab in patients with metastatic sarcoma
Source: Nat Commun. 2022 Jun 16;13:3477. doi: 10.1038/s41467-022-30874-8 (PMC9203519; doi:10.1038/s41467-022-30874-8)
Supplement: Supplementary file 2 — Description of Additional Supplementary Files [file 41467_2022_30874_MOESM2_ESM.pdf]

Supplementary Data Legends

Supplementary Data 1: Sample attributes. Values are listed for each sample with the first column indicating the patient from which the sample was derived and the second column indicating the sample time point followed by IHC values, trial cohort, sample purity, and the identifying names used for exome, RNAseq or TCRseq files. Source data are provided as a Source Data file.

Supplementary Data 2: Models of partial response with cohort and IHC immune markers. Partial response versus all other samples was modeled by each IHC immune marker separately with cohort as a covariate. Samples were subsetted by time point. For patients with samples at both time points, the difference in percent positive immune cells was used to model partial response. Nominal p-value of the full model is listed as well as the nominal p-value of each marker within the model. Source data are provided as a Source Data file.

Supplementary Data 3: Differentially expressed genes between partial responders and non-partial responders. The first column shows the Ensembl ID followed by the gene symbol in the second column. The next three columns are derived from sleuth where b is the effect size (beta), pval is nominal p-value and qval is the q-value, which is corrected for multiple tests. Genes were considered differentially expressed if the q-value < 0.05. Source data are provided as a Source Data file.
