# Supplementary material for: Pilot study of bempegaldesleukin in combination with nivolumab in patients with metastatic sarcoma
Source: Nat Commun. 2022 Jun 16;13:3477. doi: 10.1038/s41467-022-30874-8 (PMC9203519; doi:10.1038/s41467-022-30874-8)
Supplement: Supplementary file 6 — Reporting Summary [file 41467_2022_30874_MOESM6_ESM.pdf]

Corresponding author(s): Sandra D'Angelo

Last updated by author(s): Apr 27, 2022

Reporting Summary

Nature Portfolio wishes to improve the reproducibility of the work that we publish. This form provides structure for consistency and transparency in reporting. For further information on Nature Portfolio policies, see our Editorial Policies and the Editorial Policy Checklist.

Statistics

For all statistical analyses, confirm that the following items are present in the figure legend, table legend, main text, or Methods section.

n/a	Confirmed	
☐	☒	The exact sample size (*n*) for each experimental group/condition, given as a discrete number and unit of measurement
☐	☒	A statement on whether measurements were taken from distinct samples or whether the same sample was measured repeatedly
☐	☒	The statistical test(s) used AND whether they are one- or two-sided<br>*Only common tests should be described solely by name; describe more complex techniques in the Methods section.*
☐	☒	A description of all covariates tested
☐	☒	A description of any assumptions or corrections, such as tests of normality and adjustment for multiple comparisons
☐	☒	A full description of the statistical parameters including central tendency (e.g. means) or other basic estimates (e.g. regression coefficient) AND variation (e.g. standard deviation) or associated estimates of uncertainty (e.g. confidence intervals)
☐	☒	For null hypothesis testing, the test statistic (e.g. *F*, *t*, *r*) with confidence intervals, effect sizes, degrees of freedom and *P* value noted<br>*Give P values as exact values whenever suitable.*
☒	☐	For Bayesian analysis, information on the choice of priors and Markov chain Monte Carlo settings
☒	☐	For hierarchical and complex designs, identification of the appropriate level for tests and full reporting of outcomes
☐	☒	Estimates of effect sizes (e.g. Cohen's *d*, Pearson's *r*), indicating how they were calculated

Our web collection on statistics for biologists contains articles on many of the points above.

Software and code

Policy information about availability of computer code

Data collection	Whole exome sequencing was performed on HiSeq 4000 or HiSeq 2500 in rapid mode in a PE100 run. RNA sequencing was performed on HiSeq 4000 in a PE100 run. TCR sequencing was performed using the Immunoverse kit from ArcherDX (catalog #DB0219) and sequenced on an Illumina platform. ArcherDX delivered trimmed and deduplicated fastqs. IHC was performed on paraffin-embedded tumor specimens for PD-L1, CD8, FOXP3, CD68, Ki67 and PD-1 using antibodies from Mosaic Laboratories.
Data analysis	Code for processing whole exome sequencing data can be found https://ccstempo.netlify.app/. Many previously published algorithms are included in these pipelines and further information and versions can be found in the Methods section of the manuscript.Code for processing RNA-seq data and for subsequent analysis throughout the manuscript can be found at https://github.com/mskcc/ImmunoSarc.

For manuscripts utilizing custom algorithms or software that are central to the research but not yet described in published literature, software must be made available to editors and reviewers. We strongly encourage code deposition in a community repository (e.g. GitHub). See the Nature Portfolio guidelines for submitting code & software for further information.

Data

Policy information about availability of data

All manuscripts must include a data availability statement. This statement should provide the following information, where applicable:

- Accession codes, unique identifiers, or web links for publicly available datasets
- A description of any restrictions on data availability
- For clinical datasets or third party data, please ensure that the statement adheres to our policy

De-identified exome sequencing data from patients treated at Memorial Sloan Kettering Cancer Center have been deposited in the NCBI dbGaP archive under accession number phs001783 [http://www.ncbi.nlm.nih.gov/projects/gap/cgi-bin/study.cgi?study_id=phs001783.v3.p1]. De-identified exome sequencing data from

patients treated at MD Anderson Cancer Center and all de-identified RNA sequencing and TCR sequencing data have been deposited in the NCBI dbGaP archive under accession number phs002852 [https://www.ncbi.nlm.nih.gov/projects/gap/cgi-bin/study.cgi?study_id=phs002852.v1.p1]. The data are available under controlled access, which can be obtained through dbGaP upon reasonable request. The raw sequencing data from 3 patients are protected and are not deposited in dbGaP because they did not consent to future use. Mapping of each sample, along with IHC values, can be found in Supplementary Data 1. The study protocol is available as Supplementary Note 1 in the Supplementary Information file. The source data for each figure can be found at https://github.com/mskcc/ImmunoSarc; source data are also provided in the Source Data file. The remaining data are available within the Article and Supplementary Information.

Field-specific reporting

Please select the one below that is the best fit for your research. If you are not sure, read the appropriate sections before making your selection.

☒ Life sciences ☐ Behavioural & social sciences ☐ Ecological, evolutionary & environmental sciences

For a reference copy of the document with all sections, see nature.com/documents/nr-reporting-summary-flat.pdf

Life sciences study design

All studies must disclose on these points even when the disclosure is negative.

Sample size

A sample size of 10 patients is planned for seven of the histological cohorts. If 2 or more confirmed responses are observed among the 10 patients in an arm, the drug combination will be claimed to be positive and worthy further investigation for that arm. A sample size of 15 is planned for each of the other two cohorts (in order to account for the greater sample heterogeneity in these cohorts). If 3 or more confirmed responses are observed among the 15 patients in each of these two arms, the drug combination will be claimed to be positive and worthy further investigation for that arm. The probability of observing a positive result for an individual arm and the probability of observing at least one positive arm among the six arms are provided in the protocol page 71. With the small sample size planned for the pilot study, we acknowledge that there will be a 52% probability that at least one cohort will look promising with respect to ORR even if all cohorts have a true overall response rate of 5%. We will interpret the study results cautiously when planning future studies and may follow up promising findings with a larger phase 2 study in the future.

Data exclusions

There were no data exclusions.

Replication

This was designed as a pilot study, signal seeking with intention to expand enrollment if appropriate.

Randomization

Study was non-randomized.

Blinding

Study design was non-blinded.

Reporting for specific materials, systems and methods

We require information from authors about some types of materials, experimental systems and methods used in many studies. Here, indicate whether each material, system or method listed is relevant to your study. If you are not sure if a list item applies to your research, read the appropriate section before selecting a response.

Materials & experimental systems

n/a	Involved in the study	
☐	☒	Antibodies
☒	☐	Eukaryotic cell lines
☒	☐	Palaeontology and archaeology
☒	☐	Animals and other organisms
☐	☒	Human research participants
☐	☒	Clinical data
☒	☐	Dual use research of concern

Methods

n/a	Involved in the study	
☒	☐	ChIP-seq
☒	☐	Flow cytometry
☒	☐	MRI-based neuroimaging

Antibodies

Antibodies used

PD-L1 (rabbit clone 28-8), CD8 (mouse clone C8/144B), CD68 (mouse clone KP1), FOXP3 (mouse clone 236A/E7), Ki67 (clone MIB-1) and PD-1 (rabbit clone EPR48772) by Mosaic laboratories. Catalog numbers are included in parentheses.

Validation

Mosaic Laboratories performs validation on their products. Statement from Mosaic Labs: "Immunohistochemistry (IHC) combines immunological and enzymatic techniques to localize and evaluate antigen expression in tissue sections. Single-stain immunohistochemistry may be evaluated by pathologist light microscopic review or image analysis. Mosaic's IHC assays are focused on immuno-oncology, immunology, signal-transduction pathways and relevant targets for cancer therapy. Mosaic has experience testing over a thousand antibodies for IHC and is validating numerous new assays on a rolling basis" (https://www.mosaiclabs.com/ihc/).

Human research participants

Policy information about studies involving human research participants

Population characteristics	Eligible patients were ≥12 years and had advanced or metastatic sarcoma measurable per RECIST v1.1, ECOG performance status of 0 or 1, an estimated life expectancy of ≥3 months, previous receipt of ≥1 systemic therapy for metastatic disease (if applicable), and adequate kidney, liver, and bone marrow function. A sample size of 10 patients was planned for 7 of the histological cohorts (LMS, UPS/MFS, chondrosarcoma, DDLPS, osteosarcoma, angiosarcoma and ASPS), and a sample size of 15 patients was planned for the SBRCT/synovial sarcoma and other cohorts to account for their heterogeneity. A minimum of 28 days was required between any previous systemic therapy and initiation of bempegaldesleukin and nivolumab. Key exclusion criteria were active brain metastases or history of autoimmune diseases. Prior therapy with anti-PD-1 was permitted. A full list of patient characteristics is presented in Table 1.
Recruitment	The clinical trial will be listed on the clinicaltrials.gov website and on the websites of participating institutions. All eligible patients, regardless of sex and race, will be approached for participation. The investigators are aware of the NIH policy concerning inclusion of women and minorities in clinical research populations. Participants were recruited at regular oncology visits without regard to sex or race/ethnicity. Participants reflect the makeup of the MSK and MDA catchment areas and patient populations.
Ethics oversight	Protocol was approved by Memorial Sloan Kettering Cancer Center and MD Anderson Cancer Center (Methods text has been updated).<br><br>Patients ≥18 years of age had to be capable, willing, and able to provide written informed consent/assent. For patients < 18 years of age, their parents or legal guardians had to sign a written informed consent. Assent, when appropriate, was obtained according to institutional guidelines.

Note that full information on the approval of the study protocol must also be provided in the manuscript.

Clinical data

Policy information about clinical studies

All manuscripts should comply with the ICMJE guidelines for publication of clinical research and a completed CONSORT checklist must be included with all submissions.

Clinical trial registration	NCT03282344
Study protocol	Study protocol provided
Data collection	A total of 84 patients with selected locally advanced or metastatic high-grade sarcoma were enrolled from October 6, 2017 - January 28, 2020. Study was conducted at Memorial Sloan Kettering Cancer Center and MD Anderson Cancer Center.
Outcomes	The primary endpoint was confirmed ORR within each histology cohort based on RECIST v1.1 during protocol-directed treatment. Secondary outcomes were toxicity, clinical benefit rate, duration of response, PFS, OS, and duration of treatment.
